# Intracellular Diversity of the V4 and V9 Regions of the 18S rRNA in Marine Protists (Radiolarians) Assessed by High-Throughput Sequencing

**DOI:** 10.1371/journal.pone.0104297

**Published:** 2014-08-04

**Authors:** Johan Decelle, Sarah Romac, Eriko Sasaki, Fabrice Not, Frédéric Mahé

**Affiliations:** 1 Sorbonne Universités, UPMC Univ. Paris 06, UMR 7144, Station Biologique de Roscoff, Roscoff, France; 2 CNRS, UMR 7144, Station Biologique de Roscoff, Roscoff, France; 3 Gregor Mendel Institute of Molecular Plant Biology, Vienna, Austria; 4 Department of Ecology, University of Kaiserslautern, Kaiserslautern, Germany; Laval University, Canada

## Abstract

Metabarcoding is a powerful tool for exploring microbial diversity in the environment, but its accurate interpretation is impeded by diverse technical (e.g. PCR and sequencing errors) and biological biases (e.g. intra-individual polymorphism) that remain poorly understood. To help interpret environmental metabarcoding datasets, we investigated the intracellular diversity of the V4 and V9 regions of the 18S rRNA gene from Acantharia and Nassellaria (radiolarians) using 454 pyrosequencing. Individual cells of radiolarians were isolated, and PCRs were performed with generalist primers to amplify the V4 and V9 regions. Different denoising procedures were employed to filter the pyrosequenced raw amplicons (Acacia, AmpliconNoise, Linkage method). For each of the six isolated cells, an average of 541 V4 and 562 V9 amplicons assigned to radiolarians were obtained, from which one numerically dominant sequence and several minor variants were found. At the 97% identity, a diversity metrics commonly used in environmental surveys, up to 5 distinct OTUs were detected in a single cell. However, most amplicons grouped within a single OTU whereas other OTUs contained very few amplicons. Different analytical methods provided evidence that most minor variants forming different OTUs correspond to PCR and sequencing artifacts. Duplicate PCR and sequencing from the same DNA extract of a single cell had only 9 to 16% of unique amplicons in common, and alignment visualization of V4 and V9 amplicons showed that most minor variants contained substitutions in highly-conserved regions. We conclude that intracellular variability of the 18S rRNA in radiolarians is very limited despite its multi-copy nature and the existence of multiple nuclei in these protists. Our study recommends some technical guidelines to conservatively discard artificial amplicons from metabarcoding datasets, and thus properly assess the diversity and richness of protists in the environment.

## Introduction

High-throughput sequencing of phylogenetic markers (metabarcoding) is becoming the gold standard approach for exploring microbial diversity in the environment [1], [2], [3]. The presence of the 18S rRNA across all eukaryotes, its extensive occurrence in public reference databases and the availability of generalist primers make this gene the best universal marker available to date for eukaryotes [4], [5]. Metabarcoding of microbial eukaryotes typically targets the short variable regions V4 and V9 of the 18S rRNA gene [2], [3]. From the reads generated (amplicons), definition of operational taxonomic units (OTUs) is classically used not only to identify taxonomic entities and describe community structure (e.g. diversity and richness), but also to assess the extent of the so-called “rare biosphere” [6], [7]. Different identity thresholds, ranging between 95% and 99%, have been used to delineate OTUs in various environmental surveys [8], [9], [10].

However, when using the 18S rRNA marker, heterogeneous evolutionary rates between taxa, intracellular polymorphism, rDNA copy number variation and presence of pseudogenes are potentially important, yet poorly understood, shortcomings for properly evaluating community composition [11], [12], [13]. For instance, intra-individual polymorphism of the 18S rRNA has been reported in different eukaryotes like benthic Foraminifera [14]. Pseudogenes, defined as non-functional gene copies [15], have been also found in different eukaryotic taxa, including metazoans and protists [16], [17]. Moreover, the ribosomal array can be composed of “alien” copies resulting from lateral transfer of one sequence from unrelated species. Recently, such lateral transfer of rRNA gene, though considered unique to prokaryotes [18], has been reported for the first time in eukaryotes (i.e. ciliates) [19]. Thus, considering the sequencing depth of the next generation technologies, the different copies, pseudogenes and other variants of the 18S rRNA of each organism, all can be potentially detected in metabarcoding surveys, and consequently lead to inflated diversity metrics by increasing the number of predicted OTUs. In this context, prior to studying specific taxa from metabarcoding of communities, it appears to be necessary to explore the genetic variation of the targeted barcode in single species or even in individual cells. Such calibration is paramount to carefully interpret the flow of sequences obtained from complex natural communities.

In addition to these biological concerns, PCR artifacts and sequencing errors that scale with the sequencing effort are known to artificially inflate diversity estimates [20], [21]. Discriminating between natural amplicons and technical artifacts is definitively a challenge that has to be addressed for accurate interpretation of large datasets in molecular ecology.

In this study, we investigated the intracellular diversity of the ribosomal barcodes V4 and V9 in eukaryotes using 454 pyrosequencing. We focused on two radiolarian taxa, Acantharia and Nassellaria, which are heterotrophic marine protists, from which no genomic data is available to date. Their large cells (100–500 µm in diameter) are supported by a mineral skeleton and can contain several nuclei [22]. Acantharia and Nassellaria are important components of planktonic communities due to their abundance, predation, contribution to the vertical flux of organic matter, and indirectly as primary producers through symbiosis with microalgae [23], [24], [25], [26]. These uncultivated planktonic organisms have also a widespread distribution in marine environments since numerous environmental 18S rRNA sequences have been found from diverse habitats, including coastal [27], deep [28], [29], polar [30], [31], and anoxic waters [32], [33], [34]. The ecological and biogeochemical significance of Acantharia and Nassellaria make them key players in pelagic ecosystems, and stress the need to define a proper analytical procedure to explore their molecular diversity in the environment.

## Materials and Methods

### Single-cell collection, PCR amplification and pyrosequencing of the V4 and V9 regions

**Figure 1:**
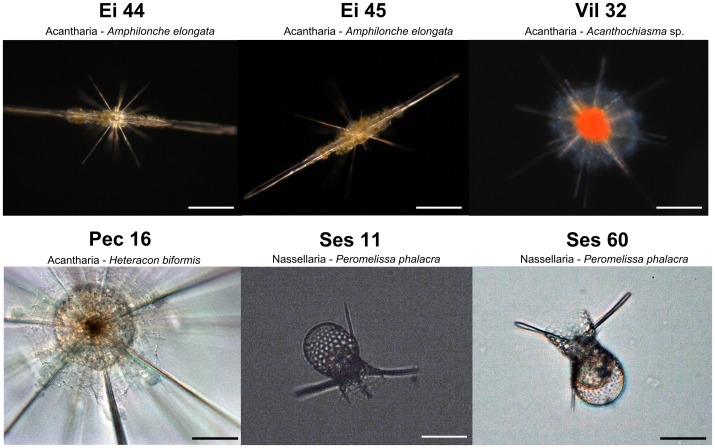
Light microscopy pictures of individual acantharian (n = 4) and nassellarian (n = 2) cells of 100–300 µm in size, isolated in the Red Sea - Gulf of Eilat (the acantharians Ei 44 and Ei 45, *Amphilonche elongata*; scale bars = 50 µm), Mediterranean Sea (the acantharians Vil 32 - *Heteracon biformis* and Pec 16 - *Staurolithium* sp.; scale bars = 50 and 20 µm, respectively) and Pacific Ocean - Sesoko Island (the nassellarians Ses 11 and Ses 60, *Peromelissa phalacra*; scale bars = 30 µm).

Radiolarian cells were collected in the Gulf of Eilat, Red Sea (the acantharians Ei 44 and Ei 45: *Amphilonche elongata*), Mediterranean Sea (the acantharians Vil 32 and Pec 16: *Staurolithium* sp. and *Heteracon biformis*, respectively) and Sesoko Island, NW Pacific Ocean (the nassellarians Ses 11 and Ses 60: *Peromelissa phalacra*) (see Figure 1; geographic coordinates of the locations are given in Materials S1). Individual cells were sampled from surface waters with a plankton net, micropipette isolated under a binocular microscope, and cleaned by several successive transfers into 0.22 µm-filtered seawater. No specific permits were required for the field sites, as the locations are not privately-owned or protected in any way (international oceanic waters), and the studied organisms did not involve endangered or protected species. Cells labeled Ei 44 and Ei 45 (*A. elongata*), and Ses 11 and Ses 60 (*P. phalacra*), belonging to the same acantharian and nassellarian morphospecies, respectively, are considered hereafter as biological replicates. The four acantharian morphospecies belong to the highly divergent phylogenetic clades C (Pec 16, *H. biformis*), D (Vil 32, *Staurolithium* sp.), and F (Ei 44 and Ei 45, *A. elongata*) [35].

DNA from each single cell was extracted as described in [35]. The V4 (ca. 380 bp) and V9 (ca. 130 bp) regions were PCR-amplified with eukaryote-specific primers that are regularly used in environmental protist surveys [3 and 2, respectively]. Because direct PCRs with 25 cycles yielded visible bands on agarose gel, Whole Genome Amplification (WGA) or nested PCR were not required to ensure the amplification of the V4 and V9 regions. Each sample was amplified in triplicate to increase the yield of amplicons, which were subsequently pooled and purified using the NucleoSpin Extract II kit (Macherey-Nagel, Hoerdt, France). To obtain a similar number of amplicons for each sample, purified PCR products were quantified with the Quant-iT™ PicoGreen dsDNA kit (Invitrogen) and then mixed in equal concentrations. Finally, amplicons were sequenced with the 454 GS-FLX Titanium pyrosequencing technology [36] (see Materials S1 for methodological details). Prior to PCR amplification and sequencing, the DNA extracts from two cells (Ei 44 and Pec 16) were split into two separate sub-samples, considered hereafter as technical replicates (Ei 44-1–Ei 44-2 and Pec 16-1–Pec 16-2).

### Filtering and taxonomic assignation of 454 pyrosequencing amplicons

A three-step filtering method was adopted to eliminate ambiguous amplicons: 1) denoising was performed with Acacia v1.52.b0 and AmpliconNoise v1.29 as described in Materials S1 [37], [38]; 2) amplicons not containing the exact distal primer sequence were removed; 3) chimeras were eliminated using UCHIME with default parameters after Acacia denoising [39]. Finally, primer sequences were trimmed off and amplicons were assigned to their closest hit in the Protist Ribosomal Reference database (PR2, version August 13, 2012 [4]) using ggsearch [40]. Amplicons corresponding to symbiotic microalgae (e.g. *Phaeocystis*) or contaminants (e.g. fungi, metazoans or other distant radiolarians) were not included in subsequent analyses. The filtered amplicons assigned to Acantharia or Nassellaria were aligned with Muscle, implemented in Seaview v.4.2.6 [41], and clustered at different identity thresholds, from 80% to 99%, using usearch ([42]; v.6.0.203_i86linux32). The V4 and V9 alignments of each individual cell did not exhibit ambiguous sections since amplicons are short and highly similar. The secondary structure of the V4 and V9 amplicons was predicted using the RNAfold server available on the Vienna RNA web servers (http://rna.tbi.univie.ac.at).

Another analytical approach, called the linkage method, was applied to infer dominant patterns and eliminate random noise [43]. Details of the methodological procedures are given in Materials S1. The raw V4 and V9 sequences have been deposited in the Short Read Archive under the accession number PRJEB4199.

## Results and Discussion

From each individual cell of Acantharia and Nassellaria, an average of 4,000 V4 and 2,380 V9 raw amplicons were obtained after pyrosequencing (Tables 1 and S1). Prior to assignation, two denoising algorithms, Acacia and AmpliconNoise, were used to filtered these amplicons. The total number of amplicons assigned to Acantharia or Nassellaria was highly variable between samples and denoising programs (Table 1). Using Acacia, from 4 to 957 V4 and 61 to 1,037 V9 amplicons were obtained. In general, AmpliconNoise retrieved more amplicons than Acacia, ranging from 386 to 2,594 for V4 and 30 to 1,080 for V9. This variability was also observed between technical replicates: in the sample Ei 44-2 we found six times more V4 amplicons than in Ei 44-1, despite comparable numbers of raw amplicons (7,197 and 6,942, respectively). Note that the acantharian Pec 16-2 and the nassellarian Ses 60 had no valid V4 amplicons after filtering in both denoising programs, probably because of the low initial number of raw amplicons obtained from these samples (1,081 and 1,088, respectively). Some of these raw amplicons were partial (distal primers were missing), and the majority were assigned to fungi, stramenopiles or metazoans that could correspond to preys or contaminants.

**Table 1:** Number of raw and filtered 454-pyrosequenced amplicons using the denoising programs Acacia [37] and AmpliconNoise [38].

			Radiolarian amplicons filtered with Acacia		Radiolarian amplicons filtered with AmpliconNoise		Number of OTUs at different identity cut-off levels (Acacia OTUs | AmpliconNoise OTUs)															Linkage method
Samples	Region	Raw amplicons	Total amplicons	Unique amplicons	Total amplicons	Unique amplicons	99%	98%	97%	96%	95%	94%	93%	92%	91%	90%	89%	88%	87%	86%	85%	Amplicon patterns
**Ei 44_1**	V4	6942	190	18 (15)	386	4 (2)	8|4	**4|4**	**3|4**	3|4	2|3	2|3	1|2	1|2	1|2	1|2	1|2	1|2	1|2	1|1	1|1	7
**Ei 44_2**	V4	7197	957	52 (37)	2594	3	19|3	**10|3**	**5|3**	2|3	2|1	1|1	1|1	1|1	1|1	1|1	1|1	1|1	1|1	1|1	1|1	21
**Ei 45**	V4	5142	907	76 (52)	1984	3	19|2	**5|2**	**2|2**	2|1	1|1	1|1	1|1	1|1	1|1	1|1	1|1	1|1	1|1	1|1	1|1	23
**Pec 16_1**	V4	2444	13	8 (5)	0	0	3|0	**2|0**	**1|0**	1|0	1|0	1|0	1|0	1|0	1|0	1|0	1|0	1|0	1|0	1|0	1|0	3
**Pec 16_2**	V4	1081	0	0	0	0	0|0	**0|0**	**0|0**	0|0	0|0	0|0	0|0	0|0	0|0	0|0	0|0	0|0	0|0	0|0	0|0	0
**Ses 11**	V4	5460	4	1	0	0	1|0	**1|0**	**1|0**	1|0	1|0	1|0	1|0	1|0	1|0	1|0	1|0	1|0	1|0	1|0	1|0	1
**Ses 60**	V4	1088	0	0	0	0	0|0	**0|0**	**0|0**	0|0	0|0	0|0	0|0	0|0	0|0	0|0	0|0	0|0	0|0	0|0	0|0	0
**Vil 32**	V4	2906	399	18 (15)	1226	1	1|1	**1|1**	**1|1**	1|1	1|1	1|1	1|1	1|1	1|1	1|1	1|1	1|1	1|1	1|1	1|1	1
**Ei 44_1**	V9	2746	656	18 (14)	30	3 (1)	10|3	**8|3**	**5|3**	3|3	3|3	3|3	2|2	2|2	2|2	2|2	1|1	1|1	1|1	1|1	1|1	7
**Ei 44_2**	V9	3899	1001	11 (5)	587	2	8|2	**7|2**	**4|2**	3|2	3|2	3|2	3|2	3|2	3|2	3|2	2|1	2|1	2|1	2|1	2|1	7
**Ei 45**	V9	1331	577	6 (3)	369	3 (1)	5|3	**4|3**	**3|3**	3|3	3|3	3|3	2|2	2|2	2|2	2|2	1|1	1|1	1|1	1|1	1|1	2
**Pec 16_1**	V9	1538	832	4 (3)	887	1	1|1	**1|1**	**1|1**	1|1	1|1	1|1	1|1	1|1	1|1	1|1	1|1	1|1	1|1	1|1	1|1	1
**Pec 16_2**	V9	1330	785	7(6)	808	1	2|1	**1|1**	**1|1**	1|1	1|1	1|1	1|1	1|1	1|1	1|1	1|1	1|1	1|1	1|1	1|1	1
**Ses 11**	V9	3913	61	1	64	1	1|1	**1|1**	**1|1**	1|1	1|1	1|1	1|1	1|1	1|1	1|1	1|1	1|1	1|1	1|1	1|1	1
**Ses 60**	V9	2488	108	3(2)	110	1	2|1	**2|1**	**1|1**	1|1	1|1	1|1	1|1	1|1	1|1	1|1	1|1	1|1	1|1	1|1	1|1	1
**Vil 32**	V9	1793	1037	1	1080	1	1|1	**1|1**	**1|1**	1|1	1|1	1|1	1|1	1|1	1|1	1|1	1|1	1|1	1|1	1|1	1|1	1

The number of singletons is indicated in brackets in the “Unique amplicons” column. OTUs were then calculated from each individual cells for the V4 (top) and V9 (bottom) regions of the ribosomal 18S rRNA gene. The numbers separated by a “|” symbol indicate the number of OTUs obtained after Acacia and AmpliconNoise denoising, respectively.

From a single cell, after denoising with Acacia and merging strictly identical amplicons, the number of unique amplicons was as high as 76 for the V4 and 18 for the V9. On average, 70% of these unique amplicons are singletons (amplicons occurring only once in the dataset). With AmpliconNoise, the number of unique amplicons was much lower (up to 4 V4 and 3 V9 amplicons from a single cell). AmpliconNoise appeared to be more stringent than Acacia since most singletons were discarded. However, this denoising algorithm did not retrieved V4 amplicons in Ses 11, Ses 60, Pec 16-1 and Pec 16-2. Among the radiolarians sampled in this study, Acantharia had more amplicons (total and unique) than Nassellaria, presumably because of the presence of multiple nuclei in acantharian cells. For the technical replicates Ei 44-1 and Ei 44-2, 18 and 52 unique V4 amplicons were found with Acacia, respectively, while the corresponding biological replicate Ei 45 had 76 unique V4 amplicons. Different number of unique amplicons between replicates were also observed with AmpliconNoise for the same cells, but to a lesser extent. These inconsistencies between replicates show that, in similar conditions, the PCR and sequencing steps can yield significantly different results in terms of amplicon number and diversity from the same morphospecies and even from the same DNA extract.

OTU-based approaches are classically used by microbial ecologists to assess species diversity and richness in the environment. Therefore, we investigated whether the various unique amplicons from single cells could form distinct OTUs. At the 97% identity level, a clustering threshold traditionally used in microbial diversity studies [8], [9], a single radiolarian cell can contain up to 5 V4 and 5 V9 radiolarian OTUs with Acacia, and up to 4 V4 and 3 V9 OTUs with the more stringent AmpliconNoise (Table 1). The highest numbers of OTUs were observed in acantharian cells (Ei 44 and Ei 45), presumably because of their higher number of unique amplicons. Notably, up to 3 V4 and 3 V9 OTUs were still found at 94% identity in the acantharian cells Ei 44 and Ei 45, both belonging to the species *Amphilonche elongata* (clade F). Furthermore, for both V4 and V9 regions, the number of OTUs was different between the biological and technical replicates, showing again the consequences of the fluctuating PCR and sequencing outcomes.

Overall, the distinct amplicon sequences and OTUs obtained from a single cell may indicate the existence of natural intracellular variability of the 18S rRNA in these radiolarians, more particularly in Acantharia. However, there was generally one numerically dominant amplicon sequence, and other amplicon sequences were in single or few copies (minor variants). Similarly, most amplicons from a single cell grouped in a single OTU, whereas other OTUs contained only few amplicons (Figure S1): between 88 and 100% of the total amplicons clustered in a single OTU at the 98% identity level. Thus, we argue that these acantharian and nassellarian morphospecies have one dominant 18S rRNA ribotype, but we cannot rule out at this stage the presence of distinct minor ribosomal variants.

**Figure 2:**
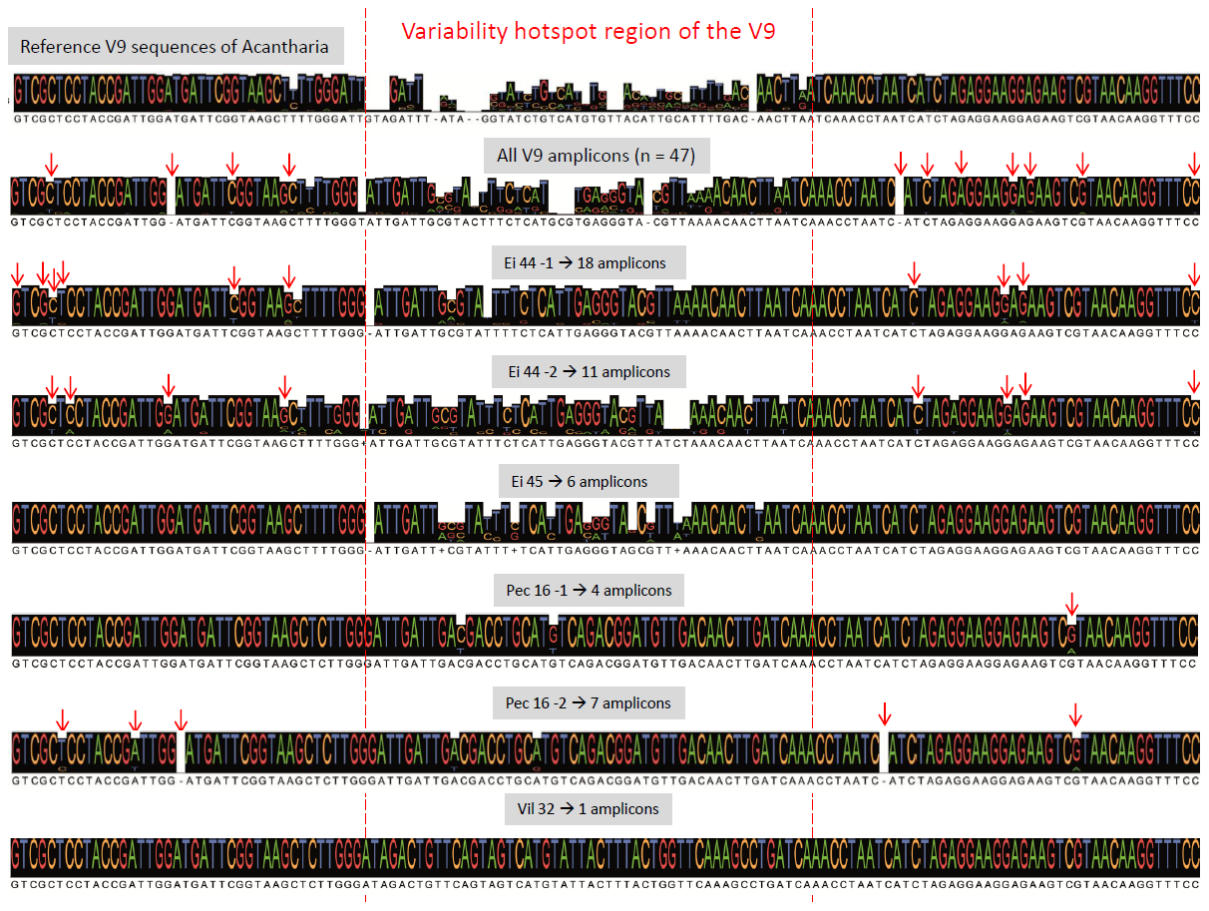
V9 alignment comparison between reference sequences of all the clades of Acantharia obtained in [35] (upper sequence consensus), and the unique V9 amplicons generated in this study filtered with Acacia (n = 47) from each individual acantharian cells (Ei 44-1, Ei 44-2, Ei 45, Pec 16-1, Pec 16-2 and Vil 32). The red dashed lines delimit the variability hotspot region of the V9 reference sequences, and the red arrows represent base substitutions (insertions or deletions) occurring outside the variability hotspot region in the pyrosequenced amplicons.

It is difficult to ascertain whether these minor variants represent natural intracellular variability, or whether they are artificially produced during the PCR and sequencing steps. This distinction is critical for carefully interpreting deep sequencing of environmental barcodes. Inspection of V4 and V9 alignments containing reference Sanger sequences and amplicons produced in this study (filtered with Acacia) revealed that most minor variants contained substitutions that seem to be randomly distributed and were not preferentially located in the variability hotspot region of reference sequences (Figure 2). For instance, Ei 44-2 had 11 unique V9 amplicons that represent 7 OTUs at 97%, but 9 of these amplicons contain nucleotide changes in regions that are conserved across all acantharian clades. Some of these substitutions might therefore represent PCR or sequencing errors that accumulate and can ultimately lead to the delineation of distinct OTUs when using high-level clustering thresholds. In addition, these substitutions can change the secondary structure of the V4 and V9 amplicon sequences found in individual cells (Figure S2). The secondary structure of the minor variants is generally different from the one of the dominant amplicon sequence, confirming that most substitutions are probably artificial.

**Figure 3:**
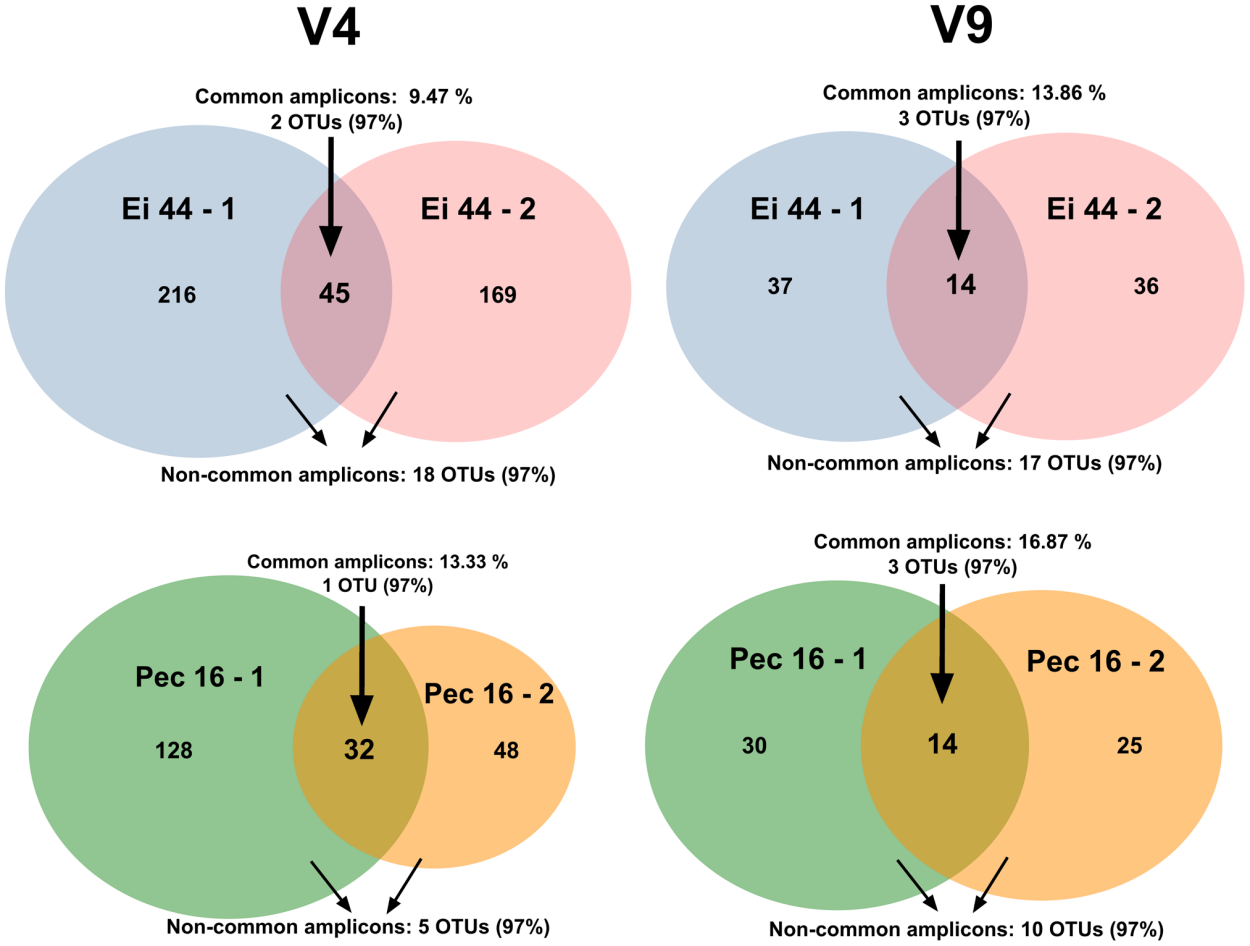
Venn diagrams showing the V4 and V9 amplicons shared between single-celled technical replicates (PCR and sequencing from the same DNA extract), or only found in one of the replicates. The number of OTUs formed with these common and non-common amplicons at 97% identity level is indicated.

In a further attempt to differentiate natural and artificial amplicons, we examined the amplicons that were shared between technical replicates (Ei 44-1 and Ei 44-2; Pec 16-1 and Pec 16-2). We choose to work with the radiolarian amplicons containing both primers without any additional quality-based filtering in order to compare between a simple noise removal procedure based on replicates and denoising programs (Acacia and AmpliconNoise). From the same single-cell DNA extract, we found that only 9 to 13% for the V4 and 13 to 16% for the V9 of the total unique amplicons were common between replicates (Figure 3). The majority of common amplicons were the most abundant ones, corresponding to the dominant ribotypes found previously, although some common amplicons were also present in low copy numbers (as low as 2 copies). The common amplicons formed 2 or 3 OTUs at the 97% clustering threshold in the acantharians Ei 44 and Pec 16 for both V4 and V9 regions (Figure 3 and Table S2). By contrast, amplicons found in only one of the replicates typically occurred in low abundance, most of them being singletons, and exhibited mutations in highly-conserved regions (outside the variability hotspot region; Figure S3). Remarkably, despite their low copy numbers, the non-common amplicons can form up to 18 OTUs at the 97% identity level, which is on average 4.7 times more than OTUs with common amplicons (Figure 3, Table S2). This additional line of evidence demonstrates that many amplicons are artificially produced during the PCR and sequencing steps, and are divergent enough to lead to an overestimation of OTU richness.

In addition to the more complex and computationally-intensive denoising algorithms [37], [38], [44], sample replication and cross-validation (selecting amplicons shared by replicates) could be an efficient and biologically meaningful method to differentiate technical artifacts from real biological signal in metabarcoding surveys. The identification of common amplicons between replicates also has the advantage of circumventing the use of arbitrary criteria and thresholds for inclusion/rejection of amplicons (e.g. minimum copy abundance and identity cut-off values), therefore allowing comparison between different environmental metabarcoding datasets. Considering the continually decreasing cost of sequencing and increasing size of new datasets, sample replication and cross-validation should be considered as an additional denoising step to ensure accurate estimates of environmental microbial diversity (“Replicate or lie” as claimed in [45]), though it remains to be properly tested on complex microbial communities.

To better understand the intracellular diversity of V4 and V9 regions, another approach, called the linkage method, was applied to the same single-cell datasets [43]. By detecting SNP combination patterns in sliding windows along the sequence, this method found that many pyrosequenced amplicons contained numerous random errors (Table S3). These amplicons that had unique patterns with no redundancy in each cell were therefore excluded for subsequent analyses (more details in File S1). The linkage method detected from 1 to 23 and 1 to 7 amplicon patterns in each acantharian and nassellarian cells for the V4 and V9, respectively (Table 1, Table S3). The results confirm that intra-individual polymorphism of the 18S rRNA is low or even absent in the acantharian and nassellarian cells. Between the technical replicates Ei 44-1 and Ei 44-2, three pairs of identical V4 and V9 sequences were recognized. For V4, one pair was numerically less abundant and more divergent compared to the other two pairs. These two pairs with one indel were also detected in a different cell of the same species (Ei 45, *Amphilonche elongata*), indicating that at least two different ribotypes of the 18S rRNA are present in this acantharian species. Pec 16-1 and Pec 16-2 shared a single and identical V9 amplicon, but the absence of V4 amplicons in Pec 16-2 prevented us from concluding that this acantharia has a single 18S rRNA ribotype. Similarly, the nassellarian species Ses 11 and Ses 60 had one unique V9 amplicon with a 1-substitution difference, but the number of amplicons obtained is not sufficient to define the ribotype number in these cells.

## Conclusion and Perspectives

Because of their ubiquitous distribution in marine environments and recurrent molecular detection from distinct environmental surveys, the radiolarian taxa Acantharia and Nassellaria will undoubtedly represent a significant part of sequence data in forthcoming environmental metabarcoding studies. The main goal of our study was to assess the intracellular variability of two genetic barcodes in these radiolarians by deep single-cell sequencing, and improve our ability to interpret metabarcoding datasets. Although several amplicon sequences and OTUs were found in a single cell, we assert that intra-individual polymorphism (defined as the divergence and relative abundance of the distinct copies) is limited in Acantharia and Nassellaria as cells contained a dominant ribotype with low-abundant variants. The ribosomal array seems therefore to evolve in concert despite its multi-copy and heterogeneous nature, and more particularly the presence of multiple nuclei in Acantharia. Based on the combination of alignment visualization, technical replicates, denoising algorithms, secondary structure prediction and a pattern-based method, we provided good evidence that many of the minor variants are artificially produced during the PCR and sequencing steps. More particularly, as also highlighted in other studies [46], we showed that amplicon sequencing approaches are not reproducible and require replicates for rigorous interpretation. Consequently, we recommend a conservative approach to discard artifacts from metabarcoding datasets: 1) two or three technical replicates (parallel PCR and sequencing steps from the same environmental sample), 2) a denoising procedure including cross-validation of amplicons between replicates, and 3) if working on specific taxon like Acantharia, alignment visualization with reference sequences to further remove ambiguous amplicons. The remaining amplicons that correspond to the dominant ribotypes of each cell should better reflect the natural diversity and richness of radiolarians in the environment. This conservative approach is critical to properly infer and compare diversity estimates of a particular taxon or a whole community across different samples, and so irrespective of the high-throughput sequencing technique. In addition, low error-rate polymerases, low cycle numbers and ideally PCR-free methods should be favored to alleviate technical biases in future metabarcoding studies.

Moreover, our approach allowed improving the 18S rRNA reference database of radiolarians by adding information about the number of ribotypes found in each species. For instance, we detected two ribotypes in the acantharian species *Amphilonche elongata* (Ei 44 and Ei 45). Although one ribotype is numerically more abundant than the other, both can be detected in environmental metabarcoding datasets. Similar approach should be adopted on more eukaryotic taxa to fine-tune the assignation of environmental barcodes and avoid inflating diversity estimates in metabarcoding surveys.

An obvious next step for radiolarians is to assess the rRNA copy number in single cells in order to estimate the abundance of these protists from metabarcoding datasets. For instance, a recent study using qPCR assays estimated the rRNA copy number per cell in benthic Foraminifera (i.e. 10,000–30,000) [47]. This allowed the establishment of normalization factors that were used to correctly determine abundance of species by removing “excess” of amplicons. Similar normalization has been also applied in bacteria based on the known copy number in reference genomes [48]. Alternatively, a single-copy gene could be selected as a barcode to assess the abundance of radiolarians in the environment, but the lack of genomic data remains the main barrier to select and validate such barcode among radiolarians.

Extending the approach conducted here on radiolarian single cells to other microbial taxa will help to define taxonomically meaningful and relevant genetic entities, but also to contribute to a better understanding of the potential and limitations of the 18S rRNA gene marker for environmental metabarcoding studies. A careful representation of the diversity and relative abundances of microbial organisms is critical for the establishment of biodiversity monitoring projects and the assessment of the impact of anthropogenic changes.

## Supporting Information

Figure S1 **Number and size of the V4 and V9 OTUs found in different individual cells of Radiolaria, based on amplicons filtered with the denoising program Acacia.** Each OTU is represented by a single color, and its number of amplicons is indicated in the bar.
(PDF)

Figure S2 **Predicted secondary structures of the V4 amplicons found in the sample Ei 44_1. The numbers indicate the abundance of the given amplicon.**
(PDF)

Figure S3 **V9 alignment comparison between reference sequences of all the clades of Acantharia (upper sequence consensus) and the common and non-common pyrosequenced amplicons obtained from technical replicates without Acacia denoising (Ei 44-1/Ei 44-2 and Pec 16-1 and Pec 16-2).** Compared to non-common amplicons, common amplicons tend to have fewer substitutions in highly-conserved regions.
(PDF)

Table S1 **Number of amplicons at the different consecutive filtering steps: 1- denoising with AmpliconNoise or Acacia, 2- selection of amplicons with the exact distal primer sequence and 3- detection of chimeras with UCHIME after Acacia denoising. (T) and (U) indicate the number of total and unique amplicons, respectively.**
(PDF)

Table S2 **Number of common and non-common radiolarian amplicons (without Acacia and AmpliconNoise denoising) between single-celled technical replicates (PCR and sequencing on the same DNA extract).** OTU reconstruction was performed with these amplicons at different identity levels.
(PDF)

Table S3 **Number of amplicons detected by the linkage method (See File S1).** The number of unique and redundant amplicons are indicated in the “Unique amplicon (Linkage)” and “Redundant amplicon (>1)” columns, respectively. The number of identical sequences between technical replicates or cells is given in the right part of the table (“Number of overlapped amplicons”).
(PDF)

File S1 (HTML)

Materials S1 (HTML)
