# Supplementary material for: Intracellular Diversity of the V4 and V9 Regions of the 18S rRNA in Marine Protists (Radiolarians) Assessed by High-Throughput Sequencing
Source: PLoS One. 2014 Aug 4;9(8):e104297. doi: 10.1371/journal.pone.0104297 (PMC4121268; doi:10.1371/journal.pone.0104297)

Relative size of the OTUs per sample at the 99% clustering level

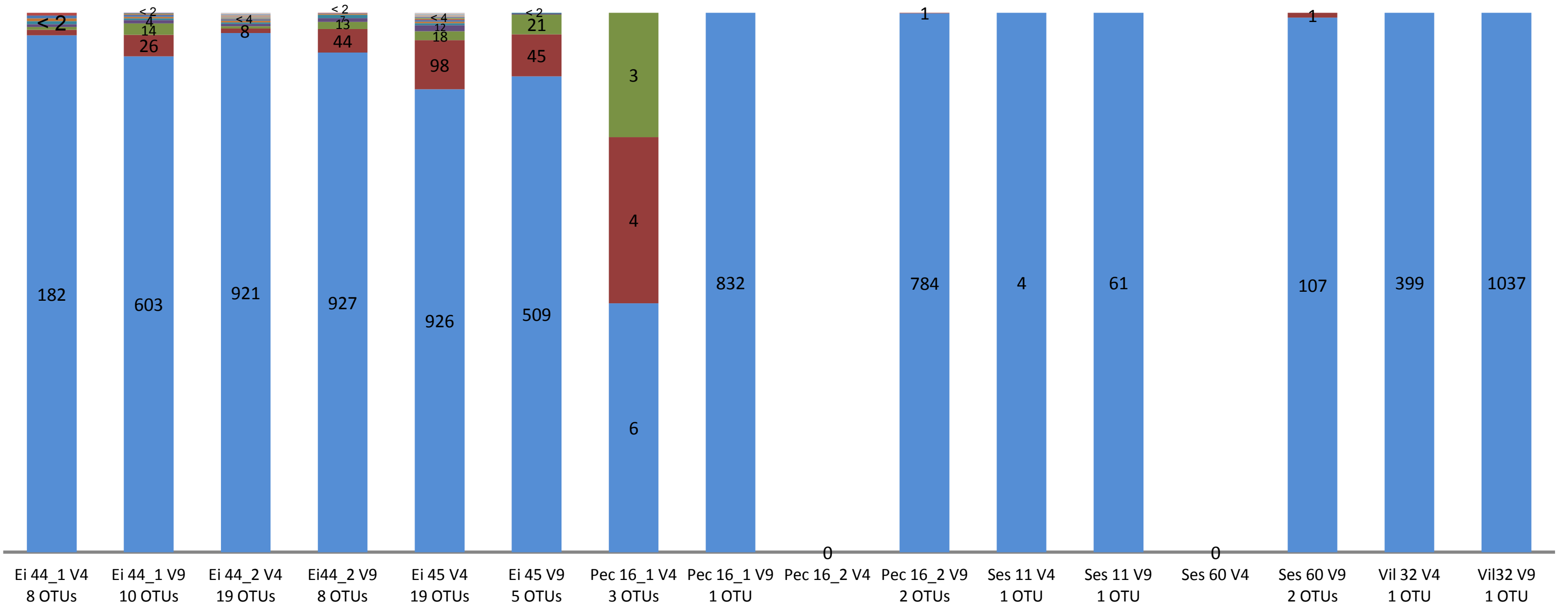

Relative size of the OTUs per sample at the 98% clustering level

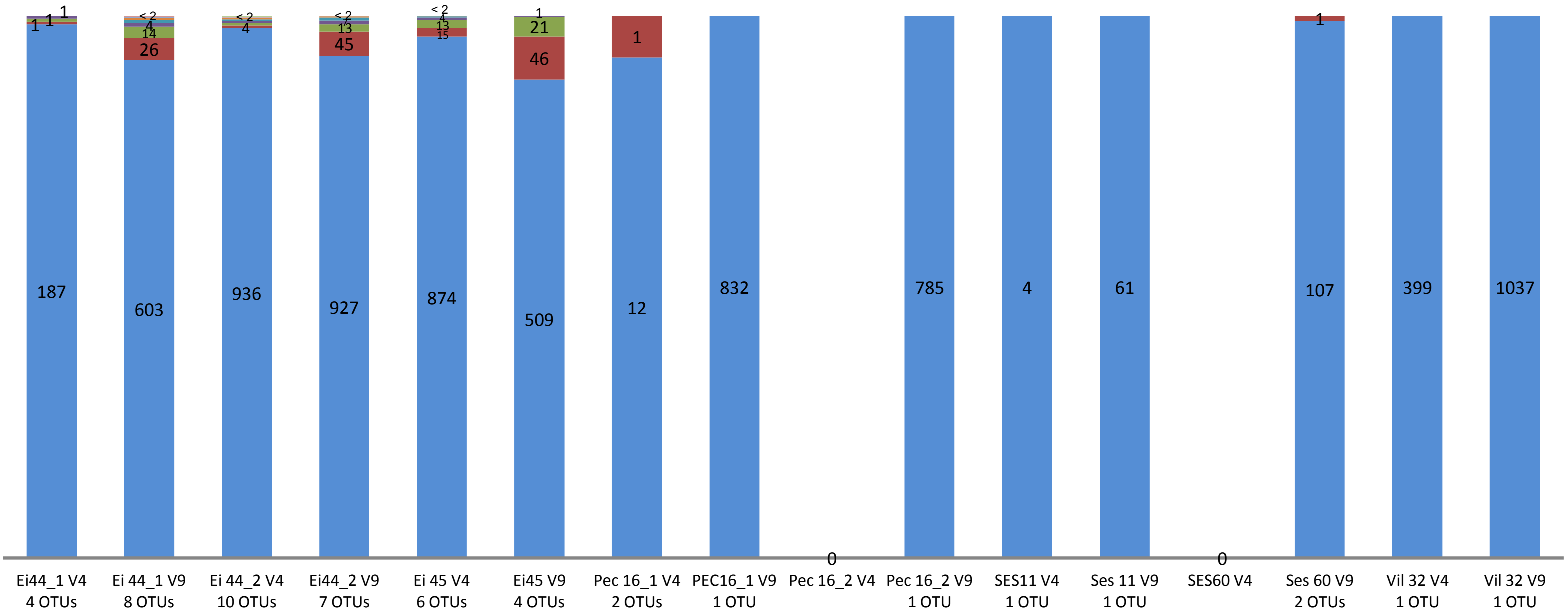

Supplement: Figure S1 — Number and size of the V4 and V9 OTUs found in different individual cells of Radiolaria, based on amplicons filtered with the denoising program Acacia. Each OTU is represented by a single color, and its number of amplicons is indicated in the bar. (PDF) [file pone.0104297.s001.pdf]
