# Supplementary figures and images for: Intracellular Diversity of the V4 and V9 Regions of the 18S rRNA in Marine Protists (Radiolarians) Assessed by High-Throughput Sequencing
Source: PLoS One. 2014 Aug 4;9(8):e104297. doi: 10.1371/journal.pone.0104297 (PMC4121268; doi:10.1371/journal.pone.0104297)

# Secondary structure of V4 reads of Ei 44\_1

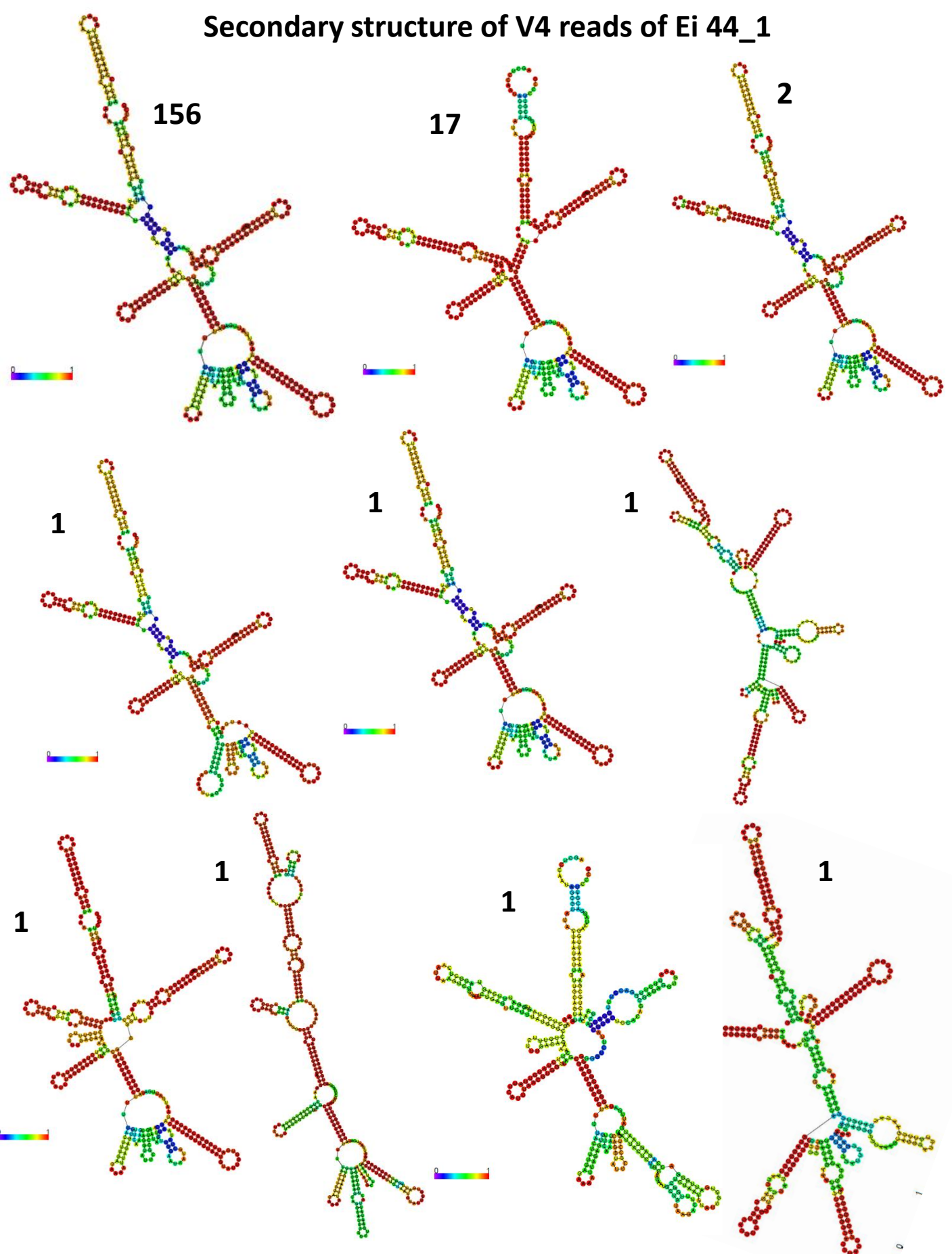

Supplement: Figure S2 — Predicted secondary structures of the V4 amplicons found in the sample Ei 44_1. The numbers indicate the abundance of the given amplicon. (PDF) [file pone.0104297.s002.pdf]
