# Supplementary material for: Intracellular Diversity of the V4 and V9 Regions of the 18S rRNA in Marine Protists (Radiolarians) Assessed by High-Throughput Sequencing
Source: PLoS One. 2014 Aug 4;9(8):e104297. doi: 10.1371/journal.pone.0104297 (PMC4121268; doi:10.1371/journal.pone.0104297)
Supplement: Figure S3 — V9 alignment comparison between reference sequences of all the clades of Acantharia (upper sequence consensus) and the common and non-common pyrosequenced amplicons obtained from technical replicates without Acacia denoising (Ei 44-1/Ei 44-2 and Pec 16-1 and Pec 16-2). Compared to non-common amplicons, common amplicons tend to have fewer substitutions in highly-conserved regions. (PDF) [file pone.0104297.s003.pdf]

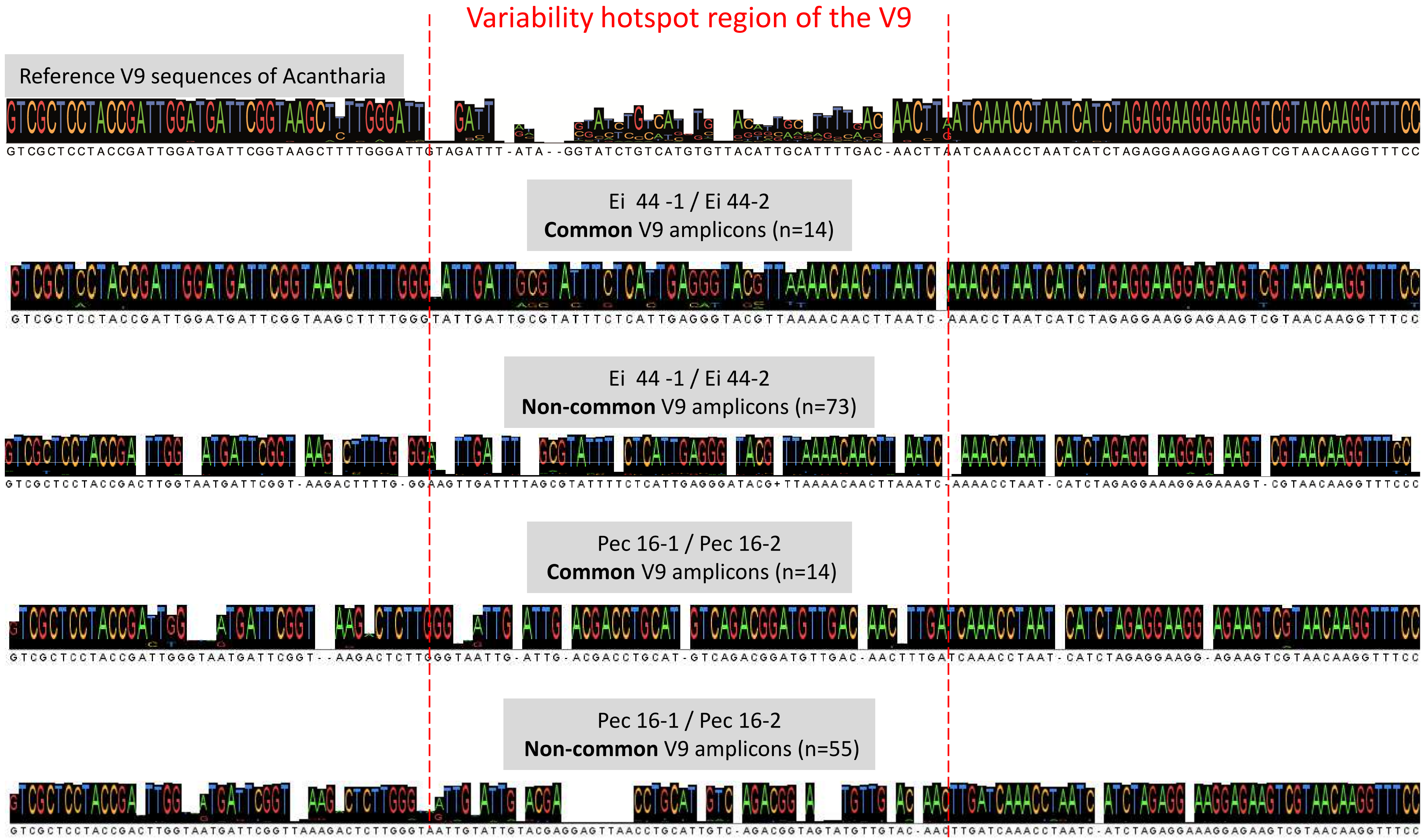
Variability hotspot region of the V9
Reference V9 sequences of Acantharia
Ei 44 -1 / Ei 44-2
Common V9 amplicons (n=14)
Ei 44 -1 / Ei 44-2
Non-common V9 amplicons (n=73)
Pec 16-1 / Pec 16-2
Common V9 amplicons (n=14)
Pec 16-1 / Pec 16-2
Non-common V9 amplicons (n=55)
