# Supplementary material for: Intracellular Diversity of the V4 and V9 Regions of the 18S rRNA in Marine Protists (Radiolarians) Assessed by High-Throughput Sequencing
Source: PLoS One. 2014 Aug 4;9(8):e104297. doi: 10.1371/journal.pone.0104297 (PMC4121268; doi:10.1371/journal.pone.0104297)
Supplement: Table S1 — Number of amplicons at the different consecutive filtering steps: 1- denoising with AmpliconNoise or Acacia, 2- selection of amplicons with the exact distal primer sequence and 3- detection of chimeras with UCHIME after Acacia denoising. (T) and (U) indicate the number of total and unique amplicons, respectively. (PDF) [file pone.0104297.s004.pdf]

Samples	Region	Raw amplicons	AmpliconNoise denoising				Acacia denoising				
			AmpliconNoise (T)	AmpliconNoise (U)	Amplicons with exact distal primer (T)	Amplicons with exact distal primers (U)	Acacia denoising	Amplicons with exact distal primer (T)	Amplicons with exact distal primers (U)	Chimera detection (T)	Chimera detection (U)
Ei 44_1	V4	6942	463	25	408	7	501	210	30	209	29
Ei 44_2	V4	7197	3059	83	2797	21	3156	1083	106	1073	96
Ei 45	V4	5142	2974	51	2809	19	2998	1287	134	1284	131
Pec 16_1	V4	2444	203	3	0	0	191	13	8	13	8
Pec 16_2	V4	1081	73	2	0	0	69	0	0	0	0
Ses 11	V4	5460	983	29	288	8	1100	193	29	193	29
Ses 60	V4	1088	78	8	54	3	151	16	6	16	6
Vil 32	V4	2906	1279	11	1247	2	1199	411	20	411	20
Ei 44_1	V9	2746	1167	72	1156	53	2168	1961	129	1960	128
Ei 44_2	V9	3899	2244	89	2225	64	3181	2747	127	2746	126
Ei 45	V9	1331	715	46	713	40	1097	974	61	974	61
Pec 16_1	V9	1538	1214	38	1207	25	1341	1211	49	1212	49
Pec 16_2	V9	1330	1052	26	1050	21	1127	1050	41	1050	41
Ses 11	V9	3913	2563	74	2555	56	2916	2617	129	2617	129
Ses 60	V9	2488	1751	49	1742	31	2075	1810	73	1810	73
Vil 32	V9	1793	1258	28	1254	21	1566	1453	31	1453	31
