# Supplementary material for: Intracellular Diversity of the V4 and V9 Regions of the 18S rRNA in Marine Protists (Radiolarians) Assessed by High-Throughput Sequencing
Source: PLoS One. 2014 Aug 4;9(8):e104297. doi: 10.1371/journal.pone.0104297 (PMC4121268; doi:10.1371/journal.pone.0104297)
Supplement: Table S2 — Number of common and non-common radiolarian amplicons (without Acacia and AmpliconNoise denoising) between single-celled technical replicates (PCR and sequencing on the same DNA extract). OTU reconstruction was performed with these amplicons at different identity levels. (PDF) [file pone.0104297.s005.pdf]

			**Non-filtered amplicons assigned to Radiolaria**		**Number of OTUs at different identity cut-off levels**																			
	**Replicates**	**Region**	**Total amplicons**	**Unique amplicons**	**99**	**98**	**97**	**96**	**95**	**94**	**93**	**92**	**91**	**90**	**89**	**88**	**87**	**86**	**85**	**84**	**83**	**82**	**81**	**80**
**Common reads**	Ei 44-1 / Ei 44-2	V4	1981	45	10	5	2	2	1	1	1	1	1	1	1	1	1	1	1	1	1	1	1	1
	Pec 16-1 / Pec 16-2	V4	1131	32	3	2	1	1	1	1	1	1	1	1	1	1	1	1	1	1	1	1	1	1
	Ei 44-1 / Ei 44-2	V9	1633	14	6	5	3	2	2	2	2	2	2	2	2	2	2	2	2	1	1	1	1	1
	Pec 16-1 / Pec 16-2	V9	1635	14	4	4	3	3	3	3	2	2	2	2	2	2	2	2	2	1	1	1	1	1
**Non-common reads**	Ei 44-1 / Ei 44-2	V4	1208	385	90	28	18	14	10	9	8	6	6	6	6	6	5	5	3	2	2	2	2	1
	Pec 16-1 / Pec 16-2	V4	193	176	37	9	5	2	1	1	1	1	1	1	1	1	1	1	1	1	1	1	1	1
	Ei 44-1 / Ei 44-2	V9	106	73	52	25	17	13	10	8	6	4	4	4	3	3	3	3	3	3	3	2	2	2
	Pec 16-1 / Pec 16-2	V9	71	55	41	15	10	7	6	5	5	4	4	4	4	4	4	3	3	3	3	3	3	3
