# Supplementary material for: Intracellular Diversity of the V4 and V9 Regions of the 18S rRNA in Marine Protists (Radiolarians) Assessed by High-Throughput Sequencing
Source: PLoS One. 2014 Aug 4;9(8):e104297. doi: 10.1371/journal.pone.0104297 (PMC4121268; doi:10.1371/journal.pone.0104297)
Supplement: Table S3 — Number of amplicons detected by the linkage method (See File S1). The number of unique and redundant amplicons are indicated in the “Unique amplicon (Linkage)” and “Redundant amplicon (>1)” columns, respectively. The number of identical sequences between technical replicates or cells is given in the right part of the table (“Number of overlapped amplicons”). (PDF) [file pone.0104297.s006.pdf]

						Number of overlapped amplicons															
						Ei 44_1	Ei 44_2	Ei 45	Pec 16_1	Pec 16_2	Ses 11	Ses 60	Vil 32	Ei 44_1	Ei 44_2	Ei 45	Pec 16_1	Pec 16_2	Ses 11	Ses 60	Vil 32
		Total amplicon	Unique amplicon	Unique amplicon	Redundant amplicon (>1)	V4	V4	V4	V4	V4	V4	V4	V4	V9	V9	V9	V9	V9	V9	V9	V9
Ei 44_1	V4	190	18 (15)	7	2	7	-	-	-	-	-	-	-	-	-	-	-	-	-	-	-
Ei 44_2	V4	957	52 (37)	21	13	3	21	-	-	-	-	-	-	-	-	-	-	-	-	-	-
Ei 45	V4	907	76 (52)	23	17	0 (2)*	0 (2)	23	-	-	-	-	-	-	-	-	-	-	-	-	-
Pec 16_1	V4	13	8 (5)	3	2	0	0	0	3	-	-	-	-	-	-	-	-	-	-	-	-
Pec 16_2	V4	0	0	0	0	0	0	0	0	0	-	-	-	-	-	-	-	-	-	-	-
Ses 11	V4	4	1	1	1	0	0	0	0	0	1	-	-	-	-	-	-	-	-	-	-
Ses 60	V4	0	0	0	0	0	0	0	0	0	0	0	-	-	-	-	-	-	-	-	-
Vil 32	V4	399	18 (15)	1	1	0	0	0	0	0	0	0	1	-	-	-	-	-	-	-	-
Ei 44_1	V9	656	18 (14)	7	7	-	-	-	-	-	-	-	-	7	3	1	0	0	0	0	0
Ei 44_2	V9	1001	11 (5)	7	4	-	-	-	-	-	-	-	-	-	4	1	0	0	0	0	0
Ei 45	V9	577	6 (3)	2	2	-	-	-	-	-	-	-	-	-	-	2	0	0	0	0	0
Pec 16_1	V9	832	4 (3)	1	1	-	-	-	-	-	-	-	-	-	-	-	1	1	0	0	0
Pec 16_2	V9	785	7 (6)	1	1	-	-	-	-	-	-	-	-	-	-	-	-	1	0	0	0
Ses 11	V9	61	1	1	1	-	-	-	-	-	-	-	-	-	-	-	-	-	1	0 (1)	0
Ses 60	V9	108	3 (2)	1	1	-	-	-	-	-	-	-	-	-	-	-	-	-	-	1	0
Vil 32	V9	1037	1	1	1	-	-	-	-	-	-	-	-	-	-	-	-	-	-	-	1

* Figures in parentheses showed the number of sequences with one substitution or indel.
