# Supplementary material for: Intracellular Diversity of the V4 and V9 Regions of the 18S rRNA in Marine Protists (Radiolarians) Assessed by High-Throughput Sequencing
Source: PLoS One. 2014 Aug 4;9(8):e104297. doi: 10.1371/journal.pone.0104297 (PMC4121268; doi:10.1371/journal.pone.0104297)
Supplement: File S1 — (HTML) [file pone.0104297.s007.html]

xml version="1.0" encoding="utf-8"?


Intracellular diversity of the V4 and V9 regions (18S rRNA) in eukaryotic cells assessed by 454 pyrosequencing


# Intracellular diversity of the V4 and V9 regions (18S rRNA) in eukaryotic cells assessed by 454 pyrosequencing

This document contains supplementary material for the paper
"Intracellular diversity of the V4 and V9 regions of the 18S rRNA in
marine protists (radiolarians) assessed by 454 pyrosequencing" by
Johan Decelle, Sarah Romac, Eriko Sasaki, Fabrice Not and Frédéric
Mahé.

## Table of Contents

- 1 Samples metadata
- 2 Wet lab
- 3 Bioinformatics 
  - 3.1 Disclaimer
  - 3.2 Analysis with denoising 
    - 3.2.1 Extract reads from SFF
    - 3.2.2 Acacia quality filtering
    - 3.2.3 AmpliconNoise quality filtering
  - 3.3 Analysis without denoising 
    - 3.3.1 Parse reads into samples
    - 3.3.2 Dereplicate
    - 3.3.3 Results per sample
    - 3.3.4 Taxonomic assignment of the reads
    - 3.3.5 Chimera checking with uchime
    - 3.3.6 Common amplicons
    - 3.3.7 Uncommon amplicons
    - 3.3.8 Triplicate: the case of Ei44 and Ei45
  - 3.4 Linkage Method
- 4 Final fasta files

## 1 Samples metadata

Individual cells were collected by Johan Decelle at the following
locations:

| sample | lattitude | longitude | Ocean |
| --- | --- | --- | --- |
| Vil32 | 43°40'55.20"N | 7°18'44.76"E | Mediterranean sea |
| Pec\_16 | 43°40'55.20"N | 7°18'44.76"E | Mediterranean sea |
| Ei44 | 29°30'18.15"N | 34°57'25.01"E | Red sea |
| Ei45 | 29°30'18.15"N | 34°57'25.01"E | Red sea |
| SES11 | 26°37'20 N | 127°52’15 E | Western North Pacific Ocean |
| SES60 | 26°37'20 N | 127°52’15 E | Western North Pacific Ocean |

## 2 Wet lab

DNA was extracted from the cell according to Decelle et al.
(2012a). Amplifications were conducted with Phusion® High-Fidelity DNA
Polymerase (Finnzymes). The PCR mixture (25 µL final volume) contained
5 ng of template with 0.35 µM final concentration of each primer, 3%
of DMSO and 2X of GC buffer Phusion Master Mix (Finnzymes). The V4
primer sequences were 5'-CCAGCASCYGCGGTAATTCC-3' and
5'-ACTTTCGTTCTTGATYRA-3'; and the V9 primer sequences were
5'-TTGTACACACCGCCC-3' and 5'-CCTTCYGCAGGTTCACCTAC-3'.

Amplifications of the V4 region were done following the PCR program:
initial denaturation step at 98°C for 30 sec, followed by 10 cycles of
10 sec at 98°C, 30 sec at 53°C, 30 sec at 72°C, followed by 15 cycles
of 10 sec at 98°C, 30 sec at 48°C, 30 sec at 72°C and final elongation
step at 72°C for 10 minutes.

Amplifications of the V9 region were done following the PCR program:
initial denaturation step at 98°C for 30 sec, followed by 25 cycles of
10 sec at 98°C, 30 sec at 57°C, 30 sec at 72°C, followed by 15 cycles
of 10 sec at 98°C, 30 sec at 48°C, 30 sec at 72°C and final elongation
step at 72°C for 10 minutes.

Each sample was amplified in triplicate to get enough amounts of
amplicons. Products of the reactions were run on a 1.5% agarose gel to
check for successful amplification products of the expected
length. Amplicons were tagged with 8-bp long unique identifiers, then
pooled and purified using the NucleoSpin® Extract II kit
(Macherey-Nagel, Hoerdt, France). To obtain a similar number of reads
for each sample, purified amplicons for each condition were quantified
with the Quant-iT™ PicoGreen ® dsDNA kit (Invitrogen) and then mixed
in equal concentrations.

Pools of amplicons where finally sent to the CEA Genoscope in Evry
(France). Emulsion PCR and sequencing were performed using a GS FLX
emPCR Genomic Lib-L kit according to the manufacturer's protocol
(Genome Sequencer FLX Titanium, 454 Life Sciences from Roche,
Brandford, CT, USA).

The raw sequences have been deposited on the Short Read Archive, under
the accession number PRJEB4199.

## 3 Bioinformatics

### 3.1 Disclaimer

The purpose of this document is too provide the reader with details on
the bioinformatics methods we used to prepare the paper "Metabarcode
diversity from single eukaryotic cells". The code snippets and shell
commands presented here were executed on a Debian GNU/Linux 6, and
might have to be adapted to your particular system. Use them
carrefully.

### 3.2 Analysis with denoising

#### 3.2.1 Extract reads from SFF

Once the SFF file has been downloaded, extract the sequences (fasta
format) and the quality values.

```
sffinfo -s ALI_AFBOTS_HQC2HZF02.sff > ALI_AFBOTS_HQC2HZF02.fas
sffinfo -q ALI_AFBOTS_HQC2HZF02.sff > ALI_AFBOTS_HQC2HZF02.qual
```

We count 182,194 raw reads:

```
grep -c "^>" ../data/2.TCA.ALI_AFBOTS_HQC2HZF02.fna
```

The read lengths are distributed as such:

```
grep -E -o "length=[1-9]+" ../data/2.TCA.ALI_AFBOTS_HQC2HZF02.fna | sed -e 's/length=//' | sort -n > ../results/lengths.data
```

#### 3.2.2 Acacia quality filtering

- Run Acacia   

  Starting from the raw data, we used Acacia v1.52-b0 (Bragg et al., 2012) with default parameters:

  ```
  java -jar acacia-1.52.b0.jar -c Single_Cell_Radiolarians.config
  ```

  With this configuration file:

  ```
  CONFIG="ANY_DIFF_SIGNIFICANT_FOR_TWO_SEQS=TRUE
  AVG_QUALITY_CUTOFF=30
  ERROR_MODEL=Balzer
  FASTA=TRUE
  FASTA_LOCATION=2.TCA.ALI_AFBOTS_HQC2HZF02.fna
  FASTQ=FALSE
  FILTER_N_BEFORE_POS=350
  FLOW_CYCLE_STRING=TACG
  FLOW_KEY=TCAG
  MAXIMUM_MANHATTAN_DISTANCE=13
  MAX_RECURSE_DEPTH=2
  MAX_STD_DEV_LENGTH=2
  MIN_FLOW_TRUNCATION=150
  MIN_READ_REP_BEFORE_TRUNCATION=0.0
  OUTPUT_DIR=./results/
  OUTPUT_PREFIX=Single_Cell_Radiolarians
  QUAL_LOCATION=2.TCA.ALI_AFBOTS_HQC2HZF02.qual
  REPRESENTATIVE_SEQUENCE=Mode
  SIGNIFICANCE_LEVEL=-9
  SPLIT_ON_MID=TRUE
  TRIM_TO_LENGTH=
  TRUNCATE_READ_TO_FLOW=
  MID_OPTION=LOAD_MIDS
  MID_FILE=Single_Cell_Radiolarians.selectedMIDS"

  echo "${CONFIG}" > Single_Cell_Radiolarians.config
  ```

  And the following MID FILE:

  ```
  # MID_NAME, MID_TAG,PRIMER_SEQUENCE
  MIDs="EI44_1_V4 CAATAGG CCAGCASCYGCGGTAATTCC
  Ei44_2_V4 AACAACAA CCAGCASCYGCGGTAATTCC
  Ei45_V4 AGCATGCG CCAGCASCYGCGGTAATTCC
  PEC16_1_V4 CTTCTTCA CCAGCASCYGCGGTAATTCC
  PEC16_2_V4 AACAATGG CCAGCASCYGCGGTAATTCC
  Vil32_V4 GAGTACTA CCAGCASCYGCGGTAATTCC
  SES11_V4 TATCACAT CCAGCASCYGCGGTAATTCC
  SES60_V4 CCAGTCAG CCAGCASCYGCGGTAATTCC
  PAC_16_V4 CTATAAGT CCAGCASCYGCGGTAATTCC
  PAC_19_V4 ATGTATAA CCAGCASCYGCGGTAATTCC
  EI44_1_V9 ACGGAACC TTGTACACACCGCCC
  Ei44_2_V9 CGGAAGAC TTGTACACACCGCCC
  Ei45_V9 AATAGTCC TTGTACACACCGCCC
  PEC16_1_V9 AGGTATGG TTGTACACACCGCCC
  PEC16_2_V9 CCAACCTG TTGTACACACCGCCC
  Vil32_V9 TTCTAGTA TTGTACACACCGCCC
  SES11_V9 GAGAGCTG TTGTACACACCGCCC
  SES60_V9 TAGTATTC TTGTACACACCGCCC
  PAC_16_V9 AATATCGC TTGTACACACCGCCC
  PAC_19_V9 TCAGGTTC TTGTACACACCGCCC"

  echo "${MIDs}" | tr " " "\t"  > Single_Cell_Radiolarians.selectedMIDS
  ```
- Discard reads without the reverse primer   

  Simply discard amplicons that do not contain the distal primer is a
  very efficient filtering method. It guarantees that amplicons are full
  length and it reduces the number of unique amplicons.

  ```
  # on my computer  
  # Trim primer R and dereplicate (V9)
  for f in *_V9.seqOut ; do
      grep "GTAGGTGAACCTGC[AG]GAAGG" "${f}" |
      sed -e 's/GTAGGTGAACCTGC[AG]GAAGG.*//' | sort -d | uniq -c |
      while read abundance sequence ; do
          hash=$(echo ${sequence} | sha1sum)
          hash=${hash:0:40}
          printf ">%s_%d_%s\n" "${hash}" "${abundance}" "${sequence}"
      done | sort -t "_" -k2,2nr | sed -e 's/\_/\n/2' > ${f/.seqOut/_trimmed_dereplicated.fas}
  done
  # Trim primer R and dereplicate (V4)
  for f in *_V4.seqOut ; do
      grep "T[CT][AG]ATCAAGAACGAAAGT" "${f}" |
      sed -e 's/T[CT][AG]ATCAAGAACGAAAGT.*//' | sort -d | uniq -c |
      while read abundance sequence ; do
          hash=$(sha1sum <<< ${sequence})
          hash=${hash:0:40}
          printf ">%s_%d_%s\n" "${hash}" "${abundance}" "${sequence}"
      done | sort -t "_" -k2,2nr | sed -e 's/\_/\n/2' > ${f/.seqOut/_trimmed_dereplicated.fas}
  done
  ```
- Chimera checking with uchime   

  We use the module uchime in the package usearch to find and eliminate
  chimeras. Usearch documentation advise to modify default parameters
  for SSU rRNA amplicon-based studies: "For example, in a 16S experiment
  using 200nt reads, clusters of radius ~3% might be used in an attempt
  to identify species. It would then be important to identify chimeras
  with divergences as low as ~2%, which could have a few as four diffs
  with their closest parents. In such cases, the small amount of
  evidence available should increase the uncertainty of the
  classification."

  ```
  mkdir -p uchime
  # copy fasta files and modify the fasta header so usearch gets copy
  # numbers (convert >readid_N to >readid;size=N)
  for f in *.fas ; do
      sed -e 's/\_/;size=/' ${f} > uchime/${f}
  done
  # uchime (-chimeras and -nochimeras options do not work)
  cd uchime/
  TEMP=$(mktemp)
  USEARCH="usearch7.0.1001_i86linux32"
  for f in *_dereplicated.fas ; do
      if [[ -s "${f}" ]] ; then
          "${USEARCH}" -uchime_denovo "${f}" -uchimeout "${f/.fas/.uchime}"
          # List chimeras
          awk '{if ($NF == "Y") print $2}' "${f/.fas/.uchime}" > "${TEMP}"
          grep -A 1 -F -f "${TEMP}" "${f}" | sed -e '/^--$/d' -e 's/;size=/_/' > "${f/.fas/_uchime_rejected.fas}"
          # List non-chimeras
          awk '{if ($NF == "N") print $2}' "${f/.fas/.uchime}" > "${TEMP}"
          grep -A 1 -F -f "${TEMP}" "${f}" | sed -e '/^--$/d' -e 's/;size=/_/' > "${f/.fas/_uchime_validated.fas}"
      else
          # Deal with empty fasta files
          cp "${f}" "${f/.fas/_uchime_validated.fas}"
      fi
  done
  rm -f "${TEMP}"
  ```
- Amplicons taxonomic assignment   

  Merge all reads into one file (one V4 and one V9). The taxonomic
  assignment method we use is based on exact pairwise global alignment
  (Needleman-Wunsch). It relies on ggsearch, a tool from the FASTA36
  package. The references we use are distributed on the PR2 website. The
  amplicons are assigned to the closest reference sequence, based on the
  percentage of identity. In case of equidistance with two or more
  references, the amplicon is assigned to the last common ancestor. We
  used a more complex script to distribute the computation load on
  several computers. The code being very specific to our IT system, we
  prefer not to distribute it, but present this simplified code:

  ```
  cat 7R0227_reduced.fas 8R00* > all_V4.fas
  cat 8R07* > all_V9.fas
  mkdir V9 V4
  mv all_V4.fas V4/
  mv all_V9.fas V9/
  cd V9/
  ggsearch36 -q -n -3 -T 1 -z -1 -m 10 all_V9.fas V9_reference.fas |
  grep -E "^>{2,3}[A-Za-Z0-9]|^; gnw_ident:" > tmp
  python mass_parse_ggsearch.py tmp | sort -k2nr > all_V9.results
  rm tmp
  cd ..
  cd V4/
  ggsearch36 -q -n -3 -T 1 -z -1 -m 10 all_V4.fas V4_reference.fas |
  grep -E "^>{2,3}[A-Za-Z0-9]|^; gnw_ident:" > tmp
  python mass_parse_ggsearch.py tmp | sort -k2nr > all_V4.results
  rm tmp
  cd ..
  ```

  The python script mass\_parse\_ggsearch.py is reproduced below:

  ```
  #!/usr/bin/env python
  # -*- coding: utf-8 -*-
  """
      Parse the results produced by the m 10 output option of
      ggsearch36.
  """

  from __future__ import print_function

  __author__ = "Frédéric Mahé <mahe@rhrk.uni-kl.de>"
  __date__ = "2012/01/20"
  __version__ = "$Revision: 1.0"

  import os
  import sys
  from decimal import *

  #**********************************************************************#
  #                                                                      #
  #                            Functions                                 #
  #                                                                      #
  #**********************************************************************#

  def get_taxonomic_consensus(best_hits):
      """
      Calculate a taxonomic consensus.
      In Laure's database, taxonomic levels: 0 = Eukaryota, 6 = genus, 7 = species.
      In Silva, we have only four levels. Six for mitochondria.
      """
      separator="|"
      taxa = [taxon[1].split(separator) for taxon in best_hits]
      taxonomic_consensus = list()
      lengths = set([len(taxon) for taxon in taxa])
      # Deal with unequal taxonomic descriptions lengths
      if len(lengths) == 1:
          max_fields = list(lengths)[0]
      else:
          max_fields = max(lengths)
          new_taxa = list()
          for taxon in taxa:
              while len(taxon) < max_fields:
                  taxon.append("*")
              new_taxa.append(taxon)
          taxa = new_taxa
      # Compute consensus
      for i in xrange(0, max_fields, 1):
          level = list(set([taxon[i] for taxon in taxa]))
          if len(level) == 1:
              taxonomic_consensus.append("".join(level))
          else:
              taxonomic_consensus.append("*")
      taxonomic_consensus = separator.join(taxonomic_consensus)

      return taxonomic_consensus

  #**********************************************************************#
  #                                                                      #
  #                              Body                                    #
  #                                                                      #
  #**********************************************************************#

  if __name__ == '__main__':
      
      # Parse command line options.
      input_file = sys.argv[1]

      data = dict()

      with open(input_file, "rU") as input_file:
          # Store all hit data in a dictionary structure
          for line in input_file:
              line = line.strip()
              if line.startswith(">>>"):
                  read_id, abundance = line.split(",")[0].lstrip(">").split("_")
                  data[read_id] = [abundance, []] 
              elif line.startswith(">>"):
                  ref_id, taxonomy = line.lstrip(">").split(" ", 1)
              elif line.startswith(";"):
                  # Ugly trick to get the identity to not be treated as a float.
                  identity = Decimal(line.split(":")[1].strip(" ")) * 100
                  identity = Decimal(identity).quantize(Decimal('.1'))
                  data[read_id][1] += [[identity, ref_id, taxonomy]]
          # Search best hits for each query 
          for key in data:
              abundance = data[key][0]
              identities = [triplet[0] for triplet in [triplets for triplets in data[key][1]]]
              top= max(identities)
              best_hits = list()
              test = list()
              # Search for max identities
              for triplet in data[key][1]:
                  identity = triplet[0]
                  ref_id = triplet[1]
                  taxonomy = triplet[2]
                  if identity == top:
                      best_hits.append((ref_id, taxonomy))
              if len(best_hits) == 1:
                  taxonomy = best_hits[0][1]
                  ref_ids = best_hits[0][0]
              elif len(best_hits) > 1:
                  taxonomy = get_taxonomic_consensus(best_hits)
                  ref_ids = ",".join([couple[0] for couple in best_hits])
              # Deal with the "no hit" case
              elif len(best_hits) == 0:
                  top = "NA"
                  taxonomy = "NA"
                  ref_ids = "NA"
              print(key, abundance, top, taxonomy, ref_ids, sep="\t")

  sys.exit(0)
  ```
- Filtering based on taxonomic assignment   

  From the list of amplicons that passed all filters, keep only those
  assigned to Radiolaria.

  ```
  cd ./uchime/
  # List Rhizaria hits in each sample
  for REGION in V4 V9 ; do
      TAXO_RESULTS="../all_${REGION}_trimmed_dereplicated_uchime_validated.results"
      for f in *_${REGION}_trimmed_dereplicated_uchime_validated.fas ; do
          SAMPLE=${f/Single_Cell_Radiolarians_/}
          SAMPLE=${SAMPLE/_V*_trimmed_dereplicated_uchime_validated.fas/}
          echo -en "${SAMPLE}\t${REGION}\t"
          grep "^>" "${f}" | tr -d ">" | tr "_" " " |
          while read id ab ; do
              taxo=$(grep -m 1 $id "${TAXO_RESULTS}" | cut -f 3-4)
              echo -e "${id}\t${ab}\t${taxo}"
          done | grep "Radiolaria" | awk 'BEGIN {OFS="\t"; sum=0} {sum+=$2} END {print sum, NR}'
      done
  done
  ```

  Amplicons assigned to Radiolaria (or not).

  |  |  | Radiolaria | Radiolaria | Non-Radiolaria |  |
  | --- | --- | --- | --- | --- | --- |
  | Sample | region | reads | unique | reads | unique |
  | Ei44\_1 | V4 | 190 | 18 | 19 | 11 |
  | Ei44\_2 | V4 | 957 | 52 | 116 | 44 |
  | Ei45 | V4 | 909 | 78 | 375 | 53 |
  | PEC16\_1 | V4 | 13 | 8 | 0 | 0 |
  | PEC16\_2 | V4 | 0 | 0 | 0 | 0 |
  | SES11 | V4 | 4 | 1 | 189 | 28 |
  | SES60 | V4 | 0 | 0 | 16 | 6 |
  | Vil32 | V4 | 399 | 18 | 12 | 2 |
  | Ei44\_1 | V9 | 656 | 18 | 1304 | 110 |
  | Ei44\_2 | V9 | 1001 | 11 | 1745 | 115 |
  | Ei45 | V9 | 577 | 6 | 397 | 55 |
  | PEC16\_1 | V9 | 842 | 8 | 370 | 41 |
  | PEC16\_2 | V9 | 785 | 7 | 265 | 34 |
  | SES11 | V9 | 61 | 1 | 2556 | 128 |
  | SES60 | V9 | 114 | 4 | 1696 | 69 |
  | Vil32 | V9 | 1040 | 3 | 413 | 28 |

  The final filtered samples (fasta format) has been joined to that file
  (see section "Final fasta files").
- Summary of filtering results   

  | Taxa | region | Acacia | distal (T) | distal (U) | uchime (T) | uchime (U) | Radiolaria (T) | Radiolaria (U) | Other (T) | Other (U) |
  | --- | --- | --- | --- | --- | --- | --- | --- | --- | --- | --- |
  | Ei44\_1 | V4 | 501 | 210 | 30 | 209 | 29 | 190 | 18 | 19 | 11 |
  | Ei44\_2 | V4 | 3156 | 1083 | 106 | 1073 | 96 | 957 | 52 | 116 | 44 |
  | Ei45 | V4 | 2998 | 1287 | 134 | 1284 | 131 | 909 | 78 | 375 | 53 |
  | PEC16\_1 | V4 | 191 | 13 | 8 | 13 | 8 | 13 | 8 | 0 | 0 |
  | PEC16\_2 | V4 | 69 | 0 | 0 | 0 | 0 | 0 | 0 | 0 | 0 |
  | SES11 | V4 | 1100 | 193 | 29 | 193 | 29 | 4 | 1 | 189 | 28 |
  | SES60 | V4 | 151 | 16 | 6 | 16 | 6 | 0 | 0 | 16 | 6 |
  | Vil32 | V4 | 1199 | 411 | 20 | 411 | 20 | 399 | 18 | 12 | 2 |
  | Ei44\_1 | V9 | 2168 | 1961 | 129 | 1960 | 128 | 656 | 18 | 1304 | 110 |
  | Ei44\_2 | V9 | 3181 | 2747 | 127 | 2746 | 126 | 1001 | 11 | 1745 | 115 |
  | Ei45 | V9 | 1097 | 974 | 61 | 974 | 61 | 577 | 6 | 397 | 55 |
  | PEC16\_1 | V9 | 1341 | 1211 | 49 | 1212 | 49 | 842 | 8 | 370 | 41 |
  | PEC16\_2 | V9 | 1127 | 1050 | 41 | 1050 | 41 | 785 | 7 | 265 | 34 |
  | SES11 | V9 | 2916 | 2617 | 129 | 2617 | 129 | 61 | 1 | 2556 | 128 |
  | SES60 | V9 | 2075 | 1810 | 73 | 1810 | 73 | 114 | 4 | 1696 | 69 |
  | Vil32 | V9 | 1566 | 1453 | 31 | 1453 | 31 | 1040 | 3 | 413 | 28 |

  legend: T = Total number of reads; U = unique reads
- Clustering with Usearch   

  To get a precise idea of the content of each sample, we used uclust
  (usearch7.0.1001\_i86linux32) to compute the number of OTUs for all
  clustering levels from 80% to 99%.

  ```
  cd ./uchime/
  # Extract Radiolaria and clusterize 
  USEARCH="usearch7.0.1001_i86linux32"
  for REGION in V4 V9 ; do
      TAXO_RESULTS="../all_${REGION}_trimmed_dereplicated_uchime_validated.results"
      TMP_RADIOLARIA=$(mktemp)
      grep "Radiolaria" "${TAXO_RESULTS}" | cut -f 1 > "${TMP_RADIOLARIA}"
      for f in *_${REGION}_trimmed_dereplicated_uchime_validated.fas ; do
          grep -A 1 -F -f "${TMP_RADIOLARIA}" "${f}" | sed -e '/^--$/d' > "${f/.fas/_rhizaria.fas}"
          for THRESHOLD in {80..99} ; do
              OUTPUT_CLUSTERS="${f/.fas/_rhizaria.uclust_}${THRESHOLD}"
              TMP_USEARCH=$(mktemp)
              "${USEARCH}" -cluster_smallmem -usersort "${f/.fas/_rhizaria.fas}" -id 0.${THRESHOLD} -uc "${TMP_USEARCH}"
              grep "^S" "${TMP_USEARCH}" | 
              while read a b c d e f g h i j ; do
                  hits=$(grep "^H.*${i}$" "${TMP_USEARCH}" | cut -f 9 | tr "\n" " " | sed -e 's/\ $//')
                  echo "${i} ${hits}"
              done > "${OUTPUT_CLUSTERS}"
              rm "${TMP_USEARCH}"
          done
      done
      rm "${TMP_RADIOLARIA}"
  done

  # Parsing
  for SAMPLE in Ei44_1 Ei44_2 Ei45 PEC16_1 PEC16_2 SES11 SES60 Vil32 ; do
      for REGION in V4 V9 ; do
          CLUSTER_COUNTS=$(wc -l Single_Cell_Radiolarians_${SAMPLE}_${REGION}_trimmed_dereplicated_uchime_validated_rhizaria.uclust_* | grep "uclust" | awk 'BEGIN {ORS=","} {print $1} END {print "\n"}' | sed -e 's/,$//')
          echo "${SAMPLE},${REGION},${CLUSTER_COUNTS}"
      done
  done | sort -t "," -k2,2d
  ```

  Number of OTUs at different clustering thresholds ("Uniques" column
  represent the number of OTUs at 100%).

  | Sample | Region | Reads | Uniques | 80 | 81 | 82 | 83 | 84 | 85 | 86 | 87 | 88 | 89 | 90 | 91 | 92 | 93 | 94 | 95 | 96 | 97 | 98 | 99 |
  | --- | --- | --- | --- | --- | --- | --- | --- | --- | --- | --- | --- | --- | --- | --- | --- | --- | --- | --- | --- | --- | --- | --- | --- |
  | Ei44\_1 | V4 | 190 | 18 | 1 | 1 | 1 | 1 | 1 | 1 | 1 | 1 | 1 | 1 | 1 | 1 | 1 | 1 | 2 | 2 | 3 | 3 | 4 | 8 |
  | Ei44\_2 | V4 | 957 | 52 | 1 | 1 | 1 | 1 | 1 | 1 | 1 | 1 | 1 | 1 | 1 | 1 | 1 | 1 | 1 | 2 | 2 | 5 | 10 | 19 |
  | Ei45 | V4 | 909 | 78 | 2 | 2 | 2 | 2 | 2 | 2 | 2 | 2 | 2 | 2 | 2 | 2 | 2 | 2 | 2 | 2 | 3 | 3 | 6 | 21 |
  | PEC16\_1 | V4 | 13 | 8 | 1 | 1 | 1 | 1 | 1 | 1 | 1 | 1 | 1 | 1 | 1 | 1 | 1 | 1 | 1 | 1 | 1 | 1 | 2 | 3 |
  | PEC16\_2 | V4 | 0 | 0 | 0 | 0 | 0 | 0 | 0 | 0 | 0 | 0 | 0 | 0 | 0 | 0 | 0 | 0 | 0 | 0 | 0 | 0 | 0 | 0 |
  | SES11 | V4 | 4 | 1 | 1 | 1 | 1 | 1 | 1 | 1 | 1 | 1 | 1 | 1 | 1 | 1 | 1 | 1 | 1 | 1 | 1 | 1 | 1 | 1 |
  | SES60 | V4 | 0 | 0 | 0 | 0 | 0 | 0 | 0 | 0 | 0 | 0 | 0 | 0 | 0 | 0 | 0 | 0 | 0 | 0 | 0 | 0 | 0 | 0 |
  | Vil32 | V4 | 399 | 18 | 1 | 1 | 1 | 1 | 1 | 1 | 1 | 1 | 1 | 1 | 1 | 1 | 1 | 1 | 1 | 1 | 1 | 1 | 1 | 1 |
  | Ei44\_1 | V9 | 656 | 18 | 1 | 1 | 1 | 1 | 1 | 1 | 1 | 1 | 1 | 1 | 2 | 2 | 2 | 2 | 3 | 3 | 3 | 5 | 8 | 10 |
  | Ei44\_2 | V9 | 1001 | 11 | 2 | 2 | 2 | 2 | 2 | 2 | 2 | 2 | 2 | 2 | 3 | 3 | 3 | 3 | 3 | 3 | 3 | 4 | 7 | 8 |
  | Ei45 | V9 | 577 | 6 | 1 | 1 | 1 | 1 | 1 | 1 | 1 | 1 | 1 | 1 | 2 | 2 | 2 | 2 | 3 | 3 | 3 | 3 | 4 | 5 |
  | PEC16\_1 | V9 | 842 | 8 | 3 | 3 | 3 | 3 | 3 | 3 | 3 | 3 | 3 | 3 | 3 | 3 | 3 | 3 | 3 | 3 | 3 | 3 | 3 | 4 |
  | PEC16\_2 | V9 | 785 | 7 | 1 | 1 | 1 | 1 | 1 | 1 | 1 | 1 | 1 | 1 | 1 | 1 | 1 | 1 | 1 | 1 | 1 | 1 | 1 | 2 |
  | SES11 | V9 | 61 | 1 | 1 | 1 | 1 | 1 | 1 | 1 | 1 | 1 | 1 | 1 | 1 | 1 | 1 | 1 | 1 | 1 | 1 | 1 | 1 | 1 |
  | SES60 | V9 | 114 | 4 | 2 | 2 | 2 | 2 | 2 | 2 | 2 | 2 | 2 | 2 | 2 | 2 | 2 | 2 | 2 | 2 | 2 | 2 | 3 | 3 |
  | Vil32 | V9 | 1040 | 3 | 2 | 2 | 2 | 2 | 2 | 2 | 2 | 2 | 3 | 3 | 3 | 3 | 3 | 3 | 3 | 3 | 3 | 3 | 3 | 3 |
- Sample intersections and sample pooling   

  The filenames are very long, I create shorter symbolic links.

  ```
  cd ./uchime/

  # Link fasta files
  for f in Single_Cell_Radiolarians_*_trimmed_dereplicated_uchime_validated.fas ; do
      sample=${f/Single_Cell_Radiolarians_/}
      sample=${sample/_trimmed/}
      sample=${sample/_uchime_validated/}
      ln -s $f $sample
  done

  # Link taxonomic assignments
  ln -s ../all_V4_trimmed_dereplicated_uchime_validated.results all_V4.results
  !!:gs/V4/V9/
  ```

  - Sample intersection   

    ```
    ### Intersection of samples, taxonomy based filtering and clustering

    cd ./uchime/

    # List all replicate pairs
    replicates="Ei44_1_V4 Ei44_2_V4
    Ei44_1_V4 Ei45_V4
    Ei44_2_V4 Ei45_V4
    PEC16_1_V4 PEC16_2_V4
    SES11_V4 SES60_V4
    Ei44_1_V9 Ei44_2_V9
    Ei44_1_V9 Ei45_V9
    Ei44_2_V9 Ei45_V9
    PEC16_1_V9 PEC16_2_V9
    SES11_V9 SES60_V9"

    rm tmp*

    # Extract non-common reads, remove non-radiolarians, clusterize and
    # make a summary
    while read replicates ; do
        sampleA=${replicates% *}
        sampleB=${replicates#* }
        region=${replicates##*_}
        
        intersection="${sampleA}_vs_${sampleB}.fas"
        intersection_radiolaria="${sampleA}_vs_${sampleB}_radiolaria.fas"
        all_samples="${sampleA}_dereplicated.fas ${sampleB}_dereplicated.fas"

        # Extract common reads
        COMMON_READS=$(mktemp)
        RADIOLARIA_READS=$(mktemp)
        grep -h "^>" ${all_samples} | tr -d ">" | cut -d '_' -f 1 | sort -d | uniq -d > "${COMMON_READS}"
        grep -A 1 -F -f "${COMMON_READS}" ${sampleA}_dereplicated.fas | sed -e '/^--$/d' > "${intersection}"

        # Limit to Radiolaria only
        grep -F -f "${COMMON_READS}" "all_${region}.results" | sed -e '/^--$/d' | grep "Radiolaria" | cut -f 1 | sort -du > "${RADIOLARIA_READS}"
        grep -A 1 -F -f "${RADIOLARIA_READS}" ${sampleA}_dereplicated.fas | sed -e '/^--$/d' > "${intersection_radiolaria}"

        # Clusterize
        USEARCH="usearch7.0.1001_i86linux32"
        TMP_USEARCH=$(mktemp)
        for THRESHOLD in {80..99} ; do
            OUTPUT_CLUSTERS="${intersection_radiolaria/.fas/.uclust_}${THRESHOLD}"
            "${USEARCH}" -cluster_smallmem -usersort "${intersection_radiolaria}" -id 0.${THRESHOLD} -uc "${TMP_USEARCH}" &> /dev/null
            grep "^S" "${TMP_USEARCH}" | 
            while read a b c d e f g h i j ; do
                hits=$(grep "^H.*${i}$" "${TMP_USEARCH}" | cut -f 9 | tr "\n" " " | sed -e 's/\ $//')
                echo "${i} ${hits}"
            done > "${OUTPUT_CLUSTERS}"
        done
        rm "${TMP_USEARCH}"
      
        # Parse clustering results (all levels, and 98%)
        total=$(grep "^>" "${intersection}" |
            while read l ; do
                read_id=${l%_*}
                grep -h "$read_id" ${all_samples}
            done | awk 'BEGIN {FS="_"} {sum+=$NF} END {print sum}')
        uniques=$(grep -c "^>" "${intersection}")
        radiolaria_uniques=$(wc -l < "${RADIOLARIA_READS}")
        radiolaria_total=$(grep -h -F -f "${RADIOLARIA_READS}" ${all_samples} | awk 'BEGIN {FS="_"} {sum += $2} END {print sum}')
        CLUSTER_COUNTS=$(wc -l ${sampleA}_vs_${sampleB}_radiolaria.uclust_* | grep "uclust" | awk 'BEGIN {ORS="|"} {print $1} END {print "\n"}' | head -n 1)
        echo "|${sampleA}_vs_${sampleB}|${region}|${total:-0}|${uniques:-0}|${radiolaria_total:-0}|${radiolaria_uniques}|${CLUSTER_COUNTS}"

        rm "${COMMON_READS}" "${RADIOLARIA_READS}" *_radiolaria.uclust_??

    done <<< "${replicates}"
    ```
  - Sample pooling   

    Compute the number of OTUs when pooling (not intersecting) the
    samples.

    ```
    ### Intersection of samples, taxonomy based filtering and clustering

    cd ./uchime/

    # List all replicate pairs
    replicates="Ei44_1_V4 Ei44_2_V4
    Ei44_1_V4 Ei45_V4
    Ei44_2_V4 Ei45_V4
    PEC16_1_V4 PEC16_2_V4
    SES11_V4 SES60_V4
    Ei44_1_V9 Ei44_2_V9
    Ei44_1_V9 Ei45_V9
    Ei44_2_V9 Ei45_V9
    PEC16_1_V9 PEC16_2_V9
    SES11_V9 SES60_V9"

    rm tmp*

    # Extract non-common reads, remove non-radiolarians, clusterize and
    # make a summary
    while read replicates ; do
        sampleA=${replicates% *}
        sampleB=${replicates#* }
        region=${replicates##*_}    
        all_samples="${sampleA}_dereplicated.fas ${sampleB}_dereplicated.fas"

        # Merge the reads
        POOLING=$(mktemp)
        cat ${all_samples} > "${POOLING}"

        # Limit to Radiolaria only
        RADIOLARIA_READS=$(mktemp)
        POOLING_RADIOLARIA=$(mktemp)    
        grep "^>" "${POOLING}" | tr -d ">" | cut -d "_" -f 1 |
        while read l ; do
            grep -m 1 "^$l" "all_${region}.results"
        done | grep "Radiolaria" | cut -f 1 | sort -du > "${RADIOLARIA_READS}"
        grep -A 1 -F -f "${RADIOLARIA_READS}" "${POOLING}" |
        sed -e '/^--$/d' > "${POOLING_RADIOLARIA}"

        # Clusterize
        USEARCH="usearch7.0.1001_i86linux32"
        TMP_USEARCH=$(mktemp)
        for THRESHOLD in {80..99} ; do
            OUTPUT_CLUSTERS="${sampleA}_plus_${sampleB}_radiolaria.uclust_${THRESHOLD}"
            "${USEARCH}" -cluster_smallmem -usersort "${POOLING_RADIOLARIA}" -id 0.${THRESHOLD} -uc "${TMP_USEARCH}" &> /dev/null
            grep "^S" "${TMP_USEARCH}" | 
            while read a b c d e f g h i j ; do
                hits=$(grep "^H.*${i}$" "${TMP_USEARCH}" | cut -f 9 | tr "\n" " " | sed -e 's/\ $//')
                echo "${i} ${hits}"
            done > "${OUTPUT_CLUSTERS}"
        done
        rm "${TMP_USEARCH}"
      
        # Parse clustering results (all levels, and 98%)
        total=$(awk 'BEGIN {FS = "_"} {sum += $2} END {print sum}' "${POOLING}")
        uniques=$(grep -c "^>" "${POOLING}")
        radiolaria_uniques=$(wc -l < "${RADIOLARIA_READS}")
        radiolaria_total=$(awk 'BEGIN {FS = "_"} {sum += $2} END {print sum}' "${POOLING_RADIOLARIA}")
        CLUSTER_COUNTS=$(wc -l ${sampleA}_plus_${sampleB}_radiolaria.uclust_* | grep "uclust" | awk 'BEGIN {ORS="|"} {print $1} END {print "\n"}' | head -n 1)
        echo "|${sampleA}_plus_${sampleB}|${region}|${total:-0}|${uniques:-0}|${radiolaria_total:-0}|${radiolaria_uniques}|${CLUSTER_COUNTS}"

        rm "${POOLING}" "${POOLING_RADIOLARIA}" "${RADIOLARIA_READS}" *_radiolaria.uclust_??

    done <<< "${replicates}"
    ```
  - Results   

    **Intersections after Acacia and Uchime**

    |  |  | All | All | Radiolaria | Radiolaria |  |  |  |  |  |  |  |  |  |  |  |  |  |  |  |  |  |  |  |  |
    | --- | --- | --- | --- | --- | --- | --- | --- | --- | --- | --- | --- | --- | --- | --- | --- | --- | --- | --- | --- | --- | --- | --- | --- | --- | --- |
    | Intersection of samples | region | Reads | Uniques | Reads | Uniques | 80 | 81 | 82 | 83 | 84 | 85 | 86 | 87 | 88 | 89 | 90 | 91 | 92 | 93 | 94 | 95 | 96 | 97 | 98 | 99 |
    | Ei44\_1\_V4\_vs\_Ei44\_2\_V4 | V4 | 1069 | 9 | 1016 | 6 | 1 | 1 | 1 | 1 | 1 | 1 | 1 | 1 | 1 | 1 | 1 | 1 | 1 | 1 | 1 | 1 | 1 | 1 | 1 | 3 |
    | Ei44\_1\_V4\_vs\_Ei45\_V4 | V4 | 331 | 3 | 329 | 2 | 1 | 1 | 1 | 1 | 1 | 1 | 1 | 1 | 1 | 1 | 1 | 1 | 1 | 1 | 1 | 1 | 1 | 1 | 1 | 2 |
    | Ei44\_2\_V4\_vs\_Ei45\_V4 | V4 | 976 | 4 | 972 | 3 | 1 | 1 | 1 | 1 | 1 | 1 | 1 | 1 | 1 | 1 | 1 | 1 | 1 | 1 | 1 | 1 | 1 | 1 | 1 | 2 |
    | PEC16\_1\_V4\_vs\_PEC16\_2\_V4 | V4 | 0 | 0 | 0 | 0 | 0 | 0 | 0 | 0 | 0 | 0 | 0 | 0 | 0 | 0 | 0 | 0 | 0 | 0 | 0 | 0 | 0 | 0 | 0 | 0 |
    | SES11\_V4\_vs\_SES60\_V4 | V4 | 35 | 3 | 0 | 0 | 0 | 0 | 0 | 0 | 0 | 0 | 0 | 0 | 0 | 0 | 0 | 0 | 0 | 0 | 0 | 0 | 0 | 0 | 0 | 0 |
    | Ei44\_1\_V9\_vs\_Ei44\_2\_V9 | V9 | 4365 | 37 | 1623 | 4 | 1 | 1 | 1 | 1 | 1 | 1 | 1 | 1 | 1 | 1 | 2 | 2 | 2 | 2 | 2 | 2 | 2 | 2 | 4 | 4 |
    | Ei44\_1\_V9\_vs\_Ei45\_V9 | V9 | 2521 | 18 | 1212 | 3 | 1 | 1 | 1 | 1 | 1 | 1 | 1 | 1 | 1 | 1 | 2 | 2 | 2 | 2 | 2 | 2 | 2 | 2 | 3 | 3 |
    | Ei44\_2\_V9\_vs\_Ei45\_V9 | V9 | 3297 | 24 | 1557 | 3 | 1 | 1 | 1 | 1 | 1 | 1 | 1 | 1 | 1 | 1 | 2 | 2 | 2 | 2 | 2 | 2 | 2 | 2 | 3 | 3 |
    | PEC16\_1\_V9\_vs\_PEC16\_2\_V9 | V9 | 2149 | 16 | 1610 | 2 | 1 | 1 | 1 | 1 | 1 | 1 | 1 | 1 | 1 | 1 | 1 | 1 | 1 | 1 | 1 | 1 | 1 | 1 | 1 | 1 |
    | SES11\_V9\_vs\_SES60\_V9 | V9 | 2251 | 13 | 0 | 0 | 0 | 0 | 0 | 0 | 0 | 0 | 0 | 0 | 0 | 0 | 0 | 0 | 0 | 0 | 0 | 0 | 0 | 0 | 0 | 0 |

    **Intersections without cleaning**

    |  |  | All | All | Radiolaria | Radiolaria |  |  |  |  |  |  |  |  |  |  |  |  |  |  |  |  |  |  |  |  |
    | --- | --- | --- | --- | --- | --- | --- | --- | --- | --- | --- | --- | --- | --- | --- | --- | --- | --- | --- | --- | --- | --- | --- | --- | --- | --- |
    | Intersected samples | region | Reads | Uniques | Reads | Uniques | 80 | 81 | 82 | 83 | 84 | 85 | 86 | 87 | 88 | 89 | 90 | 91 | 92 | 93 | 94 | 95 | 96 | 97 | 98 | 99 |
    | Ei44\_1\_V4\_vs\_Ei44\_2\_V4 | V4 | 2196 | 79 | 1981 | 45 | 1 | 1 | 1 | 1 | 1 | 1 | 1 | 1 | 1 | 1 | 1 | 1 | 1 | 1 | 1 | 1 | 2 | 2 | 5 | 10 |
    | Ei44\_1\_V4\_vs\_Ei45\_V4 | V4 | 1326 | 17 | 1294 | 10 | 1 | 1 | 1 | 1 | 1 | 1 | 1 | 1 | 1 | 1 | 1 | 1 | 1 | 1 | 1 | 1 | 1 | 1 | 1 | 2 |
    | Ei44\_2\_V4\_vs\_Ei45\_V4 | V4 | 1174 | 10 | 1167 | 7 | 1 | 1 | 1 | 1 | 1 | 1 | 1 | 1 | 1 | 1 | 1 | 1 | 1 | 1 | 1 | 1 | 1 | 2 | 2 | 3 |
    | PEC16\_1\_V4\_vs\_PEC16\_2\_V4 | V4 | 1131 | 32 | 1131 | 32 | 1 | 1 | 1 | 1 | 1 | 1 | 1 | 1 | 1 | 1 | 1 | 1 | 1 | 1 | 1 | 1 | 1 | 1 | 2 | 3 |
    | SES11\_V4\_vs\_SES60\_V4 | V4 | 125 | 19 | 36 | 7 | 1 | 1 | 1 | 1 | 1 | 1 | 1 | 1 | 1 | 1 | 1 | 1 | 1 | 1 | 1 | 1 | 1 | 1 | 1 | 2 |
    | Ei44\_1\_V9\_vs\_Ei44\_2\_V9 | V9 | 4277 | 62 | 1633 | 14 | 1 | 1 | 1 | 1 | 1 | 2 | 2 | 2 | 2 | 2 | 2 | 2 | 2 | 2 | 2 | 2 | 2 | 3 | 5 | 6 |
    | Ei44\_1\_V9\_vs\_Ei45\_V9 | V9 | 2552 | 37 | 1206 | 9 | 1 | 1 | 1 | 1 | 1 | 2 | 2 | 2 | 2 | 2 | 2 | 2 | 2 | 2 | 2 | 2 | 2 | 2 | 4 | 4 |
    | Ei44\_2\_V9\_vs\_Ei45\_V9 | V9 | 3309 | 49 | 1543 | 11 | 1 | 1 | 1 | 1 | 1 | 2 | 2 | 2 | 2 | 2 | 2 | 2 | 2 | 2 | 2 | 2 | 2 | 2 | 4 | 5 |
    | PEC16\_1\_V9\_vs\_PEC16\_2\_V9 | V9 | 2162 | 35 | 1635 | 14 | 1 | 1 | 1 | 1 | 1 | 2 | 2 | 2 | 2 | 2 | 2 | 2 | 2 | 2 | 3 | 3 | 3 | 3 | 4 | 4 |
    | SES11\_V9\_vs\_SES60\_V9 | V9 | 2305 | 23 | 0 | 0 | 0 | 0 | 0 | 0 | 0 | 0 | 0 | 0 | 0 | 0 | 0 | 0 | 0 | 0 | 0 | 0 | 0 | 0 | 0 | 0 |

    **Pooling after Acacia and Uchime**

    |  |  | All | All | Radiolaria | Radiolaria |  |  |  |  |  |  |  |  |  |  |  |  |  |  |  |  |  |  |  |  |
    | --- | --- | --- | --- | --- | --- | --- | --- | --- | --- | --- | --- | --- | --- | --- | --- | --- | --- | --- | --- | --- | --- | --- | --- | --- | --- |
    | Pooled samples | region | Reads | Uniques | Reads | Uniques | 80 | 81 | 82 | 83 | 84 | 85 | 86 | 87 | 88 | 89 | 90 | 91 | 92 | 93 | 94 | 95 | 96 | 97 | 98 | 99 |
    | Ei44\_1\_V4\_plus\_Ei44\_2\_V4 | V4 | 1282 | 125 | 1147 | 64 | 1 | 1 | 1 | 1 | 1 | 1 | 1 | 1 | 1 | 1 | 1 | 1 | 1 | 1 | 2 | 2 | 3 | 5 | 11 | 23 |
    | Ei44\_1\_V4\_plus\_Ei45\_V4 | V4 | 1493 | 160 | 1099 | 94 | 2 | 2 | 2 | 2 | 2 | 2 | 2 | 2 | 2 | 2 | 2 | 2 | 2 | 2 | 3 | 3 | 5 | 5 | 8 | 26 |
    | Ei44\_2\_V4\_plus\_Ei45\_V4 | V4 | 2357 | 227 | 1866 | 127 | 2 | 2 | 2 | 2 | 2 | 2 | 2 | 2 | 2 | 2 | 2 | 2 | 2 | 2 | 2 | 3 | 4 | 6 | 11 | 39 |
    | PEC16\_1\_V4\_plus\_PEC16\_2\_V4 | V4 | 13 | 8 | 13 | 8 | 1 | 1 | 1 | 1 | 1 | 1 | 1 | 1 | 1 | 1 | 1 | 1 | 1 | 1 | 1 | 1 | 1 | 1 | 2 | 3 |
    | SES11\_V4\_plus\_SES60\_V4 | V4 | 209 | 35 | 4 | 1 | 1 | 1 | 1 | 1 | 1 | 1 | 1 | 1 | 1 | 1 | 1 | 1 | 1 | 1 | 1 | 1 | 1 | 1 | 1 | 1 |
    | Ei44\_1\_V9\_plus\_Ei44\_2\_V9 | V9 | 4706 | 254 | 1657 | 25 | 2 | 2 | 2 | 2 | 2 | 2 | 2 | 2 | 2 | 2 | 3 | 3 | 3 | 3 | 4 | 4 | 4 | 7 | 9 | 13 |
    | Ei44\_1\_V9\_plus\_Ei45\_V9 | V9 | 2934 | 189 | 1233 | 21 | 1 | 1 | 1 | 1 | 1 | 1 | 1 | 1 | 1 | 1 | 2 | 2 | 2 | 2 | 3 | 3 | 3 | 5 | 8 | 11 |
    | Ei44\_2\_V9\_plus\_Ei45\_V9 | V9 | 3720 | 187 | 1578 | 14 | 2 | 2 | 2 | 2 | 2 | 2 | 2 | 2 | 2 | 2 | 3 | 3 | 3 | 3 | 4 | 4 | 4 | 5 | 8 | 10 |
    | PEC16\_1\_V9\_plus\_PEC16\_2\_V9 | V9 | 2262 | 90 | 1627 | 13 | 3 | 3 | 3 | 3 | 3 | 3 | 3 | 3 | 3 | 3 | 3 | 3 | 3 | 3 | 3 | 3 | 3 | 3 | 3 | 5 |
    | SES11\_V9\_plus\_SES60\_V9 | V9 | 4427 | 202 | 175 | 5 | 2 | 2 | 2 | 2 | 2 | 2 | 2 | 2 | 2 | 2 | 2 | 2 | 2 | 2 | 2 | 2 | 2 | 3 | 3 | 4 |

#### 3.2.3 AmpliconNoise quality filtering

- Folder preparation   

  ```
  cd results/
  mkdir ampliconnoise
  cd ampliconnoise/
  mkdir V4 V9
  cd V4/
  ln -s ../../data/AFB_10072012_Runs454/120627_XENON_HQC2HZF/ALI_AFBOTS_HQC2HZF02.sff ALI_AFBOTS_HQC2HZF02.sff
  cd ../V9/
  ln -s ../../data/AFB_10072012_Runs454/120627_XENON_HQC2HZF/ALI_AFBOTS_HQC2HZF02.sff ALI_AFBOTS_HQC2HZF02.sff
  ```

  **Flow**

  (from Roche's glossary)

  During a sequencing Run, nucleotides are flowed sequentially across
  the PTP device, one at a time, in the cyclical order "TACG', as
  controlled by the Run script. When the flowed nucleotide is a
  complementary to the next nucleotide (or homopolymer) on the DNA
  template in any given well, the polymerase extense the nascent DNA
  strand in that well. Addition of one or more nucleotide(s) releases a
  corresponding number of pyrophosphate (PPi) molecules. One molecule of
  ATP is synthesized for each PPi release, causing a flash of light
  (signal) whose intensive is proportional to the number of nucleotides
  incorporated.

  The link between sequence length and number of flows is not direct. A
  sequence made only of "Ts" is covered in one flow.

  I decide to consider only flowgrams that are 2/3 of the length of the
  average expected sequences. The V9 is around 150 bp, so 100 flows
  minimum. For the V4 (420 bp), its 300 flows minimum.
- Run AmpliconNoise   
  - V4   

    Prepare a key file and a primer file (can it work with IUPAC notation?
    Ask Christopher).

    ```
    cd ./results/ampliconnoise/V4/

    keys="EI44_1_V4,CAATAGG
    Ei44_2_V4,AACAACAA
    Ei45_V4,AGCATGCG
    PEC16_1_V4,CTTCTTCA
    PEC16_2_V4,AACAATGG
    Vil32_V4,GAGTACTA
    SES11_V4,TATCACAT
    SES60_V4,CCAGTCAG
    PAC_16_V4,CTATAAGT
    PAC_19_V4,ATGTATAA"

    echo "${keys}" > keys.csv

    # V4_Tom_F CCAGCASCYGCGGTAATTCC or CCAGCANCNGCGGTAATTCC
    echo -e ">V4_Tom_F\nCCAGCANCNGCGGTAATTCC" > primer.fasta
    ```

    Run the analysis

    ```
    cd ./results/ampliconnoise/V4/
    # Add usearch and sffinfo to the path
    export PATH=$PATH:$HOME
    export PATH=$HOME/AmpliconNoise/AmpliconNoiseV1.29/bin:$PATH
    export PATH=$HOME/AmpliconNoise/AmpliconNoiseV1.29/Scripts:$PATH
    export AMPLICON_NOISE_HOME=$HOME/AmpliconNoise/AmpliconNoiseV1.29/
    export PYRO_LOOKUP_FILE=$HOME/AmpliconNoise/AmpliconNoiseV1.29/Data/LookUp_Titanium.dat
    export SEQ_LOOKUP_FILE=$HOME/AmpliconNoise/AmpliconNoiseV1.29/Data/Tran.dat
    # Set the minflows value
    SCRIPT="${HOME}/AmpliconNoise/AmpliconNoiseV1.29/Scripts/RunTitanium.sh"
    sed -i 's/^minflows=[0-9]*$/minflows=300/' "${SCRIPT}"
    RunTitanium.sh all ALI_AFBOTS_HQC2HZF02.sff
    ```
  - V9   

    ```
    cd ./results/ampliconnoise/V9/

    keys="EI44_1_V9,ACGGAACC
    Ei44_2_V9,CGGAAGAC
    Ei45_V9,AATAGTCC
    PEC16_1_V9,AGGTATGG
    PEC16_2_V9,CCAACCTG
    Vil32_V9,TTCTAGTA
    SES11_V9,GAGAGCTG
    SES60_V9,TAGTATTC
    PAC_16_V9,AATATCGC
    PAC_19_V9,TCAGGTTC"

    echo "${keys}" > keys.csv

    # V9_F TTGTACACACCGCCC
    echo -e ">V9_F\nTTGTACACACCGCCC" > primer.fasta
    ```

    Run the analysis (I reduced the minflows to 240. A value of 400
    yielded zero V9 amplicons). The value I selected might not be optimal.

    ```
    cd ~/Science/Projects/Single_Cell_Radiolarians/results/ampliconnoise/V9/
    # Add usearch and sffinfo to the path
    export PATH=$PATH:$HOME
    export PATH=$HOME/AmpliconNoise/AmpliconNoiseV1.29/bin:$PATH
    export PATH=$HOME/AmpliconNoise/AmpliconNoiseV1.29/Scripts:$PATH
    export AMPLICON_NOISE_HOME=$HOME/AmpliconNoise/AmpliconNoiseV1.29/
    export PYRO_LOOKUP_FILE=$HOME/AmpliconNoise/AmpliconNoiseV1.29/Data/LookUp_Titanium.dat
    export SEQ_LOOKUP_FILE=$HOME/AmpliconNoise/AmpliconNoiseV1.29/Data/Tran.dat
    # Set the minflows value
    SCRIPT="${HOME}/AmpliconNoise/AmpliconNoiseV1.29/Scripts/RunTitanium.sh"
    sed -i 's/^minflows=[0-9]*$/minflows=100/' "${SCRIPT}"
    RunTitanium.sh all ALI_AFBOTS_HQC2HZF02.sff
    ```
- Remove reads without the reverse primer   

  Work on the "\_F\_Good.fa" files.

  ```
  # on my computer
  for REGION in V9 V4 ; do
      cd ./results/ampliconnoise/${REGION}/
      # Clean first
      rm -rf *.dat *_F_Chi.fa *.class *.fout *_F.per *.raw *.qual *.mapping *.master *.pout *_T400.fa *.fcout *.list *.otu *.snout *.seqdist *_cd.fa *_${REGION}.fa *_${REGION}_F.fa *.tree nonmatching.fasta All_Good* AN_stats.txt Temp.* splitkeys.stats *_s60/ *_s25/

      # Trim primer R and dereplicate (V9)
      for f in *_${REGION}_F_Good.fa ; do
          grep -B 1 "GTAGGTGAACCTGC[AG]GAAGG" "${f}" |
          sed -e 's/GTAGGTGAACCTGC[AG]GAAGG.*//' | sed -e '/^--$/d' | paste - - |
          awk 'BEGIN {FS = "\t"} {n = split($1, a, "_") ; for (i=1 ; i<=a[n] ; i++) {print $NF}}' |
          sort --temporary-directory=. -d | uniq -c |
          while read abundance sequence ; do
              hash=$(echo ${sequence} | sha1sum)
              hash=${hash:0:40}
              printf ">%s_%d_%s\n" "${hash}" "${abundance}" "${sequence}"
          done | sort -t "_" -k2,2nr | sed -e 's/\_/\n/2' > ${f/_F_Good.fa/_trimmed_dereplicated.fas}
      done
  done
  ```
- Remove non-radiolarians   
  - merge and dereplicate all V4 and all V9   

    ```
    for REGION in V9 V4 ; do
        cd ./results/ampliconnoise/${REGION}/
        FASTA="ampliconnoise_all_${REGION}.fas"
        ## Dereplicate the whole project (using a Awk table)
        cat *_trimmed_dereplicated.fas |
        awk 'BEGIN {RS = ">" ; FS = "[_\n]"} {if (NR != 1) {abundances[$1] += $2 ; sequences[$1] = $3}} END {for (amplicon in sequences) {print ">" amplicon "_" abundances[amplicon] "_" sequences[amplicon]}}' |
        sort --temporary-directory=$(pwd) -t "_" -k2,2nr -k1.2,1d |
        sed -e 's/\_/\n/2' > "${FASTA}"
    done
    ```

    Perform taxonomic assignment (see Acacia section for details)
  - filter out non-radiolarians and produce the summary table   

    ```
    FOLDER="./results/ampliconnoise/"
    # List Rhizaria hits in each sample
    ASSIGNMENTS=$(mktemp)
    for REGION in V4 V9 ; do
        cd "${FOLDER}${REGION}"
        TAXO_RESULTS="../../Stampa/ampliconnoise_all_${REGION}.results"
        for f in *_${REGION}_F_Good.fa ; do
            TRIMMED=${f/_F_Good.fa/_trimmed_dereplicated.fas}
            SAMPLE=${f/_V*_F_Good.fa/}
            echo -en "${SAMPLE}\t${REGION}\t"
            for FILE in ${f} ${TRIMMED} ; do
                awk 'BEGIN {FS = "_" ; OFS = "\t" ; ORS = "" ; uniq = 0 ; sum = 0} /^>/ {uniq += 1 ; sum += $NF} END {print sum, uniq, ""}' ${FILE}
            done
            grep "^>" "${TRIMMED}" | tr -d ">" | tr "_" " " |
            while read id ab ; do
                taxo=$(grep -m 1 "^${id}" "${TAXO_RESULTS}" | cut -f 3-4)
                echo -e "${id}\t${ab}\t${taxo}"
            done > "${ASSIGNMENTS}"
            grep "Radiolaria" "${ASSIGNMENTS}" | awk 'BEGIN {OFS = "\t" ; ORS = "" ; sum=0} {sum+=$2} END {print sum, NR, ""}'
            grep "Radiolaria" "${ASSIGNMENTS}" |
            while read id ab taxo ; do
                grep -m 1 -A 1 "^>${id}" "${TRIMMED}"
            done > "${TRIMMED/.fas/_radiolaria.fas}"
            grep -v "Radiolaria" "${ASSIGNMENTS}" | awk 'BEGIN {OFS = "\t"; sum=0} {sum+=$2} END {print sum, NR}'
        done
    done
    rm "${ASSIGNMENTS}"
    ```
- Summary of filtering results   

  | Taxa | region | AmpliconNoise (T) | AmpliconNoise (U) | distal (T) | distal (U) | Radiolaria (T) | Radiolaria (U) | Other (T) | Other (U) |
  | --- | --- | --- | --- | --- | --- | --- | --- | --- | --- |
  | EI44\_1 | V4 | 463 | 25 | 408 | 7 | 386 | 4 | 22 | 3 |
  | Ei44\_2 | V4 | 3059 | 83 | 2797 | 21 | 2594 | 3 | 203 | 18 |
  | Ei45 | V4 | 2974 | 51 | 2809 | 19 | 1985 | 4 | 824 | 15 |
  | PEC16\_1 | V4 | 203 | 3 | 0 | 0 | 0 | 0 | 0 | 0 |
  | PEC16\_2 | V4 | 73 | 2 | 0 | 0 | 0 | 0 | 0 | 0 |
  | SES11 | V4 | 983 | 29 | 288 | 8 | 0 | 0 | 288 | 8 |
  | SES60 | V4 | 78 | 8 | 54 | 3 | 0 | 0 | 54 | 3 |
  | Vil32 | V4 | 1279 | 11 | 1247 | 2 | 1226 | 1 | 21 | 1 |
  | Ei44\_1 | V9 | 1167 | 72 | 1156 | 53 | 30 | 3 | 1126 | 50 |
  | Ei44\_2 | V9 | 2244 | 89 | 2225 | 64 | 589 | 4 | 1636 | 60 |
  | Ei45 | V9 | 715 | 46 | 713 | 40 | 369 | 3 | 344 | 37 |
  | PEC16\_1 | V9 | 1214 | 38 | 1207 | 25 | 899 | 4 | 308 | 21 |
  | PEC16\_2 | V9 | 1052 | 26 | 1050 | 21 | 808 | 1 | 242 | 20 |
  | SES11 | V9 | 2563 | 74 | 2555 | 56 | 64 | 1 | 2491 | 55 |
  | SES60 | V9 | 1751 | 49 | 1742 | 31 | 116 | 2 | 1626 | 29 |
  | Vil32 | V9 | 1258 | 28 | 1254 | 21 | 1083 | 3 | 171 | 18 |

  legend: T = Total number of reads; U = unique reads

  AmpliconNoise saves no PEC and SES sequences.
- Clustering with Usearch   

  To get a precise idea of the content of each sample, we used uclust
  (usearch7.0.1001\_i86linux32) to compute the number of OTUs for all
  clustering levels from 80% to 99%.

  ```
  # Extract Radiolaria and clusterize
  USEARCH="usearch7.0.1001_i86linux32"
  TMP_USEARCH=$(mktemp)
  # FILENAME="trimmed_dereplicated_radiolaria"
  FILENAME="trimmed_dereplicated_radiolaria_no_crosscontaminations"
  for REGION in V9 V4 ; do
      cd ~/Science/Projects/Single_Cell_Radiolarians/results/ampliconnoise/${REGION}/
      for f in *_${REGION}_${FILENAME}.fas ; do
          for THRESHOLD in {80..99} ; do
              OUTPUT_CLUSTERS="${f/.fas/.uclust_}${THRESHOLD}"
              "${USEARCH}" -usersort -cluster_smallmem "${f}" -id 0.${THRESHOLD} -uc "${TMP_USEARCH}" 2> /dev/null > /dev/null
              grep "^S" "${TMP_USEARCH}" | 
              while read a b c d e f g h i j ; do
                  hits=$(grep "^H.*${i}$" "${TMP_USEARCH}" | cut -f 9 | tr "\n" " " | sed -e 's/\ $//')
                  echo "${i} ${hits}"
              done > "${OUTPUT_CLUSTERS}"
          done
          READS=$(awk 'BEGIN {FS = "_" ; OFS = "\t" ; uniq = 0 ; sum = 0} /^>/ {uniq += 1 ; sum += $2} END {print sum, uniq}' "${f}")
          SAMPLE=${f/_V*_${FILENAME}.fas/}
          CLUSTER_COUNTS=$(wc -l ${SAMPLE}_${REGION}_${FILENAME}.uclust_* | grep "uclust" | awk 'BEGIN {ORS = "\t"} {print $1}' | sed -e 's/\t$//')
          echo -e "${SAMPLE}\t${REGION}\t${READS}\t${CLUSTER_COUNTS}"
          rm -f ${SAMPLE}_${REGION}_${FILENAME}.uclust_*
      done
  done
  rm "${TMP_USEARCH}"
  ```

  Number of OTUs at different clustering thresholds (Uniques column
  represent the number of OTUs at 100%).

  | Sample | Region | Reads | Uniques | 80 | 81 | 82 | 83 | 84 | 85 | 86 | 87 | 88 | 89 | 90 | 91 | 92 | 93 | 94 | 95 | 96 | 97 | 98 | 99 |
  | --- | --- | --- | --- | --- | --- | --- | --- | --- | --- | --- | --- | --- | --- | --- | --- | --- | --- | --- | --- | --- | --- | --- | --- |
  | Ei44\_1 | V9 | 30 | 3 | 1 | 1 | 1 | 1 | 1 | 1 | 1 | 1 | 1 | 1 | 2 | 2 | 2 | 2 | 3 | 3 | 3 | 3 | 3 | 3 |
  | Ei44\_2 | V9 | 589 | 4 | 2 | 2 | 2 | 2 | 2 | 2 | 2 | 2 | 2 | 2 | 3 | 3 | 3 | 3 | 3 | 3 | 3 | 3 | 3 | 3 |
  | Ei45 | V9 | 369 | 3 | 1 | 1 | 1 | 1 | 1 | 1 | 1 | 1 | 1 | 1 | 2 | 2 | 2 | 2 | 3 | 3 | 3 | 3 | 3 | 3 |
  | PEC16\_1 | V9 | 899 | 4 | 3 | 3 | 3 | 3 | 3 | 3 | 3 | 3 | 3 | 3 | 3 | 3 | 3 | 3 | 3 | 3 | 3 | 3 | 3 | 4 |
  | PEC16\_2 | V9 | 808 | 1 | 1 | 1 | 1 | 1 | 1 | 1 | 1 | 1 | 1 | 1 | 1 | 1 | 1 | 1 | 1 | 1 | 1 | 1 | 1 | 1 |
  | SES11 | V9 | 64 | 1 | 1 | 1 | 1 | 1 | 1 | 1 | 1 | 1 | 1 | 1 | 1 | 1 | 1 | 1 | 1 | 1 | 1 | 1 | 1 | 1 |
  | SES60 | V9 | 116 | 2 | 2 | 2 | 2 | 2 | 2 | 2 | 2 | 2 | 2 | 2 | 2 | 2 | 2 | 2 | 2 | 2 | 2 | 2 | 2 | 2 |
  | Vil32 | V9 | 1083 | 3 | 2 | 2 | 2 | 2 | 2 | 2 | 2 | 2 | 3 | 3 | 3 | 3 | 3 | 3 | 3 | 3 | 3 | 3 | 3 | 3 |
  | Ei44\_1 | V4 | 386 | 4 | 1 | 1 | 1 | 1 | 1 | 1 | 1 | 2 | 2 | 2 | 2 | 2 | 2 | 2 | 3 | 3 | 4 | 4 | 4 | 4 |
  | Ei44\_2 | V4 | 2594 | 3 | 1 | 1 | 1 | 1 | 1 | 1 | 1 | 1 | 1 | 1 | 1 | 1 | 1 | 1 | 1 | 1 | 3 | 3 | 3 | 3 |
  | Ei45 | V4 | 1985 | 4 | 2 | 2 | 2 | 2 | 2 | 2 | 2 | 2 | 2 | 2 | 2 | 2 | 2 | 2 | 2 | 2 | 2 | 3 | 3 | 3 |
  | PEC16\_1 | V4 | 0 | 0 | 0 | 0 | 0 | 0 | 0 | 0 | 0 | 0 | 0 | 0 | 0 | 0 | 0 | 0 | 0 | 0 | 0 | 0 | 0 | 0 |
  | PEC16\_2 | V4 | 0 | 0 | 0 | 0 | 0 | 0 | 0 | 0 | 0 | 0 | 0 | 0 | 0 | 0 | 0 | 0 | 0 | 0 | 0 | 0 | 0 | 0 |
  | SES11 | V4 | 0 | 0 | 0 | 0 | 0 | 0 | 0 | 0 | 0 | 0 | 0 | 0 | 0 | 0 | 0 | 0 | 0 | 0 | 0 | 0 | 0 | 0 |
  | SES60 | V4 | 0 | 0 | 0 | 0 | 0 | 0 | 0 | 0 | 0 | 0 | 0 | 0 | 0 | 0 | 0 | 0 | 0 | 0 | 0 | 0 | 0 | 0 |
  | Vil32 | V4 | 1226 | 1 | 1 | 1 | 1 | 1 | 1 | 1 | 1 | 1 | 1 | 1 | 1 | 1 | 1 | 1 | 1 | 1 | 1 | 1 | 1 | 1 |

  **After cross-contamination cleaning**

  | Sample | Region | Reads | Uniques | 80 | 81 | 82 | 83 | 84 | 85 | 86 | 87 | 88 | 89 | 90 | 91 | 92 | 93 | 94 | 95 | 96 | 97 | 98 | 99 |
  | --- | --- | --- | --- | --- | --- | --- | --- | --- | --- | --- | --- | --- | --- | --- | --- | --- | --- | --- | --- | --- | --- | --- | --- |
  | Ei44\_1 | V9 | 30 | 3 | 1 | 1 | 1 | 1 | 1 | 1 | 1 | 1 | 1 | 1 | 2 | 2 | 2 | 2 | 3 | 3 | 3 | 3 | 3 | 3 |
  | Ei44\_2 | V9 | 587 | 2 | 1 | 1 | 1 | 1 | 1 | 1 | 1 | 1 | 1 | 1 | 2 | 2 | 2 | 2 | 2 | 2 | 2 | 2 | 2 | 2 |
  | Ei45 | V9 | 369 | 3 | 1 | 1 | 1 | 1 | 1 | 1 | 1 | 1 | 1 | 1 | 2 | 2 | 2 | 2 | 3 | 3 | 3 | 3 | 3 | 3 |
  | PEC16\_1 | V9 | 887 | 1 | 1 | 1 | 1 | 1 | 1 | 1 | 1 | 1 | 1 | 1 | 1 | 1 | 1 | 1 | 1 | 1 | 1 | 1 | 1 | 1 |
  | PEC16\_2 | V9 | 808 | 1 | 1 | 1 | 1 | 1 | 1 | 1 | 1 | 1 | 1 | 1 | 1 | 1 | 1 | 1 | 1 | 1 | 1 | 1 | 1 | 1 |
  | SES11 | V9 | 64 | 1 | 1 | 1 | 1 | 1 | 1 | 1 | 1 | 1 | 1 | 1 | 1 | 1 | 1 | 1 | 1 | 1 | 1 | 1 | 1 | 1 |
  | SES60 | V9 | 110 | 1 | 1 | 1 | 1 | 1 | 1 | 1 | 1 | 1 | 1 | 1 | 1 | 1 | 1 | 1 | 1 | 1 | 1 | 1 | 1 | 1 |
  | Vil32 | V9 | 1080 | 1 | 1 | 1 | 1 | 1 | 1 | 1 | 1 | 1 | 1 | 1 | 1 | 1 | 1 | 1 | 1 | 1 | 1 | 1 | 1 | 1 |
  | Ei44\_1 | V4 | 386 | 4 | 1 | 1 | 1 | 1 | 1 | 1 | 1 | 2 | 2 | 2 | 2 | 2 | 2 | 2 | 3 | 3 | 4 | 4 | 4 | 4 |
  | Ei44\_2 | V4 | 2594 | 3 | 1 | 1 | 1 | 1 | 1 | 1 | 1 | 1 | 1 | 1 | 1 | 1 | 1 | 1 | 1 | 1 | 3 | 3 | 3 | 3 |
  | Ei45 | V4 | 1984 | 3 | 1 | 1 | 1 | 1 | 1 | 1 | 1 | 1 | 1 | 1 | 1 | 1 | 1 | 1 | 1 | 1 | 1 | 2 | 2 | 2 |
  | PEC16\_1 | V4 | 0 | 0 | 0 | 0 | 0 | 0 | 0 | 0 | 0 | 0 | 0 | 0 | 0 | 0 | 0 | 0 | 0 | 0 | 0 | 0 | 0 | 0 |
  | PEC16\_2 | V4 | 0 | 0 | 0 | 0 | 0 | 0 | 0 | 0 | 0 | 0 | 0 | 0 | 0 | 0 | 0 | 0 | 0 | 0 | 0 | 0 | 0 | 0 |
  | SES11 | V4 | 0 | 0 | 0 | 0 | 0 | 0 | 0 | 0 | 0 | 0 | 0 | 0 | 0 | 0 | 0 | 0 | 0 | 0 | 0 | 0 | 0 | 0 |
  | SES60 | V4 | 0 | 0 | 0 | 0 | 0 | 0 | 0 | 0 | 0 | 0 | 0 | 0 | 0 | 0 | 0 | 0 | 0 | 0 | 0 | 0 | 0 | 0 |
  | Vil32 | V4 | 1226 | 1 | 1 | 1 | 1 | 1 | 1 | 1 | 1 | 1 | 1 | 1 | 1 | 1 | 1 | 1 | 1 | 1 | 1 | 1 | 1 | 1 |
- Verification of taxonomic assignments   

  There are some cross-contaminations that I forgot to remove from the
  final fasta file (Radiolaria, but not the targetted species).

  ```
  for REGION in V4 V9 ; do
      cd ./results/ampliconnoise/${REGION}/
      for f in *_${REGION}_trimmed_dereplicated_radiolaria.fas ; do
          echo "## ${f}"
          cut -d "_" -s -f 1 "${f}" | tr -d ">" |
          while read l ; do
              grep "$l" ../../Stampa/ampliconnoise_all_${REGION}.results
          done
          echo
      done
  done
  ```

  I need to prepare new fasta files without cross-contaminations:

  ```
  for REGION in V4 V9 ; do
      cd ~/Science/Projects/Single_Cell_Radiolarians/results/ampliconnoise/${REGION}/
      for f in *_${REGION}_trimmed_dereplicated_radiolaria.fas ; do
          cp "${f}" "${f/.fas/_no_crosscontaminations.fas}"
      done
  done
  ```

  **Remove**

  Ei 44\_2\_V9
  76933c57caa9c7f5ab19ba24e0bd029e70f1bcd5 1 84.1 Eukaryota|Rhizaria|Radiolaria|Acanth B2|\*|\*|\*|\* GU246585
  d015ec81ec5730a976593e98cd4c24affd019eb3 1 83.3 Eukaryota|Rhizaria|Radiolaria|Acanth B2|\*|\*|\*|\* GU246585

  PEC 16\_1\_V9
  4971d07e41d8c63b3aab8546a58a4953b5aeb3da 7 92.2 Eukaryota|Rhizaria|Radiolaria|Polycystinea|Collodaria-Nassellarida|Collodaria|Siphonosphaera|cyathina AF091145
  b7ada2fb38a7c42ba87b42ba4796f6bb65af0857 4 90.8 Eukaryota|Rhizaria|Radiolaria|Polycystinea|Collodaria-Nassellarida|Collodaria|Collozoum|pelagicum AF091146
  c6bfb286a6b6fc362e9be3524f05f1f8dcc432d1 11 92.4 Eukaryota|Rhizaria|Radiolaria|Polycystinea|Collodaria-Nassellarida|Collodaria|Collozoum|pelagicum AF091146

  Ses 60\_V9
  6fb106064a06036f57756e15e22b729a07f833ba 18 94.7 Eukaryota|Rhizaria|Radiolaria|Polycystinea|Collodaria-Nassellarida|Collodaria|Collozoum|inerme AY266295

  Vil32\_V9
  fc5812f0a8d0dc8108476eefe26063f9c529b645 2 96.2 Eukaryota|Rhizaria|Radiolaria|Polycystinea|Collodaria-Nassellarida|Collodaria|Collozoum|\* AF091146,AY266295
  a2058947e9d0e0a153f24742e1ab3fbe9efd243b 40355 97.7 Eukaryota|Rhizaria|Radiolaria|Polycystinea|Collodaria-Nassellarida|Collodaria|Sphaerozoum|punctatum AF018161

  Ei 45\_V4
  487f9b6da71fc8f47db216d522083c65d744ecb1 1 93.3 Eukaryota|Rhizaria|Radiolaria|Polycystinea|Collodaria-Nassellarida|Collodaria|Sphaerozoum|Sphaerozoum+punctatum|Sphaerozoum|Sphaerozoum+punctatum AF018161.1.1788\_U

### 3.3 Analysis without denoising

Here we start from the fasta file extracted from the initial SFF file.

```
sffinfo -s ALI_AFBOTS_HQC2HZF02.sff > ALI_AFBOTS_HQC2HZF02.fas
```

#### 3.3.1 Parse reads into samples

The samples are tagged as follow:

| Sample\_ID | Primer\_name | MIDnb | Sequences | Taxa |
| --- | --- | --- | --- | --- |
| Ei44\_1 | V4Lf 7R0227 | 7R0227 | caatagg | Acantharea |
| Ei44\_2 | V4Lf 8R1 | 8R0001 | aacaacaa | Acantharea |
| Ei45 | V4Lf 8R5 | 8R0005 | agcatgcg | Acantharea |
| PEC16\_1 | V4Lf 8R8 | 8R0008 | cttcttca | Acantharea |
| PEC16\_2 | V4Lf 8R10 | 8R0010 | aacaatgg | Acantharea |
| Vil32 | V4Lf 8R12 | 8R0012 | gagtacta | Acantharea |
| SES11 | V4Lf 8R15 | 8R0015 | tatcacat | Nassellarida |
| SES60 | V4Lf 8R20 | 8R0020 | ccagtcag | Nassellarida |
| Ei44\_1 | V9Lf 8R0757 | 8R0757 | acggaacc | Acantharea |
| Ei44\_2 | V9Lf 8R0758 | 8R0758 | cggaagac | Acantharea |
| Ei45 | V9Lf 8R0744 | 8R0744 | aatagtcc | Acantharea |
| PEC16\_1 | V9Lf 8R0748 | 8R0748 | aggtatgg | Acantharea |
| PEC16\_2 | V9Lf 8R0749 | 8R0749 | ccaacctg | Acantharea |
| Vil32 | V9Lf 8R0751 | 8R0751 | ttctagta | Acantharea |
| SES11 | V9Lf 8R0752 | 8R0752 | gagagctg | Nassellarida |
| SES60 | V9Lf 8R0754 | 8R0754 | tagtattc | Nassellarida |

Extract reads starting with one of our MIDs, the rest is thrown
away. Each MID corresponds to a pair of primers, and we search
amplicons containing both forward and reverse primers, allowing up to
two errors in the primer sequences and one error in the MID
sequence. We use the module MOODS too search for approximate sequence,
but encountered some bugs and annoying "segmentation fault". We are
looking for a replacement solution for future analyses.

```
python extract_and_ventilate_reads.py -i ASD_ACAOTS_HBGGA4404.fas
```

The python script is reproduced below:

```
#!/usr/bin/env python
# -*- coding: utf-8 -*-
"""
    Extract and ventilate reads.
"""

from __future__ import print_function

__author__ = "Frédéric Mahé <mahe@rhrk.uni-kl.de>"
__date__ = "2012/12/11"
__version__ = "$Revision: 1.0"

import os
import sys
import MOODS
from Bio import SeqIO
from Bio.Seq import Seq
from Bio.Alphabet import IUPAC
from optparse import OptionParser

samples = [
("7R0227", "caatagg", "V4"),
("8R0001", "aacaacaa", "V4"),
("8R0005", "agcatgcg", "V4"),
("8R0008", "cttcttca", "V4"),
("8R0010", "aacaatgg", "V4"),
("8R0012", "gagtacta", "V4"),
("8R0015", "tatcacat", "V4"),
("8R0020", "ccagtcag", "V4"),
("8R0757", "acggaacc", "V9"),
("8R0758", "cggaagac", "V9"),
("8R0744", "aatagtcc", "V9"),
("8R0748", "aggtatgg", "V9"),
("8R0749", "ccaacctg", "V9"),
("8R0751", "ttctagta", "V9"),
("8R0752", "gagagctg", "V9"),
("8R0754", "tagtattc", "V9")]

#**********************************************************************#
#                                                                      #
#                            Functions                                 #
#                                                                      #
#**********************************************************************#

def  option_parse():
    """
    Parse arguments from command line.
    """
    parser = OptionParser(usage="usage: %prog --input_file filename",
        version="%prog 1.0")

    parser.add_option("-i", "--input_file",
        metavar="FILE",
        action="store",
        dest="input_file",
        help="set FILE as input")

    (options, args) = parser.parse_args()

    return options.input_file


def primer2pwm(primer):
    """
    Write a primer sequence as a position weight matrix.
    """
    # Create 4 lists of length equal to primer's length.
    matrix = [[0] * len(primer) for i in range(4)]
    # List of correspondance IUPAC.
    IUPAC = {
        "A" : ["A"], 
        "C" : ["C"], 
        "G" : ["G"],        
        "T" : ["T"],        
        "U" : ["U"],        
        "R" : ["G", "A"], 
        "Y" : ["T", "C"], 
        "K" : ["G", "T"], 
        "M" : ["A", "C"], 
        "S" : ["G", "C"], 
        "W" : ["A", "T"], 
        "B" : ["C", "G", "T"], 
        "D" : ["A", "G", "T"], 
        "H" : ["A", "C", "T"], 
        "V" : ["A", "C", "G"], 
        "N" : ["A", "C", "G", "T"]
    }
    # Position of nucleotides in the PWM.
    dico = {"A" : 0,  "C" : 1, "G" : 2, "T" : 3}
    # Read each IUPAC letter in the primer.
    for index, letter in enumerate(primer):
        for nuc in IUPAC.get(letter):
            i = dico.get(nuc)
            matrix[i][index] = 1
    return matrix

#**********************************************************************#
#                                                                      #
#                              Body                                    #
#                                                                      #
#**********************************************************************#

if __name__ == '__main__':

    input_file = option_parse()
    input_format = "fasta"
    with open(input_file, "rU") as input_file:
        records = SeqIO.parse(input_file, input_format)
        records_list = [(record.id, len(record.seq), str(record.seq.lower())) for record in records]

    MIDs = [sample[0] for sample in samples]
    sequences = [sample[1] for sample in samples]
    regions = [sample[2] for sample in samples]
    sequences_to_MIDs = dict(zip(sequences,MIDs))
    MIDs_to_sequences = dict(zip(MIDs,sequences))
    MIDs_to_regions = dict(zip(MIDs,regions))
    sequences = set(sequences)
    MIDs = set(MIDs)

    # Initialize storage structures
    weird_reads = list()
    samples_dict = dict()

    # Parse the reads 
    for record in records_list:
        seq8 = record[2][0:8]
        seq7 = record[2][0:7] # I have a 7-nucleotides MID
        if seq7 in sequences:
            sample_id = sequences_to_MIDs[seq7]
            samples_dict.setdefault(sample_id,[]).append(record)
        elif seq8 in sequences:
            sample_id = sequences_to_MIDs[seq8]
            samples_dict.setdefault(sample_id,[]).append(record)
        else:
            # Bad read
            weird_reads.append(record)

    print("Reads not starting with a MID:", len(weird_reads))

    # Prepare PWM
    primers = dict()
    # V9
    forward_primer = "TTGTACACACCGCCC"
    forward_threshold = len(forward_primer) - 1
    forward_matrix = primer2pwm(forward_primer)
    reverse_primer = "GTAGGTGAACCTGCRGAAGG"
    reverse_threshold = len(reverse_primer) - 2
    reverse_matrix = primer2pwm(reverse_primer)
    primers["V9"] = (forward_primer, forward_threshold, forward_matrix, reverse_primer, reverse_threshold, reverse_matrix)
    # V4
    forward_primer = "CCAGCASCYGCGGTAATTCC"
    forward_threshold = len(forward_primer) - 2
    forward_matrix = primer2pwm(forward_primer)
    reverse_primer = "TYRATCAAGAACGAAAGT"
    reverse_threshold = len(reverse_primer) - 2
    reverse_matrix = primer2pwm(reverse_primer)
    primers["V4"] = (forward_primer, forward_threshold, forward_matrix, reverse_primer, reverse_threshold, reverse_matrix)
    
    # Output
    extension = ".fas"
    i=1
    # Those reads trigger a segmentation fault in MOODs. I have no
    # idea why!?
    banned = set(["HQC2HZF02DG8O6", "HQC2HZF02EE9YO", "HQC2HZF02EX9OU", "HQC2HZF02ELANW", "HQC2HZF02C32ZD", "HQC2HZF02EIAMD", "HQC2HZF02C4LVE", "HQC2HZF02DA669", "HQC2HZF02EBFGM", "HQC2HZF02C9CES", "HQC2HZF02DHPAF", "HQC2HZF02DHXWI", "HQC2HZF02D2H7H", "HQC2HZF02C9RVP", "HQC2HZF02C0NTC", "HQC2HZF02DWDCX", "HQC2HZF02DJTM4", "HQC2HZF02DQLS0", "HQC2HZF02DDZ7N", "HQC2HZF02E0HJP", "HQC2HZF02DSJA3", "HQC2HZF02DTFW1", "HQC2HZF02C70WF", "HQC2HZF02EASMH", "HQC2HZF02C22XH", "HQC2HZF02EUBCW", "HQC2HZF02ENH1E", "HQC2HZF02EFDX6", "HQC2HZF02D186H", "HQC2HZF02DS89D", "HQC2HZF02DEOO1", "HQC2HZF02C0QHC", "HQC2HZF02EMRNL", "HQC2HZF02D969X", "HQC2HZF02DR0B9", "HQC2HZF02D8MIB", "HQC2HZF02DF2ZP", "HQC2HZF02EF0V1", "HQC2HZF02D4EF0", "HQC2HZF02DJ53L", "HQC2HZF02DYU6C", "HQC2HZF02EOJK1", "HQC2HZF02DBE9M", "HQC2HZF02DJLPX", "HQC2HZF02D1XK9", "HQC2HZF02EEPDN", "HQC2HZF02DKA8Y"])
    for sample_id in samples_dict:
        # Get region V4 or V9
        region = MIDs_to_regions[sample_id]
        # start = len(MIDs_to_sequences[sample_id])
        # How many raw reads
        print(sample_id, len(samples_dict[sample_id]), file=sys.stdout)
        # Get parameters for this region
        forward_primer, forward_threshold, forward_matrix, reverse_primer, reverse_threshold, reverse_matrix = primers[region]
        output_file = sample_id + extension
        with open(output_file, "w") as output_file:
            for record in samples_dict[sample_id]:
                if record[0] not in banned:
                    # Test presence of both primers
                    results_forward = MOODS.search(record[2], [forward_matrix], forward_threshold, absolute_threshold=forward_threshold)
                    if len(results_forward[0]) == 1:
                        (position, score) = results_forward[0][0]
                        start = position + len(forward_primer)
                        # print(start, len(record[2]))
                        if start < len(record[2]):
                            results_reverse = MOODS.search(record[2], [reverse_matrix], reverse_threshold, absolute_threshold=reverse_threshold)
                            if len(results_reverse[0]) == 1:
                                (position, score) = results_reverse[0][0]
                                end = position
                                print(">", record[0], "_1" , "\n", record[2][start:end], sep="", file=output_file)
    
sys.exit(0)
```

Validated amplicons were distributed in files named after the sample
they refer to:

```
# Example of file:
Ei44_1_V4.fas
```

#### 3.3.2 Dereplicate

In each sample, merge strictly identical sequences.

```
for f in *.fas ; do
    grep -v "^>" "${f}" | sort -d | uniq -c |
    while read abundance sequence ; do
        hash=$(sha1sum <<< "${sequence}")
        hash=${hash:0:40}
        printf ">%s_%d_%s\n" "${hash}" "${abundance}" "${sequence}"
    done | sort -t "_" -k2,2nr -k1.2,1d |
    sed -e 's/\_/\n/2' > "${f/.fas/_dereplicated.fas}"
done
```

#### 3.3.3 Results per sample

Build a table of results (no quality filtering Acacia, no chimera
checking, no filtering based on taxonomy)

```
for f in *[49].fas ; do
    amplicons=$(grep -c "^>" "${f}")
    dereplicated=$(grep -c "^>" "${f/.fas/_dereplicated.fas}")
    region=${f/.fas/}
    region=${region##*_}
    taxa=${f/_V*/}
    echo "|${taxa}|${region}|${amplicons}|${dereplicated}|"
done | sort -t "|" -k2,2d
```

| Sample | region | reads | uniques |
| --- | --- | --- | --- |
| Ei44\_1 | V4 | 1983 | 453 |
| Ei44\_2 | V4 | 1809 | 414 |
| Ei45 | V4 | 1561 | 347 |
| PAC\_16 | V4 | 13702 | 5332 |
| PAC\_19 | V4 | 10657 | 3430 |
| PEC16\_1 | V4 | 925 | 169 |
| PEC16\_2 | V4 | 422 | 91 |
| SES11 | V4 | 781 | 297 |
| SES60 | V4 | 125 | 62 |
| Vil32 | V4 | 801 | 136 |
| Ei44\_1 | V9 | 2189 | 312 |
| Ei44\_2 | V9 | 2830 | 290 |
| Ei45 | V9 | 1012 | 133 |
| PAC\_16 | V9 | 18864 | 1550 |
| PAC\_19 | V9 | 33748 | 1773 |
| PEC16\_1 | V9 | 1265 | 105 |
| PEC16\_2 | V9 | 1116 | 111 |
| SES11 | V9 | 2823 | 360 |
| SES60 | V9 | 1849 | 177 |
| Vil32 | V9 | 1514 | 87 |

#### 3.3.4 Taxonomic assignment of the reads

We re-used the taxonomic assignment method described in the denoising
section. Here are the results of a taxonomy-based filtering
(Radiolaria or not):

```
RADIOLARIA=$(mktemp)
for f in *[49]_dereplicated.fas ; do
    amplicons=$(grep -c "^>" "${f/_dereplicated/}")
    dereplicated=$(grep -c "^>" "${f}")
    region=${f/_dereplicated.fas/}
    region=${region##*_}
    taxa=${f/_V*/}
    ## Only radiolarians
    grep "^>" "${f}" | tr -d ">" | cut -d "_" -f 1 |
    while read read_id ; do
        grep -h -m 1 "${read_id}" all_V*.results
    done | grep "Radiolaria" | cut -f 1 > "${RADIOLARIA}"
    radiolaria=$(grep -F -f "${RADIOLARIA}" "${f}" | awk 'BEGIN {FS = "_"} {sum+=$2} END {print sum "|" NR}')
    echo "|${taxa}|${region}|${amplicons}|${dereplicated}|${radiolaria}|"
done | sort -t "|" -k3,3d -k2,2d
rm "${RADIOLARIA}"
```

| Sample | region | reads | uniques | Radiolaria | uniques |
| --- | --- | --- | --- | --- | --- |
| Ei44\_1 | V4 | 1983 | 453 | 1711 | 261 |
| Ei44\_2 | V4 | 1809 | 414 | 1478 | 214 |
| Ei45 | V4 | 1561 | 347 | 1087 | 193 |
| PAC\_16 | V4 | 13702 | 5332 | 13073 | 4996 |
| PAC\_19 | V4 | 10657 | 3430 | 5294 | 2103 |
| PEC16\_1 | V4 | 925 | 169 | 915 | 160 |
| PEC16\_2 | V4 | 422 | 91 | 409 | 80 |
| SES11 | V4 | 781 | 297 | 94 | 46 |
| SES60 | V4 | 125 | 62 | 25 | 21 |
| Vil32 | V4 | 801 | 136 | 770 | 107 |
| Ei44\_1 | V9 | 2189 | 312 | 704 | 51 |
| Ei44\_2 | V9 | 2830 | 290 | 1035 | 50 |
| Ei45 | V9 | 1012 | 133 | 598 | 34 |
| PAC\_16 | V9 | 18864 | 1550 | 18135 | 1457 |
| PAC\_19 | V9 | 33748 | 1773 | 27530 | 1370 |
| PEC16\_1 | V9 | 1265 | 105 | 880 | 39 |
| PEC16\_2 | V9 | 1116 | 111 | 826 | 44 |
| SES11 | V9 | 2823 | 360 | 63 | 6 |
| SES60 | V9 | 1849 | 177 | 115 | 6 |
| Vil32 | V9 | 1514 | 87 | 1073 | 28 |

Note that a few amplicons are not assigned at all (less than 40%
identity with any reference sequences). It can be check using that
command:

```
for f in *[49]_dereplicated.fas ; do
    queries=$(grep -c "^>" $f)
    hits=$(grep "^>" $f | tr -d ">" | cut -d "_" -f 1 |
        while read l ; do
            grep -h -m 1 "^${l}" all_V*.results
        done | wc -l)
    echo $queries $hits
done
```

In the intersection analysis, the taxonomy-based filtering will be
done again, on-the-fly.

#### 3.3.5 Chimera checking with uchime

The probability to form identical chimeras in PCR replicates is low
but not null, that's why we had to test it.

```
# on my computer
mkdir -p uchime
# copy fasta files and modify the fasta header so usearch gets copy
# numbers (convert >readid_N to >readid;size=N)
for f in *_radiolaria.fas ; do
    sed -e 's/\_/;size=/' ${f} > ./uchime/${f}
done
# Invoque uchime (-chimeras and -nochimeras options do not work)
cd uchime/
TEMP=$(mktemp)
USEARCH="usearch7.0.1001_i86linux32"
for f in *_radiolaria.fas ; do
    if [[ -s "${f}" ]] ; then
        "${USEARCH}" -uchime_denovo "${f}" -uchimeout "${f/.fas/.uchime}"
        # List chimeras
        awk '{if ($NF == "Y") print $2}' "${f/.fas/.uchime}" > "${TEMP}"
        grep -A 1 -F -f "${TEMP}" "${f}" | sed -e '/^--$/d' -e 's/;size=/_/' > "${f/.fas/_uchime_rejected.fas}"
        # List non-chimeras
        awk '{if ($NF == "N") print $2}' "${f/.fas/.uchime}" > "${TEMP}"
        grep -A 1 -F -f "${TEMP}" "${f}" | sed -e '/^--$/d' -e 's/;size=/_/' > "${f/.fas/_uchime_validated.fas}"
    else
        cp "${f}" "${f/.fas/_uchime_validated.fas}"
    fi
done
rm -f "${TEMP}"
```

Uchime found no chimeras among our cross-validated amplicons.

#### 3.3.6 Common amplicons

Intersection of samples (or cross-validation) is based on the idea
that natural amplicons (representing sequences present in the initial
PCR mix) should be amplified and sequenced in both
replicates. Artificial amplicons, produced by random polymerase errors
(during PCR and sequencing) should have a low probability to appear in
both replicates. Therefore, keeping only amplicons common to both
replicates should remove a large fraction of the noise, while
preserving the taxonomic resolution (i.e. the capacity to detect all
the taxa initially present in the PCR mix).

We filter out non-radiolarians only after the intersection. It has a
noticeable effect on SES11–SES60 V9, where the intersection only
contains noise. The parents do contain reads assigned to the correct
taxa (Nassellarida, Lithomelissa): 63 reads in SES11-V9 and 109 in
SES60-V9, but none of these reads are shared between the two
samples. A look at an alignment explains why. All reads from SES60
have a "C" in position 62, where all reads from SES11 have a
"T". Consequently, the samples have no strictly identical reads in
common. These reads have been discarded by Acacia (low quality).

```
# Special analysis for SES11 ad SES60 V9
(grep "^>" SES60_V9_dereplicated.fas | sed -e 's/^>\(.*\)_.*/\1/' |
    while read l ; do
        grep $l all_V9.results
    done | grep Lithomelissa ;
 grep "^>" SES11_V9_dereplicated.fas | sed -e 's/^>\(.*\)_.*/\1/' |
 while read l ; do
     grep $l all_V9.results
 done | grep Lithomelissa) | cut -f 1 |
while read l ; do
    grep -A 1 $l all_V9.fas
done > Lithomelissa.fas

muscle -in Lithomelissa.fas -out Lithomelissa.fas_aln
seaview Lithomelissa.fas_aln
```

Intersection of samples, taxonomy based filtering and clustering

```
# List all replicate pairs
replicates="Ei44_1_V4 Ei44_2_V4
Ei44_1_V4 Ei45_V4
Ei44_2_V4 Ei45_V4
PEC16_1_V4 PEC16_2_V4
SES11_V4 SES60_V4
Ei44_1_V9 Ei44_2_V9
Ei44_1_V9 Ei45_V9
Ei44_2_V9 Ei45_V9
PEC16_1_V9 PEC16_2_V9
SES11_V9 SES60_V9"

# Extract common reads, remove non-radiolarians, clusterize and make a
# summary
while read replicates ; do
    sampleA=${replicates% *}
    sampleB=${replicates#* }
    region=${replicates##*_}
    
    # Extract common reads
    COMMON_READS=$(mktemp)
    RADIOLARIA_READS=$(mktemp)
    grep "^>" "${sampleA}_dereplicated.fas" "${sampleB}_dereplicated.fas" | cut -d '>' -f 2 | cut -d '_' -f1 | sort -d | uniq -d > "${COMMON_READS}"
    grep -A 1 -F -f "${COMMON_READS}" "${sampleA}_dereplicated.fas" | sed -e '/^--$/d' > "${sampleA}_vs_${sampleB}.fas"
    
    # Limit to Radiolaria only
    grep -F -f "${COMMON_READS}" "all_${region}.results" | sed -e '/^--$/d' | grep "Radiolaria" | cut -f 1 | sort -du > "${RADIOLARIA_READS}"
    grep -A 1 -F -f "${RADIOLARIA_READS}" "${sampleA}_dereplicated.fas" | sed -e '/^--$/d' > "${sampleA}_vs_${sampleB}_radiolaria.fas"
    rm "${COMMON_READS}" "${RADIOLARIA_READS}"
    
    # Clusterize
    USEARCH="usearch7.0.1001_i86linux32"
    f="${sampleA}_vs_${sampleB}_radiolaria.fas"
    TMP_USEARCH=$(mktemp)
    for THRESHOLD in {80..99} ; do
        OUTPUT_CLUSTERS="${f/.fas/.uclust_}${THRESHOLD}"
        "${USEARCH}" -cluster_smallmem -usersort "${f}" -id 0.${THRESHOLD} -uc "${TMP_USEARCH}"
        grep "^S" "${TMP_USEARCH}" | 
        while read a b c d e f g h i j ; do
            hits=$(grep "^H.*${i}$" "${TMP_USEARCH}" | cut -f 9 | tr "\n" " " | sed -e 's/\ $//')
            echo "${i} ${hits}"
        done > "${OUTPUT_CLUSTERS}"
    done
    rm "${TMP_USEARCH}"
    
    # Parse clustering results
    CLUSTER_COUNTS=$(wc -l ${sampleA}_vs_${sampleB}_radiolaria.uclust_?? | grep "uclust" | awk 'BEGIN {ORS="|"} {print $1} END {print "\n"}' | head -n 1)
    echo "|${sampleA}_vs_${sampleB}|${region}${CLUSTER_COUNTS}"
    rm ${sampleA}_vs_${sampleB}_radiolaria.uclust_??
done <<< "${replicates}" | grep "^|" > tmp


# Count reads and uniques
RADIOLARIANS=$(mktemp)
while read replicates ; do
    sampleA=${replicates% *}
    sampleB=${replicates#* }
    region=${replicates##*_}
    
    # Extract common reads (corrected: there was an error in the way
    # total and radiolaria_total were calculated (it should sum the
    # abundances of reads as indicated in both fasta files, not in the
    # STAMPA results)
    grep "^>" "${sampleA}_vs_${sampleB}.fas" | sed -e 's/^>\(.*\)_.*/\1/' |
    while read l ; do
        grep $l all_${region}.results
    done | grep "Radiolaria" > "${RADIOLARIANS}"
    total=$(grep "^>" "${sampleA}_vs_${sampleB}.fas" | while read l ; do read_id=${l%_*} ; grep "$read_id" "${sampleA}_dereplicated.fas" "${sampleB}_dereplicated.fas" ; done | awk 'BEGIN {FS="_"} {sum+=$NF} END {print sum}')
    uniques=$(grep -c "^>" "${sampleA}_vs_${sampleB}.fas")
    radiolaria_uniques=$(cut -f 1 "${RADIOLARIANS}" | sort -du | wc -l)
    radiolaria_total=$(cut -f 1 "${RADIOLARIANS}" | sort -du | while read l ; do read_id=${l%_*} ; grep ">${read_id}" "${sampleA}_dereplicated.fas" "${sampleB}_dereplicated.fas" ; done | awk 'BEGIN {FS="_"} {sum+=$NF} END {print sum}')
    echo "|${sampleA}_vs_${sampleB}|${total}|${uniques}|${radiolaria_total}|${radiolaria_uniques}|"

done <<< "${replicates}"
rm "${RADIOLARIANS}"
```

|  | All | All | Radiolaria | Radiolaria |  |  |  |  |  |  |  |  |  |  |  |  |  |  |  |  |  |  |  |  |
| --- | --- | --- | --- | --- | --- | --- | --- | --- | --- | --- | --- | --- | --- | --- | --- | --- | --- | --- | --- | --- | --- | --- | --- | --- |
| Intersected samples | Reads | Uniques | Reads | Uniques | 80 | 81 | 82 | 83 | 84 | 85 | 86 | 87 | 88 | 89 | 90 | 91 | 92 | 93 | 94 | 95 | 96 | 97 | 98 | 99 |
| Ei44\_1\_V4\_vs\_Ei44\_2\_V4 | 2196 | 79 | 1981 | 45 | 1 | 1 | 1 | 1 | 1 | 1 | 1 | 1 | 1 | 1 | 1 | 1 | 1 | 1 | 1 | 1 | 2 | 2 | 5 | 10 |
| Ei44\_1\_V4\_vs\_Ei45\_V4 | 1326 | 17 | 1294 | 10 | 1 | 1 | 1 | 1 | 1 | 1 | 1 | 1 | 1 | 1 | 1 | 1 | 1 | 1 | 1 | 1 | 1 | 1 | 1 | 2 |
| Ei44\_2\_V4\_vs\_Ei45\_V4 | 1174 | 10 | 1167 | 7 | 1 | 1 | 1 | 1 | 1 | 1 | 1 | 1 | 1 | 1 | 1 | 1 | 1 | 1 | 1 | 1 | 1 | 2 | 2 | 3 |
| PEC16\_1\_V4\_vs\_PEC16\_2\_V4 | 1131 | 32 | 1131 | 32 | 1 | 1 | 1 | 1 | 1 | 1 | 1 | 1 | 1 | 1 | 1 | 1 | 1 | 1 | 1 | 1 | 1 | 1 | 2 | 3 |
| SES11\_V4\_vs\_SES60\_V4 | 125 | 19 | 36 | 7 | 1 | 1 | 1 | 1 | 1 | 1 | 1 | 1 | 1 | 1 | 1 | 1 | 1 | 1 | 1 | 1 | 1 | 1 | 1 | 2 |
| Ei44\_1\_V9\_vs\_Ei44\_2\_V9 | 4277 | 62 | 1633 | 14 | 1 | 1 | 1 | 1 | 1 | 2 | 2 | 2 | 2 | 2 | 2 | 2 | 2 | 2 | 2 | 2 | 2 | 3 | 5 | 6 |
| Ei44\_1\_V9\_vs\_Ei45\_V9 | 2552 | 37 | 1206 | 9 | 1 | 1 | 1 | 1 | 1 | 2 | 2 | 2 | 2 | 2 | 2 | 2 | 2 | 2 | 2 | 2 | 2 | 2 | 4 | 4 |
| Ei44\_2\_V9\_vs\_Ei45\_V9 | 3309 | 49 | 1543 | 11 | 1 | 1 | 1 | 1 | 1 | 2 | 2 | 2 | 2 | 2 | 2 | 2 | 2 | 2 | 2 | 2 | 2 | 2 | 4 | 5 |
| PEC16\_1\_V9\_vs\_PEC16\_2\_V9 | 2162 | 35 | 1635 | 14 | 1 | 1 | 1 | 1 | 1 | 2 | 2 | 2 | 2 | 2 | 2 | 2 | 2 | 2 | 3 | 3 | 3 | 3 | 4 | 4 |
| SES11\_V9\_vs\_SES60\_V9 | 2305 | 23 | 0 | 0 | 0 | 0 | 0 | 0 | 0 | 0 | 0 | 0 | 0 | 0 | 0 | 0 | 0 | 0 | 0 | 0 | 0 | 0 | 0 | 0 |

For each intersection, the sum of reads is of course lesser than the
sum of the reads in parent samples, but still important. The number of
unique reads is more than one order of magnitude smaller than the
number of unique reads in parent samples. It shows the cleaning
efficiency of replication intersection.

We obtain slightly more OTUs using PWM (MOODS) than using regular
expressions. In theory, we expect identical results: after the PCR
process, a majority of reads are supposed to contain the primer used
to amplify them (replacement). But empirical results show that it is
possible to obtain large quantities of reads with primer regions
different from the primers used to perform the amplification.

In summary, it is important to allow a certain flexibility during the
search for primer sequences (1 or 2 differences, depending on the
primers length and complexity).

Using PWM allowed us to capture reads assigned to Radiolaria for the
samples SES11 and SES60 (region V4) where regular expressions or
Acacia yield no reads. For the V9 region, none of the different
methods were able to capture reads assigned to Radiolaria (only
contaminants). From Acacia results, we deduce that the reads
intersection captures for SES11–SES60 V4 have some low quality
segments, but the intersection confirms the reality of these reads.

With the intersection method most low abundant reads (the so-called
rare biosphere) is discarded, without any apparent lost of resolution:
the targeted taxa are captured, alongside expected contaminants:
fungi, mammal, known marine eucaryots and bacteria (with the V9
primers).

#### 3.3.7 Uncommon amplicons

We keep radiolarian amplicons that are not common to both replicates
(exclusion instead of inclusion) to assess the level of OTU richness
represented by these excluded amplicons.

```
### Intersection of samples, taxonomy based filtering and clustering

# List all replicate pairs
replicates="Ei44_1_V4 Ei44_2_V4
Ei44_1_V4 Ei45_V4
Ei44_2_V4 Ei45_V4
PEC16_1_V4 PEC16_2_V4
SES11_V4 SES60_V4
Ei44_1_V9 Ei44_2_V9
Ei44_1_V9 Ei45_V9
Ei44_2_V9 Ei45_V9
PEC16_1_V9 PEC16_2_V9
SES11_V9 SES60_V9"

rm tmp*

# Extract non-common reads, remove non-radiolarians, clusterize and
# make a summary
while read replicates ; do
    sampleA=${replicates% *}
    sampleB=${replicates#* }
    region=${replicates##*_}
    
    # Extract common reads
    NON_COMMON_READS=$(mktemp)
    RADIOLARIA_READS=$(mktemp)
    grep -h "^>" "${sampleA}_dereplicated.fas" "${sampleB}_dereplicated.fas" | tr -d ">" | cut -d '_' -f 1 | sort -d | uniq -u > "${NON_COMMON_READS}"
    grep -h -A 1 -F -f "${NON_COMMON_READS}" "${sampleA}_dereplicated.fas" "${sampleB}_dereplicated.fas" | sed -e '/^--$/d' > "${sampleA}_vs_${sampleB}_exclusion.fas"
    
    # Limit to Radiolaria only
    grep -F -f "${NON_COMMON_READS}" "all_${region}.results" | sed -e '/^--$/d' | grep "Radiolaria" | cut -f 1 | sort -du > "${RADIOLARIA_READS}"
    grep -h -A 1 -F -f "${RADIOLARIA_READS}" "${sampleA}_dereplicated.fas" "${sampleB}_dereplicated.fas" | sed -e '/^--$/d' > "${sampleA}_vs_${sampleB}_radiolaria_exclusion.fas"
    rm "${NON_COMMON_READS}" "${RADIOLARIA_READS}"
    
    # Clusterize
    USEARCH="usearch7.0.1001_i86linux32"
    f="${sampleA}_vs_${sampleB}_radiolaria_exclusion.fas"
    TMP_USEARCH=$(mktemp)
    for THRESHOLD in {80..99} ; do
        OUTPUT_CLUSTERS="${f/.fas/.uclust_}${THRESHOLD}"
        "${USEARCH}" -cluster_smallmem -usersort "${f}" -id 0.${THRESHOLD} -uc "${TMP_USEARCH}"
        grep "^S" "${TMP_USEARCH}" | 
        while read a b c d e f g h i j ; do
            hits=$(grep "^H.*${i}$" "${TMP_USEARCH}" | cut -f 9 | tr "\n" " " | sed -e 's/\ $//')
            echo "${i} ${hits}"
        done > "${OUTPUT_CLUSTERS}"
    done
    rm "${TMP_USEARCH}"
    
    # Parse clustering results (all levels, and 98%)
    CLUSTER_COUNTS=$(wc -l ${sampleA}_vs_${sampleB}_radiolaria_exclusion.uclust_* | grep "uclust" | awk 'BEGIN {ORS="|"} {print $1} END {print "\n"}' | head -n 1)
    echo "|${sampleA}_vs_${sampleB}|${region}${CLUSTER_COUNTS}"
    # OTU sizes at 98%
    echo ${sampleA}_vs_${sampleB} >> tmp2
    while read l ; do
        tr " " "\n" <<< "${l}" | awk 'BEGIN {FS = "_"} {sum+=$2} END {print sum}'
    done < ${sampleA}_vs_${sampleB}_radiolaria_exclusion.uclust_98 | sort -nr | uniq -c >> tmp2
    echo >> tmp2
    rm ${sampleA}_vs_${sampleB}_radiolaria_exclusion.uclust_*
done <<< "${replicates}" | grep "^|" > tmp

# Count reads and uniques
RADIOLARIANS=$(mktemp)
while read replicates ; do
    sampleA=${replicates% *}
    sampleB=${replicates#* }
    region=${replicates##*_}
    
    # Extract uncommon reads
    grep "^>" "${sampleA}_vs_${sampleB}_exclusion.fas" | sed -e 's/^>\(.*\)_.*/\1/' |
    while read l ; do
        grep $l all_${region}.results
    done | grep "Radiolaria" > "${RADIOLARIANS}"
    total=$(grep "^>" "${sampleA}_vs_${sampleB}_exclusion.fas" | while read l ; do read_id=${l%_*} ; grep -h "$read_id" "${sampleA}_dereplicated.fas" "${sampleB}_dereplicated.fas" ; done | awk 'BEGIN {FS="_"} {sum+=$NF} END {print sum}')
    uniques=$(grep -c "^>" "${sampleA}_vs_${sampleB}_exclusion.fas")
    radiolaria_uniques=$(cut -f 1 "${RADIOLARIANS}" | sort -du | wc -l)
    radiolaria_total=$(cut -f 1 "${RADIOLARIANS}" | sort -du | while read l ; do read_id=${l%_*} ; grep -h ">${read_id}" "${sampleA}_dereplicated.fas" "${sampleB}_dereplicated.fas" ; done | awk 'BEGIN {FS="_"} {sum+=$NF} END {print sum}')
    echo "|${sampleA}_vs_${sampleB}|${total}|${uniques}|${radiolaria_total}|${radiolaria_uniques}|"
done <<< "${replicates}"
rm "${RADIOLARIANS}"
```

When removing un-common reads, we mostly remove micro-variants less
abundant thant natural amplicons. Consequently, when we clusterize
these uncommon amplicons, it is normal to find a "shadow" of the OTUs
visible in the intersection. It is also normal to find a higher number
of OTUs, and a higher number of singletons.

|  | All | All | Radiolaria | Radiolaria |  |  |  |  |  |  |  |  |  |  |  |  |  |  |  |  |  |  |  |  |
| --- | --- | --- | --- | --- | --- | --- | --- | --- | --- | --- | --- | --- | --- | --- | --- | --- | --- | --- | --- | --- | --- | --- | --- | --- |
| Exclusion of samples | Reads | Uniques | Reads | Uniques | 80 | 81 | 82 | 83 | 84 | 85 | 86 | 87 | 88 | 89 | 90 | 91 | 92 | 93 | 94 | 95 | 96 | 97 | 98 | 99 |
| Ei44\_1\_V4\_vs\_Ei44\_2\_V4 | 1596 | 709 | 1208 | 385 | 1 | 2 | 2 | 2 | 2 | 3 | 5 | 5 | 6 | 6 | 6 | 6 | 6 | 8 | 9 | 10 | 14 | 18 | 28 | 90 |
| Ei44\_1\_V4\_vs\_Ei45\_V4 | 2218 | 766 | 1504 | 434 | 2 | 3 | 3 | 3 | 3 | 5 | 5 | 6 | 7 | 7 | 7 | 7 | 7 | 9 | 10 | 10 | 13 | 17 | 24 | 79 |
| Ei44\_2\_V4\_vs\_Ei45\_V4 | 2196 | 741 | 1398 | 393 | 2 | 2 | 2 | 2 | 2 | 2 | 2 | 2 | 2 | 2 | 2 | 2 | 2 | 2 | 2 | 4 | 6 | 11 | 18 | 75 |
| PEC16\_1\_V4\_vs\_PEC16\_2\_V4 | 216 | 196 | 193 | 176 | 1 | 1 | 1 | 1 | 1 | 1 | 1 | 1 | 1 | 1 | 1 | 1 | 1 | 1 | 1 | 1 | 2 | 5 | 9 | 37 |
| SES11\_V4\_vs\_SES60\_V4 | 781 | 321 | 83 | 53 | 2 | 2 | 2 | 2 | 2 | 2 | 2 | 2 | 2 | 2 | 3 | 3 | 3 | 3 | 4 | 4 | 4 | 6 | 7 | 16 |
| Ei44\_1\_V9\_vs\_Ei44\_2\_V9 | 742 | 478 | 106 | 73 | 2 | 2 | 2 | 3 | 3 | 3 | 3 | 3 | 3 | 3 | 4 | 4 | 4 | 6 | 8 | 10 | 13 | 17 | 25 | 52 |
| Ei44\_1\_V9\_vs\_Ei45\_V9 | 649 | 371 | 96 | 67 | 1 | 1 | 1 | 1 | 1 | 1 | 1 | 2 | 2 | 2 | 3 | 3 | 5 | 6 | 7 | 10 | 11 | 13 | 22 | 54 |
| Ei44\_2\_V9\_vs\_Ei45\_V9 | 533 | 325 | 90 | 62 | 3 | 3 | 3 | 3 | 3 | 3 | 4 | 4 | 4 | 4 | 4 | 4 | 6 | 8 | 9 | 13 | 15 | 18 | 28 | 47 |
| PEC16\_1\_V9\_vs\_PEC16\_2\_V9 | 219 | 146 | 71 | 55 | 3 | 3 | 3 | 3 | 3 | 3 | 3 | 4 | 4 | 4 | 4 | 4 | 4 | 5 | 5 | 6 | 7 | 10 | 15 | 41 |
| SES11\_V9\_vs\_SES60\_V9 | 2367 | 491 | 178 | 12 | 2 | 2 | 2 | 2 | 2 | 2 | 2 | 2 | 2 | 2 | 2 | 2 | 2 | 2 | 2 | 2 | 2 | 4 | 4 | 5 |

#### 3.3.8 Triplicate: the case of Ei44 and Ei45

We have a technical replicate (Ei44), but we also have a natural
duplicate (Ei45). Ei45 and Ei44 are assigned to the same
morpho-species. We apply the same intersection and exclusion method to
the three samples.

- Exclusion   

  ```
  ### Intersection of samples, taxonomy based filtering and clustering

  # List all replicate pairs
  triplicates="Ei44_1_V4 Ei44_2_V4 Ei45_V4
  Ei44_1_V9 Ei44_2_V9 Ei45_V9"

  rm tmp*

  # Extract non-common reads, remove non-radiolarians, clusterize and
  # make a summary
  while read triplicate ; do
      sampleA=$(cut -d " " -f 1 <<< "${triplicate}")
      sampleB=$(cut -d " " -f 2 <<< "${triplicate}")
      sampleC=$(cut -d " " -f 3 <<< "${triplicate}")
      region=${triplicate##*_}

      exclusion="${sampleA}_vs_${sampleB}_vs_${sampleC}_exclusion.fas"
      exclusion_radiolaria="${sampleA}_vs_${sampleB}_vs_${sampleC}_radiolaria_exclusion.fas"
      all_samples="${sampleA}_dereplicated.fas ${sampleB}_dereplicated.fas ${sampleC}_dereplicated.fas"

      # Extract common reads
      NON_COMMON_READS=$(mktemp)
      RADIOLARIA_READS=$(mktemp)
      grep -h "^>" ${all_samples} | tr -d ">" | cut -d '_' -f 1 | sort -d | uniq -c | awk '$1 < 3 {print $2}' > "${NON_COMMON_READS}"
      grep -h -A 1 -F -f "${NON_COMMON_READS}" ${all_samples} | sed -e '/^--$/d' > "${exclusion}"
      
      # Limit to Radiolaria only
      grep -F -f "${NON_COMMON_READS}" "all_${region}.results" | sed -e '/^--$/d' | grep "Radiolaria" | cut -f 1 | sort -du > "${RADIOLARIA_READS}"
      grep -h -A 1 -F -f "${RADIOLARIA_READS}" "${exclusion}" | sed -e '/^--$/d' > "${sampleA}_vs_${sampleB}_vs_${sampleC}_radiolaria_exclusion.fas"
      
      # Clusterize
      USEARCH="usearch7.0.1001_i86linux32"
      f="${sampleA}_vs_${sampleB}_vs_${sampleC}_radiolaria_exclusion.fas"
      TMP_USEARCH=$(mktemp)
      for THRESHOLD in {80..99} ; do
          OUTPUT_CLUSTERS="${f/.fas/.uclust_}${THRESHOLD}"
          "${USEARCH}" -cluster_smallmem -usersort "${f}" -id 0.${THRESHOLD} -uc "${TMP_USEARCH}" &> /dev/null
          grep "^S" "${TMP_USEARCH}" | 
          while read a b c d e f g h i j ; do
              hits=$(grep "^H.*${i}$" "${TMP_USEARCH}" | cut -f 9 | tr "\n" " " | sed -e 's/\ $//')
              echo "${i} ${hits}"
          done > "${OUTPUT_CLUSTERS}"
      done
      rm "${TMP_USEARCH}"
      
      # Parse clustering results (all levels, and 98%)
      total=$(grep "^>" "${exclusion}" |
          while read l ; do
              read_id=${l%_*}
              grep -h "$read_id" ${all_samples}
          done | awk 'BEGIN {FS="_"} {sum+=$NF} END {print sum}')
      uniques=$(grep -c "^>" "${exclusion}")
      radiolaria_uniques=$(wc -l < "${RADIOLARIA_READS}")
      radiolaria_total=$(grep -h -F -f "${RADIOLARIA_READS}" ${all_samples} | awk 'BEGIN {FS="_"} {sum += $2} END {print sum}')
      CLUSTER_COUNTS=$(wc -l ${sampleA}_vs_${sampleB}_vs_${sampleC}_radiolaria_exclusion.uclust_* | grep "uclust" | awk 'BEGIN {ORS="|"} {print $1} END {print "\n"}' | head -n 1)
      echo "|${sampleA}_vs_${sampleB}_vs_${sampleC}|${region}|${total}|${uniques}|${radiolaria_total}|${radiolaria_uniques}|${CLUSTER_COUNTS}"

      # OTU sizes at 98%
      AMPLICONS_IN_OTU=$(mktemp)
      echo ${sampleA}_vs_${sampleB}_vs_${sampleC} >> tmp
      while read l ; do
          tr " " "\n" <<< "${l}" | cut -d "_" -f 1 > "${AMPLICONS_IN_OTU}"
          grep -h -F -f "${AMPLICONS_IN_OTU}" ${all_samples} | awk 'BEGIN {FS = "_"} {sum+=$2} END {print sum}'
      done < ${sampleA}_vs_${sampleB}_vs_${sampleC}_radiolaria_exclusion.uclust_98 | sort -nr | uniq -c >> tmp
      echo >> tmp

      # Cleaning
      rm ${sampleA}_vs_${sampleB}_vs_${sampleC}_radiolaria_exclusion.uclust_*
      rm "${AMPLICONS_IN_OTU}" "${NON_COMMON_READS}" "${RADIOLARIA_READS}"
  done <<< "${triplicates}"
  cat tmp
  ```

  |  | All | All | Radiolaria | Radiolaria |  |  |  |  |  |  |  |  |  |  |  |  |  |  |  |  |  |  |  |  |
  | --- | --- | --- | --- | --- | --- | --- | --- | --- | --- | --- | --- | --- | --- | --- | --- | --- | --- | --- | --- | --- | --- | --- | --- | --- |
  | Exclusion of samples | Reads | Uniques | Reads | Uniques | 80 | 81 | 82 | 83 | 84 | 85 | 86 | 87 | 88 | 89 | 90 | 91 | 92 | 93 | 94 | 95 | 96 | 97 | 98 | 99 |
  | Ei44\_1\_V4\_vs\_Ei44\_2\_V4\_vs\_Ei45\_V4 | 6299 | 1199 | 3050 | 606 | 2 | 3 | 3 | 3 | 3 | 5 | 5 | 6 | 7 | 7 | 7 | 7 | 7 | 9 | 10 | 11 | 15 | 20 | 30 | 113 |
  | Ei44\_1\_V9\_vs\_Ei44\_2\_V9\_vs\_Ei45\_V9 | 1391 | 642 | 176 | 101 | 2 | 2 | 2 | 3 | 3 | 3 | 4 | 4 | 4 | 4 | 4 | 4 | 5 | 8 | 10 | 14 | 17 | 21 | 32 | 71 |
- Intersection   

  ```
  ### Intersection of samples, taxonomy based filtering and clustering

  # List all replicate triplets
  triplicates="Ei44_1_V4 Ei44_2_V4 Ei45_V4
  Ei44_1_V9 Ei44_2_V9 Ei45_V9"

  rm tmp*

  # Extract non-common reads, remove non-radiolarians, clusterize and
  # make a summary
  while read triplicate ; do
      sampleA=$(cut -d " " -f 1 <<< "${triplicate}")
      sampleB=$(cut -d " " -f 2 <<< "${triplicate}")
      sampleC=$(cut -d " " -f 3 <<< "${triplicate}")
      region=${triplicate##*_}

      intersection="${sampleA}_vs_${sampleB}_vs_${sampleC}.fas"
      intersection_radiolaria="${sampleA}_vs_${sampleB}_vs_${sampleC}_radiolaria.fas"
      all_samples="${sampleA}_dereplicated.fas ${sampleB}_dereplicated.fas ${sampleC}_dereplicated.fas"

      # Extract common reads
      COMMON_READS=$(mktemp)
      RADIOLARIA_READS=$(mktemp)
      # Change awk $1 == 3, $1 < 3 to select common or non-common reads
      grep -h "^>" ${all_samples} | tr -d ">" | cut -d '_' -f 1 | sort -d | uniq -c | awk '$1 == 3 {print $2}' > "${COMMON_READS}"
      grep -h -A 1 -F -f "${COMMON_READS}" ${all_samples} | sed -e '/^--$/d' > "${intersection}"
      
      # Limit to Radiolaria only
      grep -F -f "${COMMON_READS}" "all_${region}.results" | sed -e '/^--$/d' | grep "Radiolaria" | cut -f 1 | sort -du > "${RADIOLARIA_READS}" 
      grep -h -A 1 -F -f "${RADIOLARIA_READS}" "${sampleA}_dereplicated.fas" | sed -e '/^--$/d' > "${sampleA}_vs_${sampleB}_vs_${sampleC}_radiolaria.fas"
      
      # Clusterize
      USEARCH="usearch7.0.1001_i86linux32"
      f="${sampleA}_vs_${sampleB}_vs_${sampleC}_radiolaria.fas"
      TMP_USEARCH=$(mktemp)
      for THRESHOLD in {80..99} ; do
          OUTPUT_CLUSTERS="${f/.fas/.uclust_}${THRESHOLD}"
          "${USEARCH}" -cluster_smallmem -usersort "${f}" -id 0.${THRESHOLD} -uc "${TMP_USEARCH}" &> /dev/null
          grep "^S" "${TMP_USEARCH}" |
          while read a b c d e f g h i j ; do
              hits=$(grep "^H.*${i}$" "${TMP_USEARCH}" | cut -f 9 | tr "\n" " " | sed -e 's/\ $//')
              echo "${i} ${hits}"
          done > "${OUTPUT_CLUSTERS}"
      done
      rm "${TMP_USEARCH}"
      
      # Parse clustering results (all levels, and 98%)
      total=$(grep "^>" "${intersection}" |
          while read l ; do
              read_id=${l%_*}
              grep -h "$read_id" ${all_samples}
          done | awk 'BEGIN {FS="_"} {sum+=$NF} END {print sum}')
      uniques=$(grep -c "^>" "${intersection}")
      radiolaria_uniques=$(wc -l < "${RADIOLARIA_READS}")
      radiolaria_total=$(grep -h -F -f "${RADIOLARIA_READS}" ${all_samples} | awk 'BEGIN {FS="_"} {sum += $2} END {print sum}')
      CLUSTER_COUNTS=$(wc -l ${sampleA}_vs_${sampleB}_vs_${sampleC}_radiolaria.uclust_* | grep "uclust" | awk 'BEGIN {ORS="|"} {print $1} END {print "\n"}' | head -n 1)
      echo "|${sampleA}_vs_${sampleB}_vs_${sampleC}|${region}|${total}|${uniques}|${radiolaria_total}|${radiolaria_uniques}|${CLUSTER_COUNTS}"

      # OTU sizes at 98%
      AMPLICONS_IN_OTU=$(mktemp)
      echo ${sampleA}_vs_${sampleB}_vs_${sampleC} >> tmp
      while read l ; do
          tr " " "\n" <<< "${l}" | cut -d "_" -f 1 > "${AMPLICONS_IN_OTU}"
          grep -h -F -f "${AMPLICONS_IN_OTU}" ${all_samples} | awk 'BEGIN {FS = "_"} {sum+=$2} END {print sum}'
      done < ${sampleA}_vs_${sampleB}_vs_${sampleC}_radiolaria.uclust_98 | sort -nr | uniq -c >> tmp
      echo >> tmp

      # Cleaning
      rm ${sampleA}_vs_${sampleB}_vs_${sampleC}_radiolaria.uclust_*
      rm "${AMPLICONS_IN_OTU}" "${COMMON_READS}" "${RADIOLARIA_READS}"
  done <<< "${triplicates}"
  cat tmp
  ```

  |  |  | All | All | Radiolaria | Radiolaria |  |  |  |  |  |  |  |  |  |  |  |  |  |  |  |  |  |  |  |  |
  | --- | --- | --- | --- | --- | --- | --- | --- | --- | --- | --- | --- | --- | --- | --- | --- | --- | --- | --- | --- | --- | --- | --- | --- | --- | --- |
  | Intersection of samples | region | Reads | Uniques | Reads | Uniques | 80 | 81 | 82 | 83 | 84 | 85 | 86 | 87 | 88 | 89 | 90 | 91 | 92 | 93 | 94 | 95 | 96 | 97 | 98 | 99 |
  | Ei44\_1\_V4\_vs\_Ei44\_2\_V4\_vs\_Ei45\_V4 | V4 | 3750 | 15 | 1226 | 2 | 1 | 1 | 1 | 1 | 1 | 1 | 1 | 1 | 1 | 1 | 1 | 1 | 1 | 1 | 1 | 1 | 1 | 1 | 1 | 1 |
  | Ei44\_1\_V9\_vs\_Ei44\_2\_V9\_vs\_Ei45\_V9 | V9 | 14778 | 93 | 2161 | 7 | 1 | 1 | 1 | 1 | 1 | 2 | 2 | 2 | 2 | 2 | 2 | 2 | 2 | 2 | 2 | 2 | 2 | 2 | 4 | 4 |

  Note that there is only 2 common radiolaria amplicons for Ei44-Ei45
  triplicate. The two amplicons differ by only one insertion in the
  longest homopolymer stretch (5 or 6 As) found in this region. It could
  be natural or artificial, difficult to tell, but the true sequence is
  probably the most abundant of the two. Let's check:

  | amplicon | length | total | Ei44\_1 | Ei44\_2 | Ei45 |
  | --- | --- | --- | --- | --- | --- |
  | cb8beb27a8c38b7044dd2e1e0b9a64ec | 372 | 1000 | 136 | 715 | 149 |
  | 6f63a8f394a265e3cbeddfb335d68b37 | 373 | 226 | 2 | 1 | 223 |

  Interestingly, the longest version is the most abundant in Ei45, but
  almost non-existant in Ei44.

### 3.4 Linkage Method

The linkage method was customized by Eriko Sasaki for V4 and V9
barcodes produced with 454 pyrosequencing. Aligned amplicon sequences
were analyzed with variable-sized sliding windows without
overlaps. When distance between the segregating (heterozygous) sites
was less than 5 bp, the sites were considered to be contained in the
same window. Sequences within a window was treated as partial
patterns. Singletons of partial pattern were treated as missing values
because there was no reproducibility for the pattern in the
sequences. After excluding non-reproducible partial patterns,
frequency of whole sequencing patterns (amplicons) was
counted. Missing values were treated as "wild card" and the amplicons
that contain "wild card" were counted as a part of amplicon that
consisted of reproducible patterns.

## 4 Final fasta files

We provide the amplicons obtained with the denoising pipeline (Acacia,
Uchime). Only amplicons assigned to Radiolaria and containing both
primers (forward and reverse) were kept.

```
# Ei44_1_V4_radiolaria.fas
>2aa596477e481a5c24ec1af501ea447585efe383_156
AGCTCCAATAGCGTATACTAATGTTGTTGCAGTTAAAAGCTCGTAGTCGGATTTCAGTAGGCTCAATTAAGTCTCTATTCTGAGTTCTTTATGGGCCTGCTTCTTTGACAGAAACTTCTATGTTATTCATTTAGCGTGGGTAGCGACTGTCTCTTTTACTTTGAGAAAATTAGAGTGTTCAAAGCAGGTATTCGCCTGAATATTACTCTTGGAATAATGCTATAAGACTTTGGTTCTAATGTATTGGTGATTGGGACCAGAGTAATGATTGATAGGGACGGTTGGGGTCTTACGTACTGCAAAGCGAGAGGTGAAATTCTTGGACCTTTGTATGACGAACAACTGCGAAAGCATTGGACTAGGACGTTCCCG
>d3be605345dc3e4f444a89c60334fdde85b82a7b_17
AGCTCCAATAGCGTATACTAATGTTGTTGCAGTTAAAAGACTCGTAGTCGGATTTCAGTAGGCTCAATTAAGTCTCTATTCTGAGTTCTTTATGGGCCTGCTTCTTTGACAGAAACTTCTATGTTATTCATTTAGCGTGGGTAGCGACTGTCTCTTTTACTTTGAGAAAATTAGAGTGTTCAAAGCAGGTATTCGCCTGAATATTACTCTTGGAATAATGCTATAAGACTTTGGTTCTAATGTATTGGTGATTGGGACCAGAGTAATGATTGATAGGGACGGTTGGGGTCTTACGTACTGCAAAGCGAGAGGTGAAATTCTTGGACCTTTGTATGACGAACAACTGCGAAAGCATTGGACTAGGACGTTCCCG
>fcdfafc671782f35d589f9b174458f6091155246_2
AGCTCCAATAGCGTATACTAATGTTGTTGCAGTTAAAAGCTCGTAGTCGGATTTCAGTAGGCTCAGTTAAGTCTCACTTATGAGTTCTTTATGGGCCTGCTTCTTTGACAGAAACTTCTATGTTATTCATTTAGCGTGGGTAGCGACTGTCTCTTTTACTTTGAGAAAATTAGAGTGTTCAAAGCAGGTATTCGCCTGAATATTACTCTTGGAATAATGCTATAAGACTTTGGTTCTAATGTATTGGTGATTGGGACCAGAGTAATGATTGATAGGGACGGTTGGGGTCTTACGTACTGCAAAGCGAGAGGTGAAATTCTTGGACCTTTGTATGACGAACAACTGCGAAAGCATTGGACTAGGACGTTCCCG
>0a0e97125a9b27da574e665e6dec565a8b004af0_1
AGCTCCAATAGCGTATACTAATGTTGTTGCAGTTAAAAGCTCGTAGTCGGATTTCAGTAGGCTCAATTAAGTCTCTATTCTGAGTTCTTTATGGGCCTGCTTCTTTGACAGAAACTTCTATGTTATTCATTTAGCGTGGGTAGCGACTGTCTCTTTTACTTTGGAGAAAATTAGAGTGTTCAAAGCAGGTATTCGCCTGAATATTACTCTTGGAATAATGCTATAAGACTTTGGTTCTAATGTATTGGTGATTGGGACCAGAGTAATGATTGATAGGGACGGTTGGGGTCTTACGTACTGCAAAGCGAGAGGTGAAATTCTTGGACCTTTGTATGACGAACAACTGCGAAAGCATTGGACTAGGACGTTCCCG
>25536ca13d9c2a6579442751cbff68305141f4ef_1
AGCTCCAATAGCGTATACTAATGTTGTTGCAGTTAAAAGCTCGTAGTCGGATTTCAGTAGGCTCAATTAAGTCTCTATTCTGAGTTCTTTATGGGCCTGCTTCTTTGACAGAAACTTCTATGTTATTCATTTAGCGTGGGTAGCGACTGTCTCTTTTACTTTGAGAAAATTAGAGTGTTCAAAGCAGGTATTCGCCTGAATATTACTCTTGGAATAATGCTATAAGACTTTGGTTCTAATGTATTGGTGATTGGGACCAGAGTAATGATTGATAGGGACGGTTGGGGTCTTACGTACTGCAAAGCGAGAGGCGAAATTCTTGGACCTTTGTATGACGAACAACTGCGAAAGCATTGGACTAGGACGTTCCCG
>541ce020752d514359919e6856d49c08eb557d32_1
AGCTCCAATAGCGTATATTAAAGTTGCTGCAGTTAAAAGCTCGTAGTCGGATTTCAGTAGGCTCAATTAAGTCTCTATTCTGAGTTCTTTATGGGCCTGCTTCTTTGACAGAAACTTCTATGTTATTCATTTAGCGTGGGTAGCGACTGTCTCTTTTACTTTGAGAAAATTAGAGTGTTCAAAGCAGGTATTCGCCTGAATATTACTCTTGGAATAATGCTATAAGACTTTGGTTCTAATGTATTGGTGATTGGGACCAGAGTAATGATTGATAGGGACGGTTGGGGTCTTACGTACTGCAAAGCGAGAGGTGAAATTCTTGGACCTTTGTATGACGAACAACTGCGAAAGCATTGGACTAGGACGTTCCCG
>555f1f75d1dbf99efae4a22845a96303661cacab_1
AGCTCCAATAGCGTATACTAATGTTGTTGCAGTTAAAAGCTCGTAGTCGGATTTCAGTAAGCTCAGTTAAGTCTCTATTCTGAGTTCTTTATGGGCCTGTTTCTTTGACAGAAACTTCTATGTTATTCATTTAGCGTGGGTAGCGACTGTCTCTTTTACTTTGAGAAAATTAGAGTGTTCAAAGCAGGTATTCGCCTGAATATTACTCTTGGAATAATGCTATAAGACTTTGGTTCTAATGTATTGGTGATTGGGACCAGAGTAATGATTGATAGGGACGGTTGGGGTCTTACGTACTGCAAAGCGAGAGGTGAAATTCTTGGACCTTTGTATGACGAACAACCGCGAAGCATTGGACTAGGACGTTCCCG
>5eb443188af8778a8d3eab2eacd14dcc16de9cd1_1
AGCTCCAATAGCGTATACTAATGTTGTTGCAGTTAAAAGACTCGTAGTCGGATTTCAGTAGGCTCAATTAAGTCTCTATTCTGAGTTCTTTATGGGCCTGCTTCTTTGACAGAAACTTCTATGTTATTCATTTAGCGTGGGTAGCGACTGTCTCTTTTACTTTGAGAAAATTAGAGTGTTCAAAGCAGGTATTCGCCTGAATATTACTCTTGGAATAATGCTATAAGACTTTGGTTCTAATGTATTGGTGATTGGGACCAGAGTAATGATTGATAGGGACGGTTGGGGTCTTACGTACTACAAAGCGAGAGGTGAAATTCTTGGACCTTTGTATGACGAACAACTGCGAAAGCATTGGACTAGGACGTTCCCG
>81274e3e25af167c759cafa108e0c8ad09d0efba_1
AGCTCCAATAGCGTATACTAATGTTGTTGCAGTTAAAAGACTCGTAGTCGGATTTCAGTAGGCTCAATTAAGTCTCTATTCTGAGTTCTTTATGGGCCTGCTTCTTTGACAGAAACTTCTATGTTATTCATTTAGCGTGGGTAGCGACTGTCTCTTTTACTTTGAGAAAATTAGAGTGTTCAAAACAGGTATTCGCCTGAATATTACTCTTGGAATAATGCTATAAGACTTTGGTTCTAATGTATTGGTGATTGGGACCAGAGTAATGATTGATAGGGACGGTTGGGGTCTTACGTACTGCAAAGCGAGAGGTGAAATTCTTGGACCTTTGTATGACGAACAACTGCGAAAGCATTGGACTAGGACGTTCCCG
>8ec3672a95c9413bbefd2908e63f37096c232bfd_1
AGCTCCAATAGCGTATACTAATGTTGTTGCAGTTAAAAACTACGTAGTTGGATTTCAGTAGGTTCAATTAAGTCTCTATTCTGAGTTCTTTATGGTCCTGTTGTTTTGACAGAAACTTCTATGTTATTCATTTAGCGTGGGTAGCGACTGTCTCTTTTACTTTGAGAAAATTAAAGTGTTCAAAGCAGGTATTCGCCTGAATATTACTTTTGGAATAATGCTATAAGACTTTGGTTCTAATGTATTTGTGATTGGGGACCAGAGTAATGATTGATAGGGACGGTTGAGGTCTTACGTACTGCAAAACGAGAGGTGAAATTCTTGGACCTTTGTATGACGAACAACTGCGAAAGCATTGGACTAGGACGTTCCCG
>90f5941b5097b4e3ac8da6dd7d14a6a979530744_1
AGCTCCAATAGCGTATACTAATGTTGTTGCAGTTAAAAGCTCGTAGTCGGATTTCAGTAGGCTCAATTAAGTCTCTATTCTGAGTTCTTTATGGGCCTGTTTCTTTGACAGAAACTTCTATGTTATTCATTTAACGTGGGTAGCGACTGTCTCTTTTACTTTGAGAAAATTAGAGTGTTCAAAGCAGGTATTCGCCTGAATATTACTCTTGGAATAATGCTATAAGACTTTGGCTCTAATGTATTGGTGATTGGGACCAGAGTAATGATTGATAGGGACGGTTGGGGTCTTACGTACTGCACAGCGAGAGGTGAAATTCTTGGACCTTTGTATGACGAACAACTGCGAAAACATTGGACTAGGACGTTCCCG
>ad92594c7f966620d8773ea92f2a440513d90fff_1
AGCTCCAATAGCGTATACTAATGTTGTTGCAGTTAAAAGCTCGTAGTCGGATTTCAGTAGGCTCAATTAAGTCTCTATTCTGAGTTCTTTATGGGCCTGCTTCTTTGACAGAAACTTCTATGTTATTCATTTAGCGTGGGTAGCGACTGTCTCTTTTACTTTGAGAAAATTAGAGTGTTCAAAGCAGGTATTCGCCTGAATATTACTCTTGGAATAATGCTATAAGACTTTGGTTCTAATGTATTGGTGATTGGGACCAGAGTAATGATTGATAGGGACGGTTGTGGTCTTACGTACTGCAAAGCGAGAGGTGAAATTCTTGGACCTTTGTATGACGAACACTGCGAAAGCATTGGACTAGGACGTTCCCG
>b388c90a25778f71d524419a062d9059e64e71a2_1
AGCTCCAATAGCGTATACTAATGTTGTTGCAGTTAAAAGCTCGTAGTCGGATTTCAGTAGGCTCAATTAAGTCTCTATTCTGAGTTCTTTATGGGCCTGCTTCTTTGACAGAAACTTCTATGTTATTCATTTAGCGTGGGTAGCGACTGTCTCTTTTACTTTGAGAAAATTAGAGTGTTCAAAGCAGGTATTCACCTGAATATTACTCTTGGAATAATGCTATAAGACTTTGGTTCTAATGTATTGGTGATTGGGACCAGAGTAATGATTGATAGGGACGGTTGGGGTCTTACGTACTGCAAAGCGAGAGGTGAAATTCTTGGACCTTTGTATGACGAACAACTGCGAAAGCATTGGACTAGGACGTTCCCG
>b6dfa2add37b684c8428fcce76254cf7291eed40_1
AGTTCCAATCACGTATACTAATGTTGTTGCAGTTAAAAACTCGTAGTTGGATTTCAGTAGGTTCAATTAAGTCTCTATTCTGAGTTCTTATGGTCCTGTTGTTTTGTACAGAAACTTCTATGTTATTCATTTAGTCGTGGGTAGCGACTGTCTCTTTACTTTGAGAAAATTAAAAGTGTTCAAAGCAGGTATTCGCCTGAATATTACTCTTGGAATAATGCTATAAGACTTTGGTTCTAATGTATTGGTGATTGGGACCAGAGTAATGATTGATAGGGACGGTTGGGGTCTTACGTACTGCAAAGCGAGAGGTGAAATTCTTGGACCTTTGTATGACGAACAACTGCGAAAGCATTGGACTAGGACGTTCCCG
>c9d692ccfadc1ca0689cee4f279482459addb0a8_1
AGCTCCAATAGCGTATACTAATGTTGTTGCAGTTAAAAGCTCGTAGTCGGATTTCAGTAGGCTCAATTAAGTCTCTATTCTGAGTTCTTTATGGGCCTGCTTCTTTGACAGAAACTTCTATGTTATTCATTTAGCGTGGGTAGCGACTGTCTCTTTTACTTTGAGAAAATTAGAGTGTTCAAAGCAGGTATTCGCCTGAATATTACTCTTGGAATAATGCTATAAGTACTTGGTTCTAATGTATTGGTGATTGGGACCAGAGTAATGATTGATAGGGACGGTTGGGGTCTTACGTACTGTAAAGCGAGAGGTGAAATTCTTGGACCTTTGTATGACGAACAACTGCGAAAGCATTGGACTAGGACGTTCCCG
>cbfd7775a2c0d6bfd542244ed610b19126d2a8f9_1
AGTTCCAATCACGTATACTAATGTTGTTGCAGTTAAAACTCGTAGTTGGATTTCAGTAGGTTCAATTAAGTCTCTATTCTGAGTTCTTTATGGTCCTGTTGTTTTGACAGAAACTTCTATGTTATTCATTTAGCGTGGGTAGCGACTGTCTCTTTTACTTTGAGAAAATTAAAGTGTTCAAAGCAGGTATTCGCCTGAATATTACTTTTGGAATAATGCTATAAGACTTTGGTTCTAATGTATTTGTGATTGGGACCAGAGTAATGATTGATAGGGACGGTTGAGGTCTTACGTACTGCAAAACGAGAGGTGAAATTCTTGGACCTTTGTATGACGAACAACTGCGAAAGCATTGGACTAGGACGTTCCCG
>eabc8988e40c776a9c3fdac9d54fb4f89b088795_1
AGCTCCAATAGCGTATACTAATGTTGTTGCAGTTAAAAGACTCGTAGTCGGATTTCAGTAGGCTCAATTAAGTCTCTATTCTGAGTTCTTTATGGGCCTGCTTCTTTGACAGAAACTTCTATGTTATTCATTTAGCGTGGGTAGCGACTGTCTCTTTTACTTTGAGAAAATTAGAGTGTTCAAAGCAGGTATTCGCCTGAATATTACTCTTGGAATAATGCTATAAGACTTTGGTTCTAATGTATTGGTGATTGGGACCAGAGTAATGATTGATAGGGACGGTTGGGGTCTTACGTACTGCAAAGCGAGAGGTGAAATTCTTGGACCTTTGTATGACGAACAACTGCGAAAGCATTGGACTAGGACGTTTCCCG
>fcea8ab94a59f73d571864018000a56841cbe4c2_1
AGCTCCAATAGCGTATACTAATGTTGTTGCAGTTAAAAGCTCGTAGTCGGATTTCAGTAGGCTCAATTAAGTCTCTATTCTGAGTTCTTTATGGGCCTGCTTCTTTGACAGAAACTTCTATGTTATTCATTTAGCGTGGGTAGCGACTGGTCTCTTTTACTTTGAGAAAATTAGAGTGTTCAAAGCAGGTATTCGCCTGAATATTACTCTTGGAATAATGCTATAAGACTTTGGTTCTAATGTATTGGTGATTGGGACCAGAGTAATGATTGATAGGGACGGTTGGGGTCTTACGTACTGCAAAGCGAGAGGTGAAATTCTTGGACCTTTGTATGACGACAACTGCGAAAGCATTGGACTAGGACGTTCCCG

# Ei44_1_V9_radiolaria.fas
>599a9d1ab5ed3e477599254458c741b14dac1691_598
GTCGCTCCTACCGATTGGATGATTCGGTAAGCTTTTGGGATTGATTGCGTATTTCTCATTGAGGGTACGTTAAAACAACTTAATCAAACCTAATCATCTAGAGGAAGGAGAAGTCGTAACAAGGTTTCC
>f85f078e9dc0ab7aa71e9d679471f24ea11a4045_26
GTCGCTCCTACCGATTGGATGATTCGGTAAGCTTTTGGGTATTGATTGCGTATTTCTCATTGAGGGTACGTTAAAACAACTTAATCAAACCTAATCATCTAGAGGAAGGAGAAGTCGTAACAAGGTTTCC
>ffdd6b43a06e827c764c0b8916ee3d58a3663928_14
GTCGCTCCTACCGATTGGATGATTCGGTAAGCTTTTGGGATTGATTAGCTACTTGTCACTGACATTAGCTTTTAACAACTTAATCAAACCTAATCATCTAGAGGAAGGAGAAGTCGTAACAAGGTTTCC
>5f852aee15fe11f22f250a2a8c850dbf43ca21c2_4
GTCGTTCCTACCGATTGGATGATTCGGTAACCTTTTGGGATTGATTGCGTATTTTCTCATTGAGGGTACGTTAAAACAACTTAATCAAACCTAATCATCTAGAGGAAGGAGAAGTCGTAACAAGGTTTCC
>08dbd22e229123e2d51f61d62373f828db032912_1
GTCGCTCCTACCGATTGGATGATTCGGTAAGCTTTTGGGATTGATTGCGTATTTCTCATTGAGGGTACGTTAAAACAACTTAATCAAACCTAATCATTTAGAGGAAGGAGAAGTCGTAACAAGGTTTCC
>2ae52fd7958714bb69c4c93664b31d0878720281_1
GTCGCTCCTACCGATTGGATGATTCGGTAAGCTTTTGGGATTGATTGCGTATTTCTCATTGAGGGTACGTTAAAACAACTTAATCAAACCTAATCATCTAGAGGAAGTAAAAGTCGTAACAAGGTTTCT
>40931cf9dd4e2202a429148bbda8d75ade0ec141_1
GTCGCTCCTACCGATTGGATGATTCGGTAAGCTTTTGGGATTGATTGCGTATTTCTCATTGAGGGTACGTTAAAACAACTTAATCAAACCTAATCATCTAGAGGAAGTAAAGTCGTAACAAGGTTTCT
>4274be1454cd30f319837f14c2cf7db4c284cabb_1
ATCGCTCCTACCGATTGGATGATTCGGTAAGCTTTTGGGATTGATTGCGTATTTCTCATTGAGGGTACGTTAAAACAACTTAATCAAACCTAATCATCTAGAGGAAGGAGAAGTCGTAACAAGGTTTCC
>482cfcf9c5a5d819299a605a9ea7a18ccbabf9f9_1
GTCACTCCTACCGATTGGATGATTCGGTAAGCTTTTGGGATTGATTGCGTATTTCTCATTGAGGGTACGTTAAAACAACTTAATCAAACCTAATCATCTAGAGGAAGGAGAAGTCGTAACAAGGTTTCC
>5f3fba90cc34ea0a96a370c029887dbb85e0104a_1
GTCGTTCCTACCGATTGGATGATTTGGTAAGTTTTTGGGATTGATTGAGTATTTTCTCATTGAGGGTACGTTAAAACAACTTAATCAAACCTAATCATCTAGAGGAAGGAGAAGTCGTAACAAGGTTTCC
>6c0dc0d4d58e8af433e99e3f59e5fbb725c72140_1
GTCGCTCCTACCGATTGGATGATTCGGTAAGCTTTTGGGATTGATTGCGTATTTCTCATTGGGGGTACGTTAAAACAACTTAATCAAACCTAATCATCTAGAGGAAGGAGAAGTCGTAACAAGGTTTCC
>6d09cee50127d4933c0823523fbf2d772e3cdef7_1
GTCGCCCCTACCGATTGGATGATTCGGTAAGCTTTTGGGATTGATTGCGTATTTCTCATTGAGGGTACGTTAAAACAACTTAATCAAACCTAATCATCTAGAGGAAGGAGAAGTCGTAACAAGGTTTCC
>81a6b10b1053037447cd59a182372e809f984fc6_1
GTCGCTCCTACCGATTGGATGATTCGGTAAATTTTGGGATTGATTGCGTATTTCTCATTGAGGGTACGTTAAAACAACTTAATCAAACCTAATCATCTAGAGGAAGAAGAAGTCGTAACAAGGTTTCC
>94b08a1132e6d918e16daa37124ed068de85f376_1
GTCGCTCCTACCGATTGGATGATTCGGTAAATTTTGGGATTGATTGCGTATTTTCTCATTGAGGGTACGTTAAAACAACTTAATCAAACCTAATCATCTAGAGGAAGAAGAAGTCGTAACAAGGTTTCC
>97b7b2890dfb2f8e2bb188743834738b3f5cfdd3_1
GTCGTTCCTACCGATTGGATGATTTGGTAAGTTTTGGGATTGATTGAGTATTTTCTCATTGAGGGTACGTTAAAACAACTTAATCAAACCTAATCATCTAGAGGAAGGAGAAGTCGTAACAAGGTTTCC
>ab26be4b24113350f00aaa7beeb7b6b159edcae2_1
GTCGTTCCTACCGATTGGATGATTTGGTAAGTTTTGGGATTGATTGAGTATTTCTCATTGAGGGTACGTTAAAACAACTTAATCAAACCTAATCATCTAGAGGAAGGAGAAGTCGTAACAAGGTTTCC
>c98790c760f454dff408889638adc49832124832_1
GTCGCTCCTACCGATTGGATGATTCGGTAAGCTTTTGGGATTGATTATGTATCTGTCATTGACGGTACATTTTAACAACTTAATCAAACCTAATCATCTAGAGGAAGGAGAAGTCGTAACAAGGTTTCC
>cb4de39220aea17e9fc26e426fa130f9bc0d119c_1
GTCGTTCCTACCGATTGGATGATTTGGTAAGTTTTTGGGGTTGATTGAGTATTTCTCATTGAGGGTACGTTAAAACAACTTAATCAAACCTAATCATCTAGAGGAAGGAGAAGTCGTAACAAGGTTTCC

# Ei44_2_V4_radiolaria.fas
>2aa596477e481a5c24ec1af501ea447585efe383_791
AGCTCCAATAGCGTATACTAATGTTGTTGCAGTTAAAAGCTCGTAGTCGGATTTCAGTAGGCTCAATTAAGTCTCTATTCTGAGTTCTTTATGGGCCTGCTTCTTTGACAGAAACTTCTATGTTATTCATTTAGCGTGGGTAGCGACTGTCTCTTTTACTTTGAGAAAATTAGAGTGTTCAAAGCAGGTATTCGCCTGAATATTACTCTTGGAATAATGCTATAAGACTTTGGTTCTAATGTATTGGTGATTGGGACCAGAGTAATGATTGATAGGGACGGTTGGGGTCTTACGTACTGCAAAGCGAGAGGTGAAATTCTTGGACCTTTGTATGACGAACAACTGCGAAAGCATTGGACTAGGACGTTCCCG
>ebe29081da7a93e3996da10f0331009b7421489e_43
AGCTCCAATAGCGTATACTAATGTTGTTGCAGTTAAAAGCTCGTAGTCGGATTTCAGTAGGCTCAATTAAGTCTCTATTCTGAGTTCTTTATGGGCCTGCTTCTTTGACAGAAACTTCTATGTTATTCATTTAGCGTGGGTAGCGACTGTCTCTTTTACTTTGAGAAAATTAGAGTGTTCAAAGCAGGTATTCGCCTGAATATTACTCTTGGAATAATGCTATAAGACTTTGGTTCTAATGTATTGGTGATTGGGACCAGAGTAATGATTGATAGGGACGGTTGGGTCTTACGTACTGCAAAGCGAGAGGTGAAATTCTTGGACCTTTGTATGACGAACAACTGCGAAAGCATTGGACTAGGACGTTCCCG
>d3be605345dc3e4f444a89c60334fdde85b82a7b_30
AGCTCCAATAGCGTATACTAATGTTGTTGCAGTTAAAAGACTCGTAGTCGGATTTCAGTAGGCTCAATTAAGTCTCTATTCTGAGTTCTTTATGGGCCTGCTTCTTTGACAGAAACTTCTATGTTATTCATTTAGCGTGGGTAGCGACTGTCTCTTTTACTTTGAGAAAATTAGAGTGTTCAAAGCAGGTATTCGCCTGAATATTACTCTTGGAATAATGCTATAAGACTTTGGTTCTAATGTATTGGTGATTGGGACCAGAGTAATGATTGATAGGGACGGTTGGGGTCTTACGTACTGCAAAGCGAGAGGTGAAATTCTTGGACCTTTGTATGACGAACAACTGCGAAAGCATTGGACTAGGACGTTCCCG
>fcdfafc671782f35d589f9b174458f6091155246_8
AGCTCCAATAGCGTATACTAATGTTGTTGCAGTTAAAAGCTCGTAGTCGGATTTCAGTAGGCTCAGTTAAGTCTCACTTATGAGTTCTTTATGGGCCTGCTTCTTTGACAGAAACTTCTATGTTATTCATTTAGCGTGGGTAGCGACTGTCTCTTTTACTTTGAGAAAATTAGAGTGTTCAAAGCAGGTATTCGCCTGAATATTACTCTTGGAATAATGCTATAAGACTTTGGTTCTAATGTATTGGTGATTGGGACCAGAGTAATGATTGATAGGGACGGTTGGGGTCTTACGTACTGCAAAGCGAGAGGTGAAATTCTTGGACCTTTGTATGACGAACAACTGCGAAAGCATTGGACTAGGACGTTCCCG
>287b1a121b8b7244f9f3ac0bc3da6578c3845bb7_6
AGCTCCAATAGCGTATACTAATGTTGTTGCAGTTAAAAGCTCGTAGTCGGATTTCAGTAGGCTCAATTAAGTCTCTATTCTGAGTTCTTTATGGGCCTGCTTCTTTGACAGAAACTTCTATGTTATTCATTTAGCGTGGGTAGCGACTGTCTCTTTTACTTTGAGAAAATTAGAGTGTTCAAAACAGGTATTCGCCTGAATATTACTCTTGGAATAATGCTATAAGACTTTGGTTCTAATGTATTGGTGATTGGGACCAGAGTAATGATTGATAGGGACGGTTGGGGTCTTACGTACTGCAAAGCGAGAGGTGAAATTCTTGGACCTTTGTATGACGAACAACTGCGAAAGCATTGGACTAGGACGTTCCCG
>3e3b2b603106a62e99b4ed915159ab7843c6d935_6
AGCTCCAATAGCGTATACTAATGTTGTTGCAGTTAAAAGCTCGTAGTCGGATTTCAGTAGGTTCAATTAAGTCTCTATTCTGAGTTCTTTATGGGCCTGCTTCTTTGACAGAAACTTCTATGTTATTCATTTAGCGTGGGTAGCGACTGTCTCTTTTACTTTGAGAAAATTAGAGTGTTCAAAGCAGGTATTCGCCTGAATATTACTCTTGGAATAATGCTATAAGACTTTGGTTCTAATGTATTGGTGATTGGGACCAGAGTAATGATTGATAGGGACGGTTGGGGTCTTACGTACTGCAAAGCGAGAGGTGAAATTCTTGGACCTTTGTATGACGAACAACTGCGAAAGCATTGGACTAGGACGTTCCCG
>541ce020752d514359919e6856d49c08eb557d32_6
AGCTCCAATAGCGTATATTAAAGTTGCTGCAGTTAAAAGCTCGTAGTCGGATTTCAGTAGGCTCAATTAAGTCTCTATTCTGAGTTCTTTATGGGCCTGCTTCTTTGACAGAAACTTCTATGTTATTCATTTAGCGTGGGTAGCGACTGTCTCTTTTACTTTGAGAAAATTAGAGTGTTCAAAGCAGGTATTCGCCTGAATATTACTCTTGGAATAATGCTATAAGACTTTGGTTCTAATGTATTGGTGATTGGGACCAGAGTAATGATTGATAGGGACGGTTGGGGTCTTACGTACTGCAAAGCGAGAGGTGAAATTCTTGGACCTTTGTATGACGAACAACTGCGAAAGCATTGGACTAGGACGTTCCCG
>b84dbecdcd2a5b685f491c54ba2eb558918a4026_6
AGCTCCAATAGCGTATACTAATGTTGTTGCAGTTAAAAGACTCGTAGTCGGATTTCAGTAGGCTCAATTAAGTCTCTATTCTGAGTTCTTTATGGGCCTGCTTCTTTGACAGAAACTTCTATGTTATTCATTTAGCGTGGGTAGCGACTGTCTCTTTTACTTTGAGAAAATTAGAGTGTTCAAAGCAGGTATTCGCCTGAATATTACTCTTGGAATAATGCTATAAGACTTTGGTTCTAATGTATTGGTGATTGGGACCAGAGTAATGATTGATAGGGACGGTTGGGTCTTACGTACTGCAAAGCGAGAGGTGAAATTCTTGGACCTTTGTATGACGAACAACTGCGAAAGCATTGGACTAGGACGTTCCCG
>3226033308c6b6424b41fc534b7ff8e490b2077b_5
AGCTCCAATAGCGTATATTAAAGTTGTTGCAGTTAAAAGCTCGTAGTCGGATTTCAGTAGGCTCAATTAAGTCTCTATTCTGAGTTCTTTATGGGCCTGCTTCTTTGACAGAAACTTCTATGTTATTCATTTAGCGTGGGTAGCGACTGTCTCTTTTACTTTGAGAAAATTAGAGTGTTCAAAGCAGGTATTCGCCTGAATATTACTCTTGGAATAATGCTATAAGACTTTGGTTCTAATGTATTGGTGATTGGGACCAGAGTAATGATTGATAGGGACGGTTGGGGTCTTACGTACTGCAAAGCGAGAGGTGAAATTCTTGGACCTTTGTATGACGAACAACTGCGAAAGCATTGGACTAGGACGTTCCCG
>77dce71a717f41fa86b44198601284b9c75b2f46_4
AGTTCCAATAGCGTATACTAATGTTGTTGCAGTTAAAAGCTCGTAGTCGTATTTCAGTAGGTTCAATTAAGTCTCTATTCTGAGTTCTTTATGGTCCTGTTGTTTTGACAGAAACTTCTATGTTATTCATTTAGCGTGGGTAGCGACTGTCTCTTTTACTTTGAGAAAATTAGAGTGTTCAAAGCAGGTATTCGCCTGAATATTACTCTTGGAATAATGCTATAAGACTTTGGTTCTAATGTATTGGTGATTGGGACCAGAGTAATGATTGATAGGGACGGTTGGGGTCTTACGTACTGCAAAACGAGAGGTGAAATTCTTGGACCTTTGTATGACGAACAACTGCGAAAGCATTGGACTAGGACGTTCCCG
>c42d75edbc1227f29bbc62fbdc71d04c762f0be7_4
AGCTCCAATAGCGTATACTAATGTTGTTGCAGTTAAAAGCTCGTAGTCGGATTTCAGTAGGCTCAATTAAGTCTCTATTCTGAGTTCTTTATGGGCCTGCTTCTTTGACAGAAACTTCTATGTTATTCATTTAGCGTGGGTAGCGACTGTCTCTTTTACTTTGAGAAAATTAGAGTGTTCAAAGCAGGTATTCGCCTGAATATTACTCTTGGAATAATGCTATAAGACTTTGGTTCTAATGTATTGGTGATTGGGACCAGAGTAATGATTGATAGGGACGGTTGGGTCGTTACGTACTGCAAAGCGAGAGGTGAAATTCTTGGACCTTTGTATGACGAACAACTGCGAAAGCATTGGACTAGGACGTTCCCG
>00d4bd543be05e2338d3f550fb63c8ab6d53270f_3
AGCTCCAATAGCGTATACTAATGTTGTTGCAGTTAAAAGCTCGTAGTCGGATTTCAGTAAGCTCAGTTAAGTCTCTATTCTGAGTTCTTTATGGGCCTGTTTCTTTGACAGAAACTTCTATGTTATTCATTTAGCGTGGGTAGCGACTGTCTCTTTTACTTTGAGAAAATTAGAGTGTTCAAAGCAGGTATTCGCCTGAATATTACTCTTGGAATAATGCTATAAGACTTTGGTTCTAATGTATTGGTGATTGGGACCAGAGTAATGATTGATAGGGACGGTTGGGGTCTTACGTACTGCAAAGCGAGAGGTGAAATTCTTGGACCTTTGTATGACGAACAACTGCGAAAGCATTGGACTAGGACGTTCCCG
>7f20ce0fe9a7dd499e08a51adf0f1f218b2ff528_3
AGCTCCAATAGCGTATACTAATGTTGTTGCAGTTAAAAGACTCGTAGTCGGATTTCAGTAGGCTCAATTAAAGTCTCTATTCTGAGTTCTTTATGGGCCTGCTTCTTTGACAGAAACTTCTATGTTATTCATTTAGCGTGGGTAGCGACTGTCTCTTTTACTTTGAGAAAATTAGAGTGTTCAAAGCAGGTATTCGCCTGAATATTACTCTTGGAATAATGCTATAAGACTTTGGTTCTAATGTATTGGTGATTGGGACCAGAGTAATGATTGATAGGGACGGTTGGGGTCTTACGTACTGCAAAGCGAGAGGTGAAATTCTTGGACCTTTGTATGACGAACAACTGCGAAAGCATTGGACTAGGACGTTCCCG
>a3fe9fba39cd8baf8148a65b59bb88ef79d0d45a_3
AGTTCCAATCACGTATACTAATGTTGTTGCAGTTAAAACTCGTAGTTGGATTTCAGTAGGTTCAATTAAGTCTCTATTCTGAGTTCTTTATGGTCCTGTTGTTTTTGACAGAAACTTCTATGTTATTCATTTAGCGTGGGTAGCGACTGTCTCTTTTACTTTGAGAAAATTAAAGTGTTCAAAGCAGGTATTCGCCTGAATATTACTTTTGGAATAATGCTATAAGACTTTGGTTCTAATGTATTTGTGATTGGGACCAGAGTAATGATTGATAGGGACGGTTGAGGTCTTACGTACTGCAAAACGAGAGGTGAAATTCTTGGACCTTTGTATGACGAACAACTGCGAAAGCATTGGACTAGGACGTTCCCG
>90f5941b5097b4e3ac8da6dd7d14a6a979530744_2
AGCTCCAATAGCGTATACTAATGTTGTTGCAGTTAAAAGCTCGTAGTCGGATTTCAGTAGGCTCAATTAAGTCTCTATTCTGAGTTCTTTATGGGCCTGTTTCTTTGACAGAAACTTCTATGTTATTCATTTAACGTGGGTAGCGACTGTCTCTTTTACTTTGAGAAAATTAGAGTGTTCAAAGCAGGTATTCGCCTGAATATTACTCTTGGAATAATGCTATAAGACTTTGGCTCTAATGTATTGGTGATTGGGACCAGAGTAATGATTGATAGGGACGGTTGGGGTCTTACGTACTGCACAGCGAGAGGTGAAATTCTTGGACCTTTGTATGACGAACAACTGCGAAAACATTGGACTAGGACGTTCCCG
>038f28a9979a26e80c0192c3c5549f94c0d96011_1
AGCTCCAATAGCGTATACTAATGTTGTTGCAGTTAAAAGACTCGTAGTCGGATTTCAGTAGGCTCAATTAAGTCTCTATTCTGAGTTCTTTATGGGCCTGCTTCTTTGACAGAAACTTCTATGTTATTCATTTAGCGTGGGTAGCGACTGTCTCTTTTACTTTGAGAAAATTAGAGTGTTCAAAGCAGGTATTCGCCTGAATATTACTCTTGGAATAATGCTATAAGACTTTGGTTCTAATGTATTGGTGATTGGGGACCAGAGTAATGATTGATAGGGACGGTTGGGGTCTTACGTACTGCAAAGCGAGAGGTGAAATTCTTGGACCTTTGTATGACGAACAACTGCGAAAGCATTGGACTAGGACGTTCCCG
>0c1453dca1458f5393010ff1245207b1b83739a0_1
AGTTCCAATCACGTATACTAATGTTGTTGCAGTTAAAAACTACGTAGTTGGATTTCAGTAGGTTCAATTAAGTCTCTATTCTGAGTTCTTTATGGTCCTGTTGTTTTGACAGAAACTTCTATGTTATTCATTTAGCGTGGGTAGCGACTGTCTCTTTTACTTTGAGAAAATTAAAGTGTTCAAAAGCAGGTATTCGCCTGAATATTACTTTTGGAATAATGCTATAAGACTTTGGTTCTAATGTATTTGTGATTGGGACCAGAGTAATGATTGATAGGGACGGTTGAGGTCTTACGTACTGCAAAACGAGAGGTGAAATTCTTGGACCTTTGTATGACGAACAACTGCGAAAGCATTGGACTGGACGTTCCTG
>115a335426000e145dcfc2afc75bc26e0aef58a1_1
AGCTCCAATAGCGTATACTAATGTTGTTGCAGTTAAAAGCTCGTAGTCGGATTTCAGTAGGCTCAATTAAGTCTCTATTCTGAGTTCTTTATGGGCCTGCTTCTTTGACAGAAACTTCTATGTTATTCATTTAGCGTGGGTAGCGACTGTCTCTTTTACTTTGAGAAAATTAGAGTGTTCAAAACAGGTATTCGCCTGAATATTACTCTTGGAATAATGCTATAAGACTTTGGTTCTAATGTATTGGTGATTGGGACCAGAGTAATGATTGATAGGGACGGTTGGGGTCTTACGTACTGCAAAGCGAGAGGTGAAATTCTTGGACCTTTGTATGACGAACAACTGCGAAGCATTGGACTAGGACGTTCCCG
>14676ca5b4df8b9290d4b94d869f27235f227b3f_1
AGCTCCAATAGCGTATACTAATGTTGTTGCAGTTAAAAGACTCGTAGTCGGATTTCAGTAGGCTCAATTAAGTCTCTATTCTGAGTTCTTTATGGGCCTGCTTCTTTGACAGAAACTTCTATGTTATTCATTTAGCGTGGGTAGCGACTGTCTCTTTTACTTTGAGAAAATTAGAGTGTTCAAAGCAGGTATTCGCCTGAATATTACTCTTGGAATAATGCTATAAGACTTTGGTTCTAATGTATTGGTGATTGGGACCAGAGTAATGATTGATAGGGGACGGTTTGGGTCTTACGGTACTGCAAAGCGAGAGGTGAAATTCTTGGACCTTTGTATGACGAACAACTGCGAAAGCATTGGACTAGGACGTTCCCG
>25536ca13d9c2a6579442751cbff68305141f4ef_1
AGCTCCAATAGCGTATACTAATGTTGTTGCAGTTAAAAGCTCGTAGTCGGATTTCAGTAGGCTCAATTAAGTCTCTATTCTGAGTTCTTTATGGGCCTGCTTCTTTGACAGAAACTTCTATGTTATTCATTTAGCGTGGGTAGCGACTGTCTCTTTTACTTTGAGAAAATTAGAGTGTTCAAAGCAGGTATTCGCCTGAATATTACTCTTGGAATAATGCTATAAGACTTTGGTTCTAATGTATTGGTGATTGGGACCAGAGTAATGATTGATAGGGACGGTTGGGGTCTTACGTACTGCAAAGCGAGAGGCGAAATTCTTGGACCTTTGTATGACGAACAACTGCGAAAGCATTGGACTAGGACGTTCCCG
>261c8e97426bdbd920cac5de61647c58401b8fdd_1
AGCTCCAATAGCGTATACTAATGTTGTTGCAGTTAAAAGCTCGTAGTCGGATTTCAGTAGGCTCAATTAAGTCTCTATTCTGAGTTCTTTATGGGCCTGCTTCTTTGACAGAAACTTCTATGTTATTCATTTAGCGTGGGTAGCGACTGTCTCTTTTACTTTGAGAAAATTAGAGTGTTCAAAGCAGGTATTCGCCTGAATATTACTCTTGGAATAATGCTATAAGACTTTGGTTCTAATGTATTGGTGATTGGGACCAGAGTAATGATTGATAGGGACGGTTGGGGTCTTACGTACTGCAAAGCGAGAGGTGAAATTCTTGGACCTTTGTATGACGAACAACTGTGAAAGCATTGGACTAGGACGTTCCCG
>261ed51b262ba22b9045d102112c91a784b953e3_1
AGTTCCAATCACGTATACTAATGTTGTTGCAGTTAAAACTACGTAGTTGGATTTCAGTAGGTTCAATTAAGTCTCTATTCTGAGTTCTTTATGGTCCTGTTGTTTTGACAGAAACTTCTATGTTATTCATTTAGCGTGGGTAGCGACTGTCTCTTTTACTTTGAGAAAATTAAAGTGTTCAAAGCAGGTATTCGCCTGAATATTACTTTGGAATAATGCTATAAGACTTTGGTTCTAATGTATTTGTGATTGGGACCAGAGTAATGATTGATAGGGACGGTTGAGGTCTTACGTACTGCAAAACGAGAGGTGAAATTCTTGGACCTTTGTATGACGAACAACTGCGAAAGCATTGGACTAGGACGTTCCCG
>32bb3c9aef5bcd88a2508c193f034bdde80e404e_1
AGTTCCAATCACGTATACTAATGTTGTTGCAGTTAAAAACTCGTAGTTGGATTTCAGTAGGTTCAATTAAGTCTCTATTCTGAGTTCTTTATGGTCCTGTTGTTTTTGACAGAAACTTCTATGTTATTCATTTAGCGTGGGTAGCGACTGTCTCTTTTACTTTGAGAAAATTAAAGTGTTCAAAGCAGGTATTCGCCTGAATATTACTTTTGGAATAATGCTATAAGACTTTGGTTCTAATGTATTTGTGATTGGGGACCAGAGTAATGATTGATAGGGACGGTTGGGGTCTTACGTACTGCAAAGCGAGAGGTGAAATTCTTGGACCTTTGTATGACGAACAACTGCGAAAGCATTGGACTAGGACGTTCCCG
>37954377be4684b3595fa7cb4c8f0b9b17f6fb0b_1
AGCTCCAATAGCGTATACTAATGTTGTTGCAGTTAAAAGACTCGTAGTCGGATTTCAGTAGGCTCAATTAAGTCTCTATTCTGAGTTCTTTATGGGCCTGCTTCTTTGACAGAAACTTCTATGTTATTCATTTAGCGTGGGTAGCGACTGTCTCTTTTACTTTGAGAAAATTAGAGTGTTCAAAGCAGGTATTCGCCTGAATATTACTCTTGGAATAATGCTATAAGACTTTGGTTCTAATGTATTGGTGATTGGGACCAGAGCAATGATTGATAGGGACGGTTGGGGTCTTACGTACTGCAAAGCGAGAGGTGAAATTCTTGGACCTTTGTATGACGAACACTGCGAAAGCATTGGACTAGGACGTTCCCG
>43e889bf66cc6abde3efacc20d9e43f6c97678eb_1
AGCTCCAATAGCATATACTAATGTTGTTGCAGTTAAAAGCTCGTAGTCGGATTTCAGTAGGCTCAATTAAGTCTCTATTCTGAGTTCTTTATGGGCCTGCTTCTTTGACAGAAACTTCTATGTTATTCATTTAGCGTGGGTAGCGACTGTCTCTTTTACTTTGAGAAAATTAGAGTGTTCAAAGCAGGTATTCGCCTGAATATTACTCTTGGAATAATGCTATAAGACTTTGGTTCTAATGTATTGGTGATTGGGACCAGAGTAATGATTGATAGGGACGGTTGGGGTCTTACGTACTGCAAAGCGAGAGGTGAAATTCTTGGACCTTTGTATGACGAACAACTGCGAAAGCATTGGACTAGGACGTTCCCG
>46128549254a3b5cc66363ff8ddd4564a8de8ac9_1
AGCTCCAATAGCGTATACTAATGTTGTTGCAGTTAAAAGCTCGTAGTCGGATTTCAGTAGGCTCAATTAAGTCTCTATTCTGAGTTCTTTATGGGCCTGCTTCTTTGACAGAAACTTCTATGTTATTCATTTAGCGTGGGTAGCGACTGTCTCTTTTACTTTGAGAAAATTAGAGTGTTCAAAGCAGGTATTCGCCTGAATATTACTCTTGGAATAATGCTATAAGACTTTGGTTCTAATGTATTGGTGATTGGGACCAGAGTAATGATTGATAGGGACGGTTGGGGTCTTACGTACTGCAAAGCGAGAGGTGAAATTCTTGGACCTTTGTATGACGAACAACTGCGAAAGCATTTGCCAAGAATGTTTTCA
>59ad329249883e2d027daacb1c3df7f453bc1af9_1
AGCTCCAATAGCGTACACTAATGTTGTTGCAGTTAAAAGCTCGTAGTCGGATTTCAGTAGGCTCAATTAAGTCTCTATTCTGAGTTCTTTATGGGCCTGCTTCTTTGACAGAAACTTCTATGTTATTCATTTAGCGTGGGTAGCGACTGTCTCTTTTACTTTGAGAAAATTAGAGTGTTCAAAGCAGGTATTCGCCTGAATATTACTCTTGGAATAATGCTATAAGACTTTGGTTCTAATGTATTGGTGATTGGGACCAGAGTAATGATTGATAGGGACGGTTGGGGTCTTACGTACTGCAAAGCGAGAGGTGAAATTCTTGGACCTTTGTATGACGAACAACTGCGAAAGCATTGGACTAGGACGTTCCCG
>5f728379c12841596ca7536f7064d561a40f61ba_1
AGCTCCAATAGCGTATACTAATGTTGTTGCAGTTAAAAGCTCGTAGTCGGATTTCAGTAGGCTCAGTTTAGTCTTCATTGTAAGATCTATATGGGCCTGCTTCTTTGACAGAAACTTCTATGTTATTCATTTAGCGTGGGCAGCGACTGTCTCTTTTACTTTGAGAAAATTAGAGTGTTCAAAGCAGGTAGATGCCTGAATATTACTCTTGGAATAATGCTATAAGACTTTGGTTCTAATGTATTGGTGATTGGGACCAGAGTAATGATTGATAGGGACGGTTGGGGTCTTACGTACTGCAAAGCGAGAGGTGAAATTCTTGGACCTTTGTATGCGAACAACTGCGAAAGCATTGGACTAGGACGTTCCCG
>646e9f154228a2a4fe516e1709f456ea870e7670_1
AGCTCCAATAGCGTATACTAATGTTGTTGCAGTTAAAAGCTCGTAGTCGGATTTCAGTAGGCTCAATTAAGTCTCTATTCTGAGTTCTTTATGGGCCTGCTTCTTTGACAGAAACTTCTATGTTATTCATTTAGCGTGGGTAGCGACTGTCTCTTTTACTTTGAGAAAATTAGAGTGTTCAAAGCAGGTATTCGCCTGAATATTACTCTTGGAATAATGCTATAAGACTTTGGTTCTAATGTATTGGTGATTGGGACCAGAGTAATGATTGATAGGGGACGGTTGGGGTCGTTACGTACTGCAAAGCGAGAGGTGAAATTCTTGGACCTTTGTATGACGAACAACTGCGAAAGCATTGGACTAGGACGTTCCCG
>6e4b7ef62250dbab8188863d0fb9010d3989513e_1
AGCTCCAATAGCGTATACTAATGTTGTTGCAGTTAAAAGCTCGTAGTCGGATTTCAGTAGGTTCAATTAAGTCTCTATTCTGAGTTCTTTATGGGCCTGCTTCTTTGACAGAAACTTCTATGTTATTCATTTAGCGTGGGTAGCGACTGTCTCTTTTACTTTGAGAAAATTAGAGTGTTCAAAGCAGGTATTCGCCTGAATATTACTCTTGGAATAATGCTATAAGACTTTGGTTCTAATGTATTGGTGATTGGGACCAGAGTAATGATTGATAGGGACGGTTGGGGTCTTACGTACTGCAAAAGCGAGAGGTGAAAATTCTTGGACCTTTGTATGACGAACAAACTGCGAAAGCATTGGACTAGGACGTTCCCG
>6ee6b901216fe9d3e3b199f5c41571fa337d26d8_1
AGCTCCAATAGCGTATATTAAAGTTAGTTGCAGTTAAAAAGACTCGTAGTCGGATTTCAGTAGGCTCAATTAAAGTCTCTATTCTGAGTTCTTTATGGGCCTGCTTCTTTGACAGAAACTTCTATGTTATTCATTTAGCGTGGGTAGCGACTGTCTCTTTTACTTTGAGAAAATTAGAGTGTTCAAAGCAGGTATTCGCCTGAATATTACTCTTGGAATAATGCTATAAGACTTTGGTTCTAATGTATTGGTGATTGGGACCAGAGTAATGATTGATAGGGACGGTTGGGGTCTTACGTACTGCAAAGCGAGAGGTGAAATTCTTGGACCTTTGTATGACGAACAACTGCGAAGCATTGGACTAGGACGTTCCCG
>715ee9cfeaebe8d62b6516ef6fd90113d05e206c_1
AGTTCCAATCACGTATACTAATGTTGTTGCAGTTAAAAACTACGTAGTTGGATTCAGTAGGTTCAATTAAGTCTCTATTCTGAGTTCTTTATGGTCCTGTTGTTTTGACAGAAACTTCTATGTTATTCATTTAGCGTGGGTAGCGACTGTCTCTTTTACTTTGAGAAAAATTAGAGTGTTCAAAAGCAGGTATTCGCCTGAATATTACTCTTGGAATAATGCTATAAGACTTTGGTTCTAATGTATTGGTGATTGGGGACCAGAGTAATGATTGATAGGGACGGTTGGGGTCTTACGTACTGCAAAGCGAGAGGTGAAATTCTTGGACCTTTGTATGACGAACAACTGCGAAAGCATTGGACTAGGACGTTCCCG
>72807760b04698d4d083d09315cc1d130dfea42b_1
AGCTCCAATAGCGTATACTAATGTTGTTGCAGTTAAAAGACTCGTAGTCGGATTTCAGTAGGCTCAATTAAGTCTCTATTCTGAGTTCTTTATGGGCCTGCTTCTTTGACAGAAACTTCTATGTTATTCATTTAGCGTGGGTAGCGACTGTCTCTTTTACTTTGAGAAAATTAGAGTGTTCAAAGCAGGTATTCGCCTGAATATTACTCTTGGAATAATGCTATAAGACTTTGGTTCTAATGTATTGGTGATTGGGACCAGAGTAATGATTGATAGGGACGGTTGGGGTCTTACGTACTGCAAAGCGAGAGGTGAAATTCTTGGACCTTTGTATGACGAACAATTGCGAAAGCATTGGACTAGGACGTTCCCG
>7a2c5d83e3b0c6afabe8d0b551372ae5414969d2_1
AGCTCCAATAGCGTATACTAATGTTGTTGCAGTTAAAAGCTCGTAGTCGGATTTCAGTAGGCTCAATTAAGTCTCTATTCTGAGTTCTTTATGGGCCTGCTTCTTTGACAGAAACTTCTATGTTATTCATTTAGCGTGGGTAGCGACTGTCTCTTTTACTTTGAGAAAATTAGAGTGTTCAAAGCAGGTATTCGCCTGAATATTACTCTTGGAATAATGCTATAAGACTTTGGTTCTAATGTATTGGTGATTGGGACCAGAGTAATGATTGATAGGGACGGCCGGGGTCTTACGTACTGCAAAGCGAGAGGTGAAATTCTTGGACCTTTGTATGACGAACAACTGCGAAAGCATTGGACTAGGACGTTCCCG
>8153b384dd7bbc43b8815754bbd189157fd35b24_1
AGCTCCAATAGCGTATACTAATGTTGTTGCAGTTAAAAGACTCGTAGTCGGATTTCAGTAGGCTCAATTAAGTCTCTATTCTGAGTTCTTTATGGGCCTGCTTCTTTGACAGAAACTTCTATGTTATTCATTTAGCGTGGGTAGCGACTGTCTCTTTTACTTTGAGAAAATTAGAGTGTTCAAAGCAGGTATTCGCCTGAATATTACTCTTGGAATAATGCTATAAGACTTTGGTTCTAATGTATTGGTGATTGGGACCAGAGTAATGATTGATAGGGGACGGTTGGGTCTTACGTACTGCAAAGCGAGAGGTGAAATTCTTGGACCTTTGTATGACGAACAACTGCGAAAGCATTGGACTAGGACGTTCCCG
>8e351aed707e138a66654e808a8af1e121fe7010_1
AGCTCCAATAGCGTATACTAATGTTGTTGCAGTTAAAAGCTCGTAGTCGGATTTCAGTAGGCTCAATTAAGTCTCTATTCTGAGTTCTTTATGGGCCTGCTTCTTTGACAGAAACTTCTATGTTATTCATTTAGCGTGGGTAGCGACTGTCTCTTTTACTTTGAGAAAATTAGAGTGTTCAAAGCAGGTATTCGCCTGAATATTACTCTTGGAATAATGCTATAAGACTTTGGTTCTAATGTATTGGTGATTGGGACCAGAGTAATGATTGATAGGGACGGTTGGGTCGTTACGTACTGCAAAGCGAGAGGTGAAATTCTTGGACCTTTGTATGACGAACAACTGCGAAAGCATTGGACTAGGACGTTCCG
>8e5ea623791c16fd5c502c915be583296264269d_1
AGCTCCAATAGCGTATACTAATGTTGTTGCAGTTAAAAGCTCGTAGTCGGATTTCAGTAGGCTCAATTAAGTCTCTATTCTGAGTTCTTTATGGGCCTGCTTCTTTGACAGAAACTTCTATGTTATTCATTTAGCGTGGGTAGCGACTGTCTCTTTTACTTTGAGAAAATTAGAGTGTTCAAAGCAGGTATTCGCCTGAATATTACTCTTGGAATAATGCTATAAGACTTTGGTTCTAATGTATTGGTGATTGGGACCAGAGTAATGATTGATAGGGACGGTTGGGCTTACGTACTGCAAAGCGAGAGGTGAAATCTTGGACCTTTGTATGACGAACAACTGCGAAAGCATTGGACTAGGACGTTCCCG
>9705d968d9f30d8cc53fbeda9b8cbd0d92a6c6a4_1
AGCTCCAATAGCGTATACTAATGTTGTTGCAGTTAAAAGCTCGTAGTCGGATTTCAGTAGGCTCAATTAAGTCTCTATTCTGAGTTCTTTATGGGCCTGCTTCTTTGACAGAAACTTCTATGTTATTCATTTAGCGTGGGCAGCGACTGTCTCTTTTACTTTGAGAAAATTAGAGTGTTCAAAGCAGGTAGATGCCTGAATATTACTCTTGGAATAATGCTATAAGACTTTGGTTCTAATGTATTGGTGATTGGGACCAGAGTAATGATTGATAGGGACGGTTGGGGTCTTACGTACTGCAAAGCGAGAGGTGAAATTCTTGGACCTTTGTATGACGACAACTGCGAAAGCATTGGACTAGGACGTTCCCG
>991a3889cf01194f2e54f725ae1c5ccf3a33e762_1
AGTTCCAATCACGTATACTAATGTTGTTGCAGTTAAAAACTCGTAGTTGGATTTCAGTAGGTTCAATTAAGTCTCTATTCTGAGTTCTTTATGGGCCTGCTTCTTTGACAGAAACTTCTATGTTATTCATTTAGCGTGGGTAGCGACTGTCTCTTTTACTTTGAGAAAATTAGAGTGTTCAAAGCAGGTATTCGCCTGAATATTACTCTTGGAATAATGCTATAAGACTTTGGTTCTAATGTATTGGTGATTGGGACCAGAGTAATGATTGATAGGGACGGTTGGGGTCTTACGTACTGCAAAGCGAGAGGTGAAATTCTTGGACCTTTGTATGACGAACAACTGCGAAAGCATTGGACTAGGACGTTCCCG
>ab12e926b4b451226e4b8345d6913e286d22dc6e_1
AGCTCCAATAGCGTATACTAATGTTGTTGCAGTTAAAAGCTCGTAGTCGGATTTCAGTAGGCTCAATTAAGTCTCTATTCTGAGTTCTTTATGGGCCTGCTTCTTTGACAGAAACTTCTATGTTATTCATTTAGCGTGGGTAGCGACTGTCTCTTTTACTTTGAGAAAATTAGAGTGTTCAAAGCAGGTATTCGCCTGAATATTACTCTTGGAATAATGCTATAAGACTTTGGTTCTAATGTATTGGTGATTGGGACCAGAGTAATGATTGATAGGGGACGGTTTGGGTCGTTACGTACTGCAAAGCGAGAGGTGAAATTCTTGGACCTTTGTATGACGAACAACTGCGAAAGCATTGGACTAGGACGTTCCCG
>ae7c0e0cd134bf01d0d25aecd2f3bda267db7022_1
AGCTCCAATAGCGTATACTAATGTTGTTGCAGTTAAAAGCTCGTAGTCGGATTTCAGTAGGCTCAATTAAGTCTCTATTCTGAGTTCTTTATGGGCCTGCTTCTTTGACAGAAACTTCTATGTTATTCATTTAGCATGGGTAGCGACTGTCTCTTTTACTTTGAGAAAATTAGAGTGTTCAAAGCAGGTATTCGCCTGAATATTACTCTTGGAATAATGCTATAAGACTTTGGTTCTAATGTATTGGTGATTGGGACCAGAGTAATGATTGATAGGGACGGTTGGGGTCTTACGTACTGCAAAGCGAGAGGTGAAATTCTTGGACCTTTGTATGACGAACACTGCGAAAGCATTGGACTAGGACGTTCCCG
>b3fc97aa6709a2a1b9a125a024e4d32637b2774d_1
AGCTCCAATAGCGTATACTAATGTTGTTGCAGTTAAAAGCTCGTAGTCGGATTTCAGTAGGCTCAATTAAGTCTCTATTCTGAGTTCTTTATGGGCCTGCTTCTTTGACAGAAACTTCTATGTTATTCATTTAGCGTGGGTAGCGACTGTCTCTTTTACTTTGAGAAAATTAGAGTGTTCAAAGCAGGTATTCGCCTGAATATTACCCTTGGAATAATGCTATAAGACTTTGGTTCTAATGTATTGGTGATTGGGACCAGAGTAATGATTGATAGGGACGGTTGGGGTCTTACGTACTGCAAAGCGAGAGGTGAAATTCTTGGACCTTTGTATGACGAACAACTGCGAAAGCATTGGACTAGGACGTTCCCG
>c2dcab7a8373baf3341ecf99dbdfb3d1c13be0f4_1
AGCTCCAATAGCGTATACTAATGTTGTTGCAGTTAAAAGACTCGTAGTCGGATTTCAGTAGGCTCAGTTTAGTCTTCATTGTAAGATCTATATGGGCCTGCTTCTTTGACAGAAACTTCTATGTTATTCATTTAGCGTGGGCAGCGACTGTCTCTTTTACTTTGAGAAAATTAGAGTGTTCAAAGCAGGTATTCGCCTGAATATTACTCTTGGAATAATGCTATAAGACTTTGGTTCTAATGTATTGGTGATTGGGACCAGAGTAATGATTGATAGGGACGGTTGGGGTCTTACGTACTGCAAAGCGAGAGGTGAAATTCTTGGACCTTTGTATGACGACAACTGCGAAAGCATTGGACTAGGACGTTCCCCG
>c36b090a52124b2aa310df245517a564441f354d_1
ATCTCCAATAGCGTATACTAATGTTGTTGCAGTTAAAAGCTCGTAGTCGGATTTCAGTAGGCTCAATTAAGTCTCTATTCTGAGTTCTTTATGGGCCTGCTTCTTTGACAGAAACTTCTATGTTATTCATTTAGCGTGGGTAGCGACTGTCTCTTTTACTTTGAGAAAATTAGAGTGTTCAAAGCAGGTATTCGCCTGAATATTACTCTTGGAATAATGCTATAAGACTTTGGTTCTAATGTATTGGTGATTGGGACCAGAGTAATGATTGATAGGGACGGTTGGGGTCTTACGTACTGCAAAGCGAGAGGTGAAATTCTTGGACCTTTGTATGACGAACAACTGCGAAAGCATTGGACTAGGACGTTCCCG
>c564aba14038e41493da2ec483fb9cce2aa15bf9_1
AGCTCCAATAGCGTATACTAATGTTGTTGCAGTTAAAAGACTCGTAGTCGGATTTCAGTAGGCTCAATTAAGTCTCTATTCTGAGTTCTTTATGGGCCTGCTTCTTTGACAGAAACTTCTATGTTATTCATTTAGCGTGGGTAGCGACTGTCTCTTTTACTTTGAGAAAATTAGAGTGTTCAAAGCAGGTATTCGCCTGAATATTACTCTTGGAATAATGCTATAAGACTTTGGTTCTAATGTATTGGTGATTGGGACCAGAGTAATGATTGATAGGGGACGGTTGGGGTCTTACGGTACTGCAAAGCGAGAGGTGAAATTCTTGGACCTTTGTATGACGAACAACTGCGAAAGCATTGGACTAGGACGTTCCCG
>c7386fb2a89e611b29ecb0eb409f7237176b51a1_1
AGCTCCAATAGCGTATACTAATGTTGTTGCAGTTAAAAGCTCGTAGTCGGATTTCAGTAGGCTCAATTAAGTCTCTATTCTGAGTTCTTTATGGGCCTGCTTCTTTGACAGAAACTTCTATGTTATTCATTTAGCGTGGGTAGCGACTGTCTCTTTTACTTTGAGAAAATTAGAGTGTTCAAAGCAGGTATTCGCCTGAATATTACTCTTGGAATAATGCTATAAGACTTTGGTTCTAATGTATTGGTGATTGGGACCAGAGTAATGATTGATAGGGACGGTTGGGTCGTTACGTACTGCAAAGCGAGAGGTGAAATTACTTGGACCTTTGTATGACGAACAACTGCGAAAGCATTGGACTAGGACGTTCCCG
>d1759d87ff72bd59c78876fc61edcfcffb6f41d3_1
AGCTCCAATAGCGTATACTAATGTTGTTGCAGTTAAAAGCTCGTAGTCGGATTTCAGTAGGCTCAATTAAGTCTCTATTCTGAGTTCTTTATGGGCCTGCTTCTTTGACAGAAACTTCTATGTTATTCATTTAGCGTGGGTAGTGACTGTCTCTTTTACTTTGAGAAAATTAGAGTGTTCAAAGCAGGTATTCGCCTGAATATTACTCTTGGAATAATGCTATAAGACTTTGGTTCTAATGTATTGGTGATTGGGACCAGAGTAATGATTGATAGGGACGGTTGGGGTCTTACGTACTGCAAAGCGAGAGGTGAAATTCTTGGACCTTTGTATGACGAACAACTGCGAAAGCATTGGACTAGGACGTTCCCG
>dbf3cc5f6a18572b1c29ca136757ca8eb05647c2_1
AGCTCCAATAGCGTATACTAATGTTGTTGCAGTTAAAAGCTCGTAGTCGGATTTCGGTAGGCTCAATTAAGTCTCTATTCTGAGTTCTTTATGGGCCTGCTTCTTTGACAGAAACTTCTATGTTATTCATTTAGCGTGGGTAGCGACTGTCTCTTTTACTTTGAGAAAATTAGAGTGTTCAAAGCAGGTATTCGCCTGAATATTACTCTTGGAATAATGCTATAAGACTTTGGTTCTAATGTATTGGTGATTGGGACCAGAGTAATGATTGATAGGGACGGTTGGGGTCTTACGTACTGCAAAGCGAGAGGTGAAATTCTTGGACCTTTGTATGACGAACAACTGCGAAAGCATTGGACTAGGACGTTCCCG
>dd835589d6348fb604c08da9a0ef0ec945ca4d8d_1
AGCTCCAATAGCGTATACTAATGTTGTTGCAGTTAAAAGCTCGTAGTCGGATTTCAGTAGGCTCAATTAAGTCTCTATTCTGAGTTCTTTATGGGCCTGCTTCTTTGACAGAAACTTCTATGTTATTCATTTAGCGTGGGTAGCGACTGTCTCTTTTACTTTGAGAAAATTAGAGTGTTCAAAGCAGGTATTCGCCTGAATATTACTCTTGGAATAATGCTATAAGACTTTGGTTCTAATGTATTGGTGATTGGGACCAGAGTAATGATTGATAGGGACGGTTGGGTCGTTACGTACTGCAAAGCGAGAGGTGAAATTCTTGGACCTTTGTATGACGAACAACTGCGAAAGCATTGGACTAGGACGTTCCCGT
>ed68f415109069fc99c40f7d299fae25fa8523f2_1
AGCTCCAATAGCGTATACTAATGTTGTTGCAGTTAAAAGTACTCGTAGTCGGATTTCAGTAGGCTCAATTAAGTCTCTATTCTGAGTTCTTTATGGGCCTGCTTCTTTGACAGAAACTTCTATGTTATTCATTTAGCGTGGGTAGCGACTGTCTCTTTTACTTTGAGAAAATTAGAGTGTTCAAAGCAGGTATTCGCCTGAATATTACTCTTGGAATAATGCTATAAGACTTTGGTTCTAATGTATTGGTGATTGGGACCAGAGTAATGATTGATAGGGACGGTTGGGGTCTTACGTACTGCAAAGCGAGAGGTGAAATTCTTGGACCTTTGTATGACGAACAACTGCGAAAGCATTGGACTAGGACGTTCCCG
>f5c7aff3c08337b05c312a9f338ef5ab9557365b_1
AGCTCCAATAGCGTATACTAATGTTGTTGCAGTTAAAAGCTCGTAGTCGGATTTCGGTAGGCTCAATTAAGTCTCTATTCTGAGTTCTTTATGGGCCTGCTTCTTTGACAGAAACTTCTATGTTATTCATTTAGCGTGGGTAGCGACTGTCTCTTTTACTTTGAGAAAATTAGAGTGTTCAAAGCAGGTATTCGCCTGAATATTACTCTTGGAATAATGCTATAAGACTTTGGTTCTAATGTATTGGTGATTGGGACCAGAGTAATGATTGATAGGGACGGTTGGGGTCTTACGTACTGCAAAGCGAGAGGTGAAATTCTTGGACCTTTGTATGACGAACAACTGCGAAGCATTGGACTAGGACGTTCCCG
>fcd68765ff438bfea35de189753081137e10844e_1
AGCTCCAATAGCGTATACTAATGTTGTTGCAGTTAAAAGCTCGTAGTCGGATTTCAGTAGGCTCAATTAAGTCTCTATTCTGAGTTCTTTATGGGCCTGCTTCTTTGACAGAAACTTCTATGTTATTCATTTAGCGTGGGTAGCGACTGTCTCTTTTACTTTGAGAAAATTAGAGTGTTCAAAGCAGGTATTCGCCTGAATATTACTCTTGGAATAATGCTATAAGACTTTGGTTCTAATGTATTGGTGATTGGGACCAGAGTAATGATTGATAGGGGACGGTTGGGTCTTACGTACTGCAAACGAGAGGTGAAATTCTTGGACCTTTGTATGACGAACAACTGCGAAAGCATTGGACTAGGACGTTCCCG

# Ei44_2_V9_radiolaria.fas
>599a9d1ab5ed3e477599254458c741b14dac1691_926
GTCGCTCCTACCGATTGGATGATTCGGTAAGCTTTTGGGATTGATTGCGTATTTCTCATTGAGGGTACGTTAAAACAACTTAATCAAACCTAATCATCTAGAGGAAGGAGAAGTCGTAACAAGGTTTCC
>f85f078e9dc0ab7aa71e9d679471f24ea11a4045_44
GTCGCTCCTACCGATTGGATGATTCGGTAAGCTTTTGGGTATTGATTGCGTATTTCTCATTGAGGGTACGTTAAAACAACTTAATCAAACCTAATCATCTAGAGGAAGGAGAAGTCGTAACAAGGTTTCC
>ffdd6b43a06e827c764c0b8916ee3d58a3663928_13
GTCGCTCCTACCGATTGGATGATTCGGTAAGCTTTTGGGATTGATTAGCTACTTGTCACTGACATTAGCTTTTAACAACTTAATCAAACCTAATCATCTAGAGGAAGGAGAAGTCGTAACAAGGTTTCC
>445263f7075aca9827657908c184c4d4c45d7579_7
GTCGCTCCTACCGATTGGATGATTCGGTAAATTTTTGGGATTGATTGCGTATTTCTCATTGAGGGTACGTTAAAACAACTTAATCAAACCTAATCATCTAGAGGAAGAAGAAGTCGTAACAAGGTTTCC
>70d22f8cd0a37c4b89d504d500203c942047b8be_3
GTCGTTCCTACCGATTGGATGATTCGGTAACCTTTGGGATTGATTGCGTATTTCTCATTGAGGGTACGTTAAAACAACTTAATCAAACCTAATCATCTAGAGGAAGGAGAAGTCGTAACAAGGTTTCC
>a1adae0fc28fb7a001747456c63c74ddebfa2343_3
GTCGTTCCTACCGATTGGATGATTCGGTAACCTTTTGGGATTGATTGCGTATTTCTCATTGAGGGTACGTTAAAACAACTTAATCAAACCTAATCATCTAGAGGAAGGAGAAGTCGTAACAAGGTTTCC
>40931cf9dd4e2202a429148bbda8d75ade0ec141_1
GTCGCTCCTACCGATTGGATGATTCGGTAAGCTTTTGGGATTGATTGCGTATTTCTCATTGAGGGTACGTTAAAACAACTTAATCAAACCTAATCATCTAGAGGAAGTAAAGTCGTAACAAGGTTTCT
>651d6d95240436d1a30430347469f6ebfcaa9a3a_1
GTCGCTACTACCGATTGGATGGTTCGGTAAGCTTTTGGGTATTGATTGCGTATTTCTCATTGAGGGTACGTTAAAACAACTTAATCAAACCTAATCATCTAGAGGAAGGAGAAGTCGTAACAAGGTTTCC
>76933c57caa9c7f5ab19ba24e0bd029e70f1bcd5_1
GTCGCTCCTACCGATTGAATGATTCGGTAAGCATTCAGGATCTGGTTTTATCTTCCCTTGCGGGAAGATGATCTAGAGAATTTATTCAAACCTAATCATTTAGAGGAAGGAGAAGTCGTAACAAGGTTTCC
>a3293d25c7f244626d4bb91f73eb629b94c998f6_1
GTCGCTCCTACCGATTGGATGATTCGGTAAGCTTTTGGGATTGATTGCGTATTTCTCATTGAGGGTACGTTAAAACAACTTAATCAAACCTAATCATCTAGTGGAAGGAGAAGTCGTAACAAGGTTTCC
>d015ec81ec5730a976593e98cd4c24affd019eb3_1
GTCGCTACTACCGATTGAATGATTCGGTAAGCATTCAGGATCTGGTTTTATCTTCCCTTGCGGGAAGATGATCTAGAGAATTTATTCAAACCTAATCATTTAGAGGAAGGAGAAGTCGTAACAAGGTTTCC

# Ei45_V4_radiolaria.fas
>4a8d393990440970e43450e7b708ebb833101309_297
AGCTCCAATAGCGTATACTAATGTTGTTGCAGTTAAAAGCTCGTAGTCGGATTTCAGTAGGCTCAATTAAGTCTCTATTCTGAGTTCTTTATGGGCCTGCTTCTTTGACAGAAACTTCTATGTTATTCATTTAGCGTGGGTAGCGACTGTCTCTTTTACTTTGAGAAAATTAGAGTGTTCAAAGCAGGTATTCGCCTGAATATTACTCTTGGAATAATGCTATAAGACTTTGGTTCTAATGTATTGGTGATTGGGACCAGAGTAATGATTGATAGGGACGGTTGGGGTCTTACGTACTGCAAAGCGAGAGGTGAAATTCTTGGACCTTTGTATGCGAACAACTGCGAAAGCATTGGACTAGGACGTTCCCG
>21913d145a1652382c8b4d0b53c556f436b04519_251
AGCTCCAATAGCGTATACTAATGTTGTTGCAGTTAAAAAGCTCGTAGTCGGATTTCAGTAGGCTCAATTAAGTCTCTATTCTGAGTTCTTTATGGGCCTGCTTCTTTGACAGAAACTTCTATGTTATTCATTTAGCGTGGGTAGCGACTGTCTCTTTTACTTTGAGAAAATTAGAGTGTTCAAAGCAGGTATTCGCCTGAATATTACTCTTGGAATAATGCTATAAGACTTTGGTTCTAATGTATTGGTGATTGGGACCAGAGTAATGATTGATAGGGACGGTTGGGGTCTTACGTACTGCAAAGCGAGAGGTGAAATTCTTGGACCTTTGTATGACGAACAACTGCGAAAGCATTGGACTAGGACGTTCCCG
>2aa596477e481a5c24ec1af501ea447585efe383_148
AGCTCCAATAGCGTATACTAATGTTGTTGCAGTTAAAAGCTCGTAGTCGGATTTCAGTAGGCTCAATTAAGTCTCTATTCTGAGTTCTTTATGGGCCTGCTTCTTTGACAGAAACTTCTATGTTATTCATTTAGCGTGGGTAGCGACTGTCTCTTTTACTTTGAGAAAATTAGAGTGTTCAAAGCAGGTATTCGCCTGAATATTACTCTTGGAATAATGCTATAAGACTTTGGTTCTAATGTATTGGTGATTGGGACCAGAGTAATGATTGATAGGGACGGTTGGGGTCTTACGTACTGCAAAGCGAGAGGTGAAATTCTTGGACCTTTGTATGACGAACAACTGCGAAAGCATTGGACTAGGACGTTCCCG
>2c0c6774ece834a446910303b7d6a89061530852_25
AGCTCCAATAGCGTATACTAATGTTGTTGCAGTTAAAAAGCTCGTAGTCGGATTTCAGTAGGCTCAGTTAAGTCTCACTTATGAGTTCTTTATGGGCCTGCTTCTTTGACAGAAACTTCTATGTTATTCATTTAGCGTGGGTAGCGACTGTCTCTTTTACTTTGAGAAAATTAGAGTGTTCAAAGCAGGTATTCGCCTGAATATTACTCTTGGAATAATGCTATAAGACTTTGGTTCTAATGTATTGGTGATTGGGACCAGAGTAATGATTGATAGGGACGGTTGGGGTCTTACGTACTGCAAAGCGAGAGGTGAAATTCTTGGACCTTTGTATGACGAACAACTGCGAAAGCATTGGACTAGGACGTTCCCG
>fcdfafc671782f35d589f9b174458f6091155246_23
AGCTCCAATAGCGTATACTAATGTTGTTGCAGTTAAAAGCTCGTAGTCGGATTTCAGTAGGCTCAGTTAAGTCTCACTTATGAGTTCTTTATGGGCCTGCTTCTTTGACAGAAACTTCTATGTTATTCATTTAGCGTGGGTAGCGACTGTCTCTTTTACTTTGAGAAAATTAGAGTGTTCAAAGCAGGTATTCGCCTGAATATTACTCTTGGAATAATGCTATAAGACTTTGGTTCTAATGTATTGGTGATTGGGACCAGAGTAATGATTGATAGGGACGGTTGGGGTCTTACGTACTGCAAAGCGAGAGGTGAAATTCTTGGACCTTTGTATGACGAACAACTGCGAAAGCATTGGACTAGGACGTTCCCG
>2786563d82438b9a3860f7c2d460a7837a0f6790_21
CAGCTCCAATAGCGTATACTAATGTTGTTGCAGTTAAAAAGCTCGTAGTCGGATTTCAGTAGGCTCAATTAAGTCTCTATTCTGAGTTCTTTATGGGCCTGCTTCTTTGACAGAAACTTCTATGTTATTCATTTAGCGTGGGTAGCGACTGTCTCTTTTACTTTGAGAAAATTAGAGTGTTCAAAGCAGGTATTCGCCTGAATATTACTCTTGGAATAATGCTATAAGACTTTGGTTCTAATGTATTGGTGATTGGGACCAGAGTAATGATTGATAGGGACGGTTGGGGTCTTACGTACTGCAAAGCGAGAGGTGAAATTCTTGGACCTTTGTATGACGAACAACTGCGAAAGCATTGGACTAGGACGTTCCCG
>6f9c540de7d0f13f599b6c22273c4ab50cef82ed_21
AGCTCCAATAGCGTATACTAATGTTGTTGCAGTTAAAAGCTCGTAGTCGGATTTCAGTAGGCTCAGTTAAGTCTCACTTATGAGTTCTTTATGGGCCTGCTTCTTTGACAGAAACTTCTATGTTATTCATTTAGCGTGGGTAGCGACTGTCTCTTTTACTTTGAGAAAATTAGAGTGTTCAAAGCAGGTATTCGCCTGAATATTACTCTTGGAATAATGCTATAAGACTTTGGTTCTAATGTATTGGTGATTGGGACCAGAGTAATGATTGATAGGGACGGTTGGGGTCTTACGTACTGCAAAGCGAGAGGTGAAATTCTTGGACCTTTGTATGCGAACAACTGCGAAAGCATTGGACTAGGACGTTCCCG
>61d10cd9d8901559436b26ed0b24f9efdc6ca8fc_17
AGCTCCAATAGCGTATACTAATGTTGTTGCAGTTAAAAAGCTCGTAGTCGGATTTCAGTAGGCTCAATTAAGTCTCTATTCTGAGTTCTTTATGGGCCTGCTTCTTTGACAGAAACTTCTATGTTATTCATTTAGCGTGGGTAGCGACTGTCTCTTTTACTTTGAGAAAATTAGAGTGTTCAAAGCAGGTATTCGCCTGAATATTACTCTTGGAATAATGCTATAAGACTTTGGTTCTAATGTATTGGTGATTGGGACCAGAGTAATGATTGATAGGGACGGTTGGGGTCTTACGTACTGCAAAGCGAGAGGTGAAATTCTTGGACCTTTGTATGACGAACAACTGCGAAAGCATTTGGACTAGGACGTTCCCG
>88fba6969d6b85f03dcbcc34524ca54c4e91c90a_7
CAGCTCCAATAGCGTATACTAATGTTGTTGCAGTTAAAAAGCTCGTAGTCGGATTTCAGTAGGCTCAATTAAGTCTCTATTCTGAGTTCTTTATGGGCCTGCTTCTTTGACAGAAACTTCTATGTTATTCATTTAGCGTGGGTAGCGACTGTCTCTTTTACTTTGAGAAAATTAGAGTGTTCAAAGCAGGTATTCGCCTGAATATTACTCTTGGAATAATGCTATAAGACTTTGGTTCTAATGTATTGGTGATTGGGACCAGAGTAATGATTGATAGGGACGGTTGGGGTCTTACGTACTGCAAAGCGAGAGGTGAAATTCTTGGACCTTTGTATGACGAACAACTGCGAAAGCATTTGGACTAGGACGTTCCCG
>e99dbd8b604fa64617030c9ce29d326825dd3461_6
AGCTCCAATAGCGTATACTAATGTTGTTGCAGTTAAAAAGCTCGTAGTCGGATTTCAGTAGGCTCAATTAAGTCTCTATTCTGAGTTCTTTATGGGCCTGCTTCTTTGACAGAAACTTCTATGTTATTCATTTAGCGTGGGTAGCGACTGTCTCTTTTACTTTGAGAAAATTAGAGTGTTCAAAGCAGGTATTCGCCTGAATATTACTCTTGGAATAATGCTATAAGACTTTGGTTCTAATGTATTGGTGATTGGGACCAGAGTAATGATTGATAGGGACGTTGGGGTCTTACGTACTGCAAAGCGAGAGGTGAAATTCTTGGACCTTTGTATGACGAACAACTGCGAAAGCATTGGACTAGGACGTTCCCG
>02659063e3d82d98e7f05cc3fe2626606f25dc09_5
AGCTCCAATAGCGTATACTAATGTTGTTGCAGTTAAAAGACTCGTAGTCGGATTTCAGTAGGCTCAATTAAGTCTCTATTCTGAGTTCTTTATGGGCCTGCTTCTTTGACAGAAACTTCTATGTTATTCATTTAGCGTGGGTAGCGACTGTCTCTTTTACTTTGAGAAAATTAGAGTGTTCAAAGCAGGTATTCGCCTGAATATTACTCTTGGAATAATGCTATAAGACTTTGGTTCTAATGTATTGGTGATTGGGACCAGAGTAATGATTGATAGGGACGGTTGGGGTCTTACGTACTGCAAAGCGAGAGGTGAAATTCTTGGACCTTTGTATGCGAACAACTGCGAAAGCATTGGACTAGGACGTTCCCG
>1be21b955b3f30da90b6efc64df645f2c352e759_5
AGCTCCAATAGCGTATACTAATGTTGTTGCAGTTAAAAGCTCGTAGTCGGATTTCAGTAGGCTCAGTTTAGTCTTCATTGTAAGATCTATATGGGCCTGCTTCTTTGACAGAAACTTCTATGTTATTCATTTAGCGTGGGCAGCGACTGTCTCTTTTACTTTGAGAAAATTAGAGTGTTCAAAGCAGGTAGATGCCTGAATATTACTCTTGGAATAATGCTATAAGACTTTGGTTCTAATGTATTGGTGATTGGGACCAGAGTAATGATTGATAGGGACGGTTGGGGTCTTACGTACTGCAAAGCGAGAGGTGAAATTCTTGGACCTTTGTATGACGAACAACTGCGAAAGCATTGGACTAGGACGTTCCCG
>2e8afe09212b9024a6c71bf171d9ba5c1b21cca6_4
AGCTCCAATAGCGTATACTAATGTTGTTGCAGTTAAAAGCTCGTAGTCGGATTTCAGTAGGCTCAATTAAGTCTCTATTCTGAGTTCTTTATGGGCCTGCTTCTTTGACAGAAACTTCTATGTTATTCATTTAGCGTGGGTAGCGACTGTCTCTTTTACTTTGAGAAAATTAGAGTGTTCAAAGCAGGTATTCGCCTGAATATTACTCTTGGAATAATGCTATAAGACTTTGGTTCTAATGTATTGGTGATTGGGACCAGAGTAATGATTGATAGGGACGGTTGGGTCGTTACGTACTGCAAAGCGAGAGGTGAAATTCTTGGACCTTTGTATGCGAACAACTGCGAAAGCATTGGACTAGGACGTTCCCG
>93b410ad96937a1d09c69fb102b002f22a5ad22c_4
AGCTCCAATAGCGTATATTTAAGTTGTTGCAGTTAAAAGCTCGTAGTCGGATTTCAGTAGGCTCAATTAAGTCTCTATTCTGAGTTCTTTATGGGCCTGCTTCTTTGACAGAAACTTCTATGTTATTCATTTAGCGTGGGTAGCGACTGTCTCTTTTACTTTGAGAAAATTAGAGTGTTCAAAGCAGGTATTCGCCTGAATATTACTCTTGGAATAATGCTATAAGACTTTGGTTCTAATGTATTGGTGATTGGGACCAGAGTAATGATTGATAGGGACGGTTGGGGTCTTACGTACTGCAAAGCGAGAGGTGAAATTCTTGGACCTTTGTATGACGACAACTGCGAAAGCATTGGACTAGGACGTTCCCG
>69ff08404bef345949031a90782bcb5dccf5240d_3
AGCTCCAATAGCGTATACTAATGTTGTTGCAGTTAAAAAGCTCGTAGTCGGATTTCAGTAGGCTCAATTAAGTCTCTATTCTGAGTTCTTTATGGGCCTGCTTCTTTGACAGAAACTTCTATGTTATTCATTTAGCGTGGGTAGCGACTGTCTCTTTTACTTTGAGAAAATTAGAGTGTTCAAAGCAGGTATTCGCCTGAATATTACTCTTGGAATAATGCTATAAGACTTTGGTTCTAATGTATTGGTGATTGGGACCAGAGTAATGATTGATAGGGACGGTTGGGGTATTACGTACTGCAAAGCGAGAGGTGAAATTCTTGGACCTTTGTATGACGAACAACTGCGAAAGCATTGGACTAGGACGTTCCCG
>2374f28cc21d1b96142733d6fb4d8ff7946b4da5_2
AGCTCCAATAGCGTATACTAATGTTGTTGCAGTTAAAAGCTCGTAGTCGGATTTCAGTAGGCTCAATTAAGTCTCTATTGTGAGTTCTTTATGGGCCTGCTTCTTTGACATAAACTTCTATGTTATTCATTTAGCGTGGGTAGCGACTGTCTCTTTTACTTTGAGAAAATTAGAGTGTTCAAAGCAGGTATTCGCCTGAATATTACTCTTGGAATAATGCTATAAGACTTTGGTTCTAATGTATTGGTGATTGGGACCAGAGTAATGATTGATAGGGACGGTTGGGGTCTTACGTACTGCAAAGCGAGAGGTGAAATTCTTGGACCTTTGTATGCGAACAACTTCGAAAGCATTGGACTAGGACGTTCCCG
>2fd3b7840281f955b86f4aad60ba0ec46e9e1997_2
AGCTCCAATAGCGTATACTAATGTTGTTGCAGTTAAAAAGCTCGTAGTCGGATTTCAGTAGGCTCAATTAAGTCTCTATTCTGAGTTCTTTATGGGCCTGCTTCTTTGACAGAAACTTCTATGTTATTCATTTAGCGTGGGTAGCGACTGTCTCTTTTACTTTGAGAAAATTAGAGTGTTCAAAGCAGGTATTCGCCTGAATATTACTCTTGGAATAATGCTATAAGACTTTGGTTCTAATGTATTGGTGATTGGGACCAGAGTAATGATTGATAGGGACGTTGGGGTCTTACGTACTGCAAAGCGAGAGGTGAAATTCTTGGACCTTTGTATGACGAACAACTGCGAAAGCATTTGGACTAGGACGTTCCCG
>92fed630f16a78df5f55188f0c84082d41fb26db_2
CAGCTCCAATAGCGTATACTAATGTTGTTGCAGTTAAAAGCTCGTAGTCGGATTTCAGTAGGCTCAATTAAGTCTCTATTCTGAGTTCTTTATGGGCCTGCTTCTTTGACAGAAACTTCTATGTTATTCATTTAGCGTGGGTAGCGACTGTCTCTTTTACTTTGAGAAAATTAGAGTGTTCAAAGCAGGTATTCGCCTGAATATTACTCTTGGAATAATGCTATAAGACTTTGGTTCTAATGTATTGGTGATTGGGACCAGAGTAATGATTGATAGGGACGGTTGGGGTCTTACGTACTGCAAAGCGAGAGGTGAAATTCTTGGACCTTTGTATGACGAACAACTGCGAAAGCATTGGACTAGGACGTTCCCG
>aeafb08ac447cf87bd4957205f8db0616fc407d4_2
AGCTCCAATAGCGTATACTAATGTTGTTGCAGTTAAAAAGCTCGTAGTCGGATTTCAGTAGGCTCAGTTAAGTCTCACTTATGAGTTCTTTATGGGCCTGCTTCTTTGACAGAAACTTCTATGTTATTCATTTAGCGTGGGTAGCGACTGTCTCTTTTACTTTGAGAAAATTAGAGTGTTCAGAGCAGGTATTCGCCTGAATATTACTCTTGGAATAATGCTATAAGACTTTGGTTCTAATGTATTGGTGATTGGGACCAGAGTAATGATTGATAGGGACGGTTGGGGTCTTACGTACTGCAAAGCGAGAGGTGAAATTCTTGGACCTTTGTATGACGAACAACTGCGAAAGCATTGGACTAGGACGTTCCCG
>c0b4524118d1696de8870d887e54a3f2494dc005_2
AGCTCCAATAGCGTATACTAATGTTGTTGCAGTTAAAAGCTCGTAGTCGGATTTCAGTAGACTCAATTAAGTCTCTATTCTGAGTTCTTTATGGGCCTGCTTCTTTGACAGAAACTTCTATGTTATTCATTTAGCGTGGGTAGCGACTGTCTCTTTTACTTTGAGAAAATTAGAGTGTTCAAAGCAGGTATTCGCCTGAATATTACTCTTGGAATAATGCTATAAGACTTTGGTTCTAATGTATTGGTGATTGGGACCAGAGTAATGATTGATAGGGACGGTTGGGGTCTTACGTACTGCAAAGCGAGAGGTGAAATTCTTGGACCTTTGTATGCGAACAACTGCGAAAGCATTGGACTAGGACGTTCCCG
>c769dec6b66dca1a382dcf5fb0eec1798086ea39_2
AGCTCCAATAGCGTATACTAATGTTGTTGCAGTTAAAAAGCTCGTAGTCGGATTTCAGTAGGCTCAATTAAGTCTCTATTCTGAGTTCTTTATGGGCCTGCTTCTTTGACAGAAACTTCTATGTTATTCATTTAGCGTGGGTAGCGACTGTCTCTTTTACTTTGAGAAAATTAGAGTGTTCAAAGCAGGTATTCGCCTGAATATTACTCTTGGAATAATGCTATAAGACTTTGGTTCTAATGTATTGGTGATTGGGACCAGAGTAATGATTGATAGGGACGGTTGGTGTCTTACGTACTGCAAAGCGAGAGGTGAAATTCTTGGACCTTTGTATGACGAACAACTGCGAAAGCATTGGACTAGGACGTTCCCG
>c9bd3b9a1ceb4e7e7ca3e8a96d3c23602d2679c6_2
AGCTCCAATAGCGTATACTAATGTTGTTGCAGTTAAAAGCTCGTAGTCGGATTTCAGTAGGCTCAATTAAGTCTCTATTCTGAGTTCTTTATGGGCCTGCTTCTTTGACAGAAACTTCTATGTTATTCATTTAGCGTGGGTAGCGACTGTCTCTTTTACTTTGAGAAAATTAGAGTGTTCAAAGCAGGTATTCGCCTGAATATTACTCTTGGAATAATGCTATAAGACTTTGGTTCTAATGTATTGGTGATTGGGACCAGAGTAATGATTGATAGGGACGTTGGGGTCTTACGTACTGCAAAGCGAGAGGTGAAATTCTTGGACCTTTGTATGCGAACAACTGCGAAAGCATTGGACTAGGACGTTCCCG
>ddadfd454cdb1ea6ecd1647e4f08df92324dc5c4_2
AGCTCCAATAGCGTATACTAATGTTGTTGCAGTTAAAAGCTCGTAGTCGGATTTCAGTAGGCTCAGTTTAGTCTTCATTGTAAGATCTATATGGGCCTGCTTCTTTGACAGAAACTTCTATGTTATTCATTTAGCGTGGGCAGCGACTGTCTCTTTTACTTGAGAAAATTAGAGTGTTCAAAGCAGGTAGATGCCTGAATATTACTCTTGGAATAATGCTATAAGACTTTGGTTCTAATGTATTGGTGATTGGGACCAGAGTAATGATTGATAGGGACGGTTGGGGTCTTACGTACTGCAAAGCGAGAGGTGAAATTCTTGGACCTTTGTATGACGAACAACTGCGAAAGCATTGGACTAGGACGTTCCCG
>f5b3def689b76d00d2c10c3bf781c8900db130e7_2
AGCTCCAATAGCGTATACTAATGTTGTTGCAGTTAAAAACTCGTAGTCGGATTTCAGTAGGCTCAGTTAAGTCTCTATTCTGAGTTCTTTATGGGCCTGCTTCTTTGACAGAAACTTCTATGTTATTCATTTAGCGTGGGTAACGACTGTCTCTTTTACTTTGAGAAAATTAGAGTGTTCAAAGCAGGTATTCGCCTGAATATTACTCTTGGAATAATGCTATAAGACTTTGGTTCTAATGTATTGGTGATTGGGACCAGAGTAATGATTGATAGGGACGGTTGGGGTCTTACGTACTGCAAAGCGAGAGGTGAAATTCTTGGACCTTTGTATGACGAACACTGCGAAAGCATTGGACTAGGACGTTCCCG
>011f9990cb70ad68a0a73614077a004fc74ed58c_1
AGCTCCAATAGCGTATACTAATGTTGTTGCAGTTAAAAAGCTCGTAGTCGGATTTCAGTAGGCTCAATTAAGTCTCTATTCTGAGTTCTTTATGGGCCTGCTTCTTTGACAGAAACTTCTATGTTATTCATTTAGTGTGGGTAGCGACTGTCTCTTTTACTTTGAGAAAATTAGAGTGTTCAAAGCAGGTATTCGCCTGAATATTACTCTTGGAATAATGCTATAAGACTTTGGTTCTAATGTATTGGTGATTGGGACCAGAGTAATGATTGATAGGGACGGTTGGGGTCTTACGTACTGCAAAGCGAGAGGTGAAATTCTTGGACCTTTGTATGACGAACAACTGCGAAAGCATTGGACTAGGACGTTCCCG
>02f6949134059ee06cef42d524a4cabfd9196f41_1
AGCTCCAATAGCGTATACTAATGTTGTTGCAGTTAAAAGCTCGTAGTCGGATTTCAGTAGGCTCAATTAAGTCTCTATTCTGAGTTCTTTATGGGCCTGCTTCTTTGACAGAAACTTCTATGTTATTCATTTAGTGTGGGTAGCGACTGTCTCTTTTACTTTGAGAAAATTAGAGTGTTCAAAGCAGGTATTCGCCTGAATATTACTCTTGGAATAATGCTATAAGACTTTGGTTCTAATGTATTGGTGATTGGGACCAGAGTAATGATTGATAGGGACGGTTGGGGTCTTACGTACTGCAAAGCGAGAGGTGAAATTCTTGGACCTTTGTATGACGAACAACTGCGAAAGCATTGGACTAGGACGTTCCCG
>03aed583c6d86d740497872a4ec2856b0eaa3aa3_1
AGCTCCAATAGCGTATACTAATGTTGTTGCAGTTAAAAAGCTCGTAGTCGGATTTCAGTAGGCTCAATTAAGTCTCTATTGTGAGTTCTTTATGGGCCTGCTTCTTTGACAGAAACTTCTATGTTATTCATTTAGCGTGGGTAGCGACTGTCTCTTTTACTTTGAGAAAATTAGAGTGTTCAAAGCAGGTATTCGCCTGAATATTACTCTTGGAATAATGCTATAAGACTTTGGTTCTAATGTATTGGTGATTGGGACCAGAGTAATGATTGATGGGGACGGTTGGGGTCTTACGTACTGCAAAGCGAGAGGTGAAATTCTTGGACCTTTGTATGACGAACAACTTCGAAAGCATTGGACTAGGACGTTCCCG
>06782282b9ba082956e8f37f85aa5e039f10825c_1
AGCTCCAATAGCGTATACTAATGTTGTTGCAGTTAAAAGCTCGTAGTCGGATTTCAGTAGGCTCAATTAAGTCTCTATTCTGAGTTCTTTATGGGCCTGCTTCTTTGACAGAAACTTCTATGTTATTCATTTAGCGTGGGTAGCGACTGTCTCTTTTACTTTGATAAAATTAGAGTGTTCAAAGCAGGTATTCGCCTGAATATTACTCTTGGAATAATGCTATAAGACTTTGGTTCTAATGTATTGGTGATTGGGACCAGAGTAATGATTGATAGGGACGGTTGGGGTCTTACGTACTGCAAAGCGAGAGGTGAAATTCTTGGACCTTTGTATGACGAACAACTGCGAAAGCATTGGACTAGGACGTTCCCG
>08dc30d35061e550a4db73a6ab79d023bd636edd_1
AGCTCCAATAGCGTATACTAATGTTGTTGCAGTTAAAAAGCTCGTAGTCGGATTTCAGTAGGCTCAATTAAGTCTCTATTCTGAGTTCTTTATGGGCCTGCTTCTTTGACAGAAACTTCTATGTTATTCATTTAGCGTGGGTAGCGACTGTCTCTTTTACTTTGAGAAAATTAGAGTGTTCAAAGCAGGTATTCGCCTGAATATTACTCTTGGAATAATGCTATAAGACTTTGGTTCTAATGTATTGGTGATTGGGACCAGAGTAATGATTGATAGGGACGGTTGGGGTCTTACGTACTGCAAAGCGAGAGGTGAAATTCTTGGACCTTTGTATGACGAACAACTGCGAAAGCATTTGCCAAGGATGTTTTTCA
>16b9221035db2dd7bf993b89e030fa178f4f88e7_1
AGCTCCAATAGCGTATACTAATGTTGTTGCAGTTAAAAAGCTCGTAGTCGGATTTCAGTAGGCTCAGTTTAGTCTTCATTGTAAGATCTATATGGGCCTGCTTCTTTGACAGAAACTTCTATGTTATTCATTTAGCGTGGGGCAGCGACTGTCTCTTTTACTTTGAGAAAATTAGAGTGTTCAAAGCAGGTAGATGCCTGAATATTACTCTTGGAATAATGCTATAAGACTTTGGTTCTAATGTATTGGTGATTGGGACCAGAGTAATGATTGATAGGGACGGTTGGGGTCTTACGTACTGCAAAGCGAGAGGTGAAATTCTTGGACCTTTGTATGACGAACAACTGCGAAAGCATTGGACTAGGACGTTCCCG
>18cd843e317a4a3443c62f288e5df0c35a0a13b0_1
CAGCTCCAATAGCGTATACTAATGTTGTTGCAGTTAAAAGCTCGTAGTCGGATTTCAGTAGGCTCAATTAAGTCTCTATTCTGAGTTCTTTATGGGCCTGCTTCTTTGACAGAAACTTCTATGTTATTCATTTAGCGTGGGTAGCGACTGTCTCTTTTACTTTGAGAAAATTAGAGTGTTCAAAGCAGGTATTCGCCTGAATATTACTCTTGGAATAATGCTATAAGACTTTGGTTCTAATGTATTGGTGATTGGGACCAGAGTAATGATTGATAGGGACGGTTGGGGTCTTACGTACTGCAAAGCGAGAGGTGAAATTCTTGGACCTTTGTATGCGAACAACTGCGAAAGCATTGGACTAGGACGTTCCCG
>1a94692ba37be6370a00cbaa09d7582266da4575_1
CAGCTCCAATAGCGTATACTAATGTTGTTGCAGTTAAAAAGCTCGTAGTCGGATTTCAGTAGGCTCAATTAAGTCTCTATTCTGAGTTCTTTATGGGCCTGCTTCTTTGACAGAAACTTCTATGTTATTCATTTAGCGTGGGTAGCGACTGTCTCTTTTACTTTGAGAAAATTAGAGTGTTCAAAGCAGGTATTCGCCTGAATATTACTCTTGGAATAATGCTATAAGACTTTGGTTCTAATGTATTGGTGATTGGGACCAGAGTAATGATTGATAGGGACGGTTGGGGTCTTACGTACTGCAACGCGAGAGGTGAAATTCTTGGACCTTTGTATGACGAACAACTGCGAAAGCATTTGGACTAGGACGTTCCCG
>2262cdd9dfee8aef06d4234d721da7b13b735d8d_1
AGCTCCAATAGCGTATACTAATGTTGTGCAGTTAAAAAGACTCGTAGTCGGATTTCAGTAGGCTCAATTAAGTCTCTATTCTGAGTTCTTTATGGGCCTGCTTCTTTGACAGAAACTTCTATGTTATTCATTTAGCGTGGGTAGCGACTGTCTCTTTTACTTTGAGAAAATTAGAGTGTTCAAAGCAGGTATTCGCCTGAATATTACTCTTGGAATAATGCTATAAGACTTTGGTTCTAATGTATTGGTGATTGGGACCAGAGTAATGATTGATAGGGACGGTTGGGGTCGTTACGTACTGCAAAGCGAGAGGTGAAATTCTTGGACCTTTGTATGACGAACAACTGCGAAAGCATTGGACTAGGACGTTCCCG
>26c6c15fb89cf6589a17a02622941a7094fddc00_1
CAGCTCCAATAGCGTATACTAATGTTGTTGCAGTTAAAAAGCTCGTAGTTGGATTTCAGTAGGCTCAATTAAGTCTCTATTCTGAGTTCTTTATGGGCCTGCTTCTTTGACAGAAACTTCTATGTTATTCATTTAGCGTGGGTAGCGACTGTCTCTTTTACTTTGAGAAAATTAGAGTGTTCAAAGCAGGTATTCGCCTGAATATTACTCTTGGAATAATGCTATAAGACTTTGGTTCTAATGTATTGGTGATTGGGACCAGAGTAATGATTGATAGGGACGGTTGGGGTCTTACGTACTGCAAAGCGAGAGGTGAAATTCTTGGACCTTTGTATGACGAACAACTGCGAAAGCATTTGGACTAGGACGTTCCCG
>29141302c0c6f91a3c610860c6a3082777eb5afb_1
CAGCTCCAATAGCGTATACTAATGTTGTTGCAGTTAAAAAGCTCGTAGTCGGATTTCAGTAGGCTCAGTTAAGTCTCACTTATGAGTTCTTTATGGGCCTGCTTCTTTGACAGAAACTTCTATGTTATTCATTTAGCGTGGGTAGCGACTGTCTCTTTTACTTTGAGAAAATTAGAGTGTTCAAAGCAGGTATTCGCCTGAATATTACTCTTGGAATAATGCTATAAGACTTTGGTTCTAATGTATTGGTGATTGGGACCAGAGTAATGATTGATAGGGACGGTTGGGGTCTTACGTACTGCAAAGCGAGAGGTGAAATTCTTGGACCTTTGTATGACGAACAACTGCGAAAGCATTTGGACTAGGACGTTCCCG
>39d79873cf063ce2efc6bca231ba04f53941e25d_1
AGCTCCAATAGCGTATACTAATGTCGTTGCAGTTAAAAGCTCGTAGTCGGATTTCAGTAGGCTCAGTTTAGTCTTCATTGTAAGATCTATATGGGCCTGCTTCTTTGACAGAAACTTCTATGTTATTCATTTAGCGTGGGCAGCGACTGTCTCTTTTACTTTGAGAAAATTAGAGTGTTCAAAGCAGGTAGATGCCTGAATATTACTCTTGGAATAATGCTATAAGACTTTGGTTCTAATGTATTGGTGATTGGGACCAGAGTAATGATTGATAGGGACGGTTGGGGTCTTACGTACTGCAAAGCGAGAGGTGAAATTCTTGGACCTTTGTATGACGAACAACTGCGAAAGCATTGGACTAGGACGTTCCCG
>3b86c742ed187132c4b26379b126810b401bc2a3_1
AGCTCCAATAGCGTATACTAATGTTGTTGCAGTTAAAAGCTCGTAGTTAGATTTCAGTAGGCTCAATTAAGTCTCTATTGTGAGTTCTTTATGGGCCTGCTTCTTTGACAGAAACTTCTATGTTATTCATTTAGCGTGGGTAGCGACTGTCTCTTTTACTTTGAGAAAATTAGAGTGTTCAAAGCAGGTATTCGCCTGAATATTACTCTTGGAATAATGCTATAAGACTTTGGTTCTAATGTATTGGTGATTGGGACCAGAGTAATGATTGATAGGGACGGTTGGGTCTTACGTACTGCAAAGCGAGAGGTGAAATTCTTGGACCTTTGTATGCGAACAACTTCGAAAGCATTGGACTAGGACGTTCCCG
>41f4941b41c6c05903bc487c286092c59f74cafb_1
AGCTCCAATAGCGTATACTAATGTTGTTGCAGTTAAAAGCTCGTAGTTAGATTTCAGTAGGCTCAATTAAGTCTCTATTGTGAGTTCTTTATGGGCCTGCTTCTTTGACAGAAACTTCTATGTTATTCATTTAGCGTGGGTAGCGACTGTCTCTTTTACTTTGAGAAAATTAGAGTGTTCAAAGCAGGTATTCGCCTGAATATTACTCTTGGAATAATGCTATAAGACTTTGGTTCTAATGTATTGGTGATTGGGACCAGAGTAATGATTGATAGGGACGGTTGGGGTCTTACGTACTGCAAAGCGAGAGGTGAAATTCTTGGACCTTTGTATGCGAACAACTTCGAAAGCATTGGACTAGGACGTTCCCG
>46087bc5bcb6b920cf4ca051758fdd226ec124c0_1
AGCTCCGATAGCGTATACTAATGTTGTTGCAGTTAAAAGCTCGTAGTCGGATTTCAGTAGGCTCAATTAAGTCTCTATTCTGAGTTCTTTATGGGCCTGCTTCTTTGACAGAAACTTCTACGTTATTCATTTAGCGTGGGTAGCGACTGTCTCTTTTACTTTGAGAAAATTAGAGTGTTCAAAGCAGGTATTCGCCTGAATATTACTCTTGGAATAATGCTATAAGACTTTGGTTCTAATGTATTGGTGATTGGGACCAGAGTAATGATTGATAGGGACGGTTGGGGTCTTACGTACTGCAAAGCGAGAGGTGAAATTCTTGGACCTTTGTATGACGACAACTGCGAAAGCATTGGACTAGGACGTTCCCG
>46a73a2f5a999efdb7b3e587032e852f44d44bd9_1
CAGCTCCAATAGCGTATACTAATGTTGTTGCAGTTAAAAAGCTCGTAGTCGGATTTCAGTAGGCTCAATTAAGTCTCTATTCTGAGTTCTTTAATGGGCCTGCTTCTTTGACAGAAACTTCTATGTTATTCATTTAGCATGGGTAGCGACTGTCTCTTTTACTTTGAGAAAATTAGAGTGTTCAAAGCAGGTATTCGCCTGAATATTACTCTTGGAATAATGCTATAAGACTTTGGTTCTAATGTATTGGTGATTGGGACCAGAGTAATGATTGATAGGGACGGTTGGGGTCTTACGTACTGCAAAGCGAGAGGTGAAATTCTTGGACCTTTGTATGACGAACAACTGCGAAAGCATTGGACTAGGACGTTCCCG
>487f9b6da71fc8f47db216d522083c65d744ecb1_1
AGCTCCAATGGTGTATGCTAACATTGTTGCAGTTAAAAAGCTCGTAGTCGAATTTGTAAGAAAATCAATTTTATGTGATCCTAATGAACAAAAAAGATGATTTACCTTATTTTATGCTTATGCACCGTTGAAATTCATTTTTTGACGGTGGACTGATTTTGTGTTTACTTTGAGAAAATTAGAGTGTTCAAAGCAGCCTTTGTGGACTTATATTAAAGCATGGAATGATAAATAATGACATTGGTTAATTTTTGTTGGGATGAGGAGCTGATGTAATGATTTATAGAGTTAGTCGGAGGTATTAGTATTTTATCGTTAGAAGTGAAATTTTTGGATCGTTTAAAGACTACAATTGCAAAAGTATTTACCTAGAATTTTCTCT
>4920f80e6050ee7adeb25207a09a6965cb3b8891_1
AGCTCCAATAGCGTATACTAATGTTGTTGCAGTTAAAAAGCTCGTAGTCGGATTTCAGTAGGCTCAATTAAGTCTCTATTCTGAGTTCTTTATGGGCCTGCTTCTTTGACAGAAACTTCTATGTTATTCATTTAGCGTGGGTAGCGACTGTCTCTTTTACTTTGAGAAAATTAGAGTGTTCAAAGCAGGTATTCGCCTGAATATTACTCTTGGAATAATGCTATAATACTTTGGTTCTAATGTATTGGTGATTGGGACCAGAGTAATGATTGATAGGGACGGTTGGGGTCTTACGTACTGCAAAGCGAGAGGTGAAATTCTTGGACCTTTGTATGACGAACAACTGCGAAAGCATTGGACTAGGACGTTCCCG
>4d04bc619a544d90f2e7b74e364a98cd9fc2fef0_1
AGCTCCAATAGCGTATACTAATGTTGTTGCAGTTAAAAAGCTCGTAGTCGGATTTCAGTAGGCTCAATTAAGTCTCTATTCTGAGTTCTTTATGGGCCTGCTTCTTTGACAGAAACTTCTATGTTATTCATTTAGCGTGGGTAGCGACTGTCTCTTTTACTTTGAGAAAATTAGAGTGTTCAAAGCAGGTATTCGCCTGAATATTACTCTTGGAATAATGCTATAAGACTTTGGTTCTAATGTATTGGTGATTGGGACCAGAGTAATGATTGATAGGGACGTCGGGGTCTTACGTACTGCAAAGCGAGAGGTGAAATTCTTGGACCTTTGTATGACGAACAACTGCGAAAGCATTGGACTAGGACGTTCCCG
>4d8a8ac05559e7c3dd49746795e7b93a1feadff6_1
AGCTCCAATAGCGTATACTAATGTTGTTGCAGTTAAAAGCTCGTAGTCGGATTTCAGTAGGCTCAATTAAGTCTCTATTCTGAGTTCTTTATGGGCCTGCTTCTTTGACAGAAACTTCTATGTTATTCATTTAGCGTGGGTAGCGACTGTCTCTTTTACTTTGAGAAAATTAGAGTGTTCAAAGCAGGTATTCGCCTGAATATTACTCTTGGAATAATGCTATAAGACTTTGGTTCTAATGTATTGGTGATTGGGACCAGAGTAATGATTGATAGGGACGGTTGGGGTCTTACGTACTGCAAAGCGAGAGGTGAAATTCTTGGACCTTTGTATGACGAACAACTGCGAAAGCATTTGGACTAGGACGTTCCCG
>56149e46da8784b50aa73cbc815bc37470864eeb_1
AGCTCCAATAGCGTATACTAATGTTGTTGCAGTTAAAAAGCTCGTAGTCGGATTTCAGTAGGCTCAATTAAGTCTCTATTCTGAGTTCTTTATGGGCCTGCTTCTTTGACAGAAACTTCTATGTTATTCATTTAGCGTGGGTAGCGACTGTCTCTTTTACTTTGAGAAAATTAGAGTGTTCAAAGCAGGTATTCGCCTGAATATTACTCTTGGAATAATGCTATAAGACTTTGGTTCTAATGTATTGGTGATTGGGACCAGAGTAATGATTGATAGGGACGGTTGGGGTCTTACGTACTGCAAAGCGAGAGGTGAAATTCTTGGACCTTTGTACGCGAACAACTGCGAAAGCATTGGACTAGGACGTTCCCG
>5d0b5a2f862a33888c86c4e6b5dbb6e4f3fe8aec_1
AGCTCCAATAGCGTATACTAATGTTGTTGCAGTTAAAAAGCTCGTAGTCGGATTTCAGTAGGCTCAGTTTAGTCTTCATTGTAAGATCTATATGGGCCTGCTTCTTTGACAGAAACTTCTATGTTATTCATTTAGTGTGGGCAGCGACTGTCTCTTTTACTTGAGAAAATTAGAGTGTTCAAAGCAGGTAGATGCCTGAATATTACTCTTGGAATAATGCTATAAGACTTTGGTTCTAATGTATTGGTGATTGGGACCAGAGTAATGATTGATAGGGACGGTTGGGGTCTTACGTACTGCAAAGCGAGAGGTGAAATTCTTGGACCTTTGTATGACGAACAACTGCGAAAGCATTGGACTAGGACGTTCCCG
>651681521e379c440cb789bc2b04d68ca3c2d4d3_1
AGCTCCAATAGCGTATACTAATGTTGTTGCAGTTAAAAAGCTCGTAGTCGGATTTCAGTAGGCTCAATTAAGTCTCTATTGTGAGTTCTTTATGGGCCTGTTTCTTTGACAGAAACTTCTATGTTATTCATTTAGCGTGGGTAGCGACTGTCTCTTTTACTTTGAGAAAATTAGAGTGTTCAAAGCAGGTATTCGCCTGAATATTACTCTTGGAATAATGCTATAAGACTTTGGTTCTAATGTATTGGTGATTGGGACCAGAGTAATGATTGATAGGGACGGTTGGGGTCTTACGTACTGCAAAGCGAGAGGTGAAATTCTTGGACCTTTGTATGCGAACAACTGCGAAAGCATTGGACTAGGACGTTCCCG
>6c2bd9b88b9460383169c67ba7bb22b6e282e00f_1
CAGCTCCAATAGCGTATACTAATGTTGTTGCAGTTAAAAAGCTCGTAGTCGGATTTCAGTAGGCTCAATTAAGTCTCTATTCTGAGTTCTTTATGGGCCTGCTTCTTTGACAGAAACTTCTATGTTATTCATTTAGCGTGGGTAGCGACTGTCTCTTTTACTTTGAGAAAATTAGAGTGTTCAAAGCAGGTATTCGCCTGAATATTACTCTTGGAATAATGCTATAAGACTTTGGTTCTAATGTATTGGTGATTGGGACCAGAGTAATGATTGATAGGGACGTTGGGGTCTTACGTACTGCAAAGCGAGAGGTGAAATTCTTGGACCTTTGTATGACGAACAACTGCGAAAGCATTTGGACTAGGACGTTCCCG
>70ba08a0f37ed16e358f6f44bad529d59968ca93_1
AGCTCCAATAGCGTATACTAATGTTGTTGCAGTTAAAAAGCTCGTAGTCGGATTTCAGTAGGCTCAGTTAAGTCTCACTTATGAGTTCTTTATGGGCCTGCTTCTTTGACAGAAACTTCTATGTTATTCATTTAGCGTGGGTAGCGACTGTCTCTTTTACTTTGAGAAAATTAGAGTGTTCAAAGCAGGTATTCGCCTGAATATTACTCTTGGAATAATGCTATAAGACTTTGGTTCTAATGTATTGGTGATTGGGACCAGAGTAATGATTGATAGGGACGGTTGGGGTCTTACGTACTGCAAAGCGAGAGGTGAAATTCTTGGACCTTTGTATGACGAACAACTACGAAAGCATTGGACTAGGACGTTCCCG
>71e6aa2d7bb421ce4ac3a081786c600ed38e02b5_1
AGCTCCAATAGCGTATACTAATGTTGTTGCAGTTAAAAGGACTCGTAGTCGGATTTCAGTAGGCTCAGTTTAGTCTTCATTGTAAGATCTATATGGGCCTGCTTCTTTGACAGAAACTTCTATGTTATTCATTTAGCGTGGGCAGCGACTGTCTCTTTTTACTTTGAGAAAATTAGAGTGTTCAAAGCAGGTAGATGCCTGAATATTACTCTTGGAATAATGCTATAAGACTTTGGTTCTAATGTATTGGTGATTGGGACCAGAGTAATGATTGATAGGGACGGTTGGGTCTTACGTACTGCAAAGCGAGAGGTGAAATTCTTGGACCTTTGTATGACGAACAACTGCGAAAGCATTGGACTAGGACGTTCCCG
>790e640fe6fdd4d48971f5837c80caaeb9736267_1
AGCTCCAATAGCGTATACTAATGTTGTTGCAGTTAAAAAGCTCGTAGTCGGATTTCAGTAGGCTCAGTTAAGTCTCACTTATGAGTTCTTTATGGGCCTGCTTCTTTGACAGAAACTTCTATGTTATTCATTTAGCGTGGGTAGCGACTGTCTCTTTTACTTTGAGAAAATTAGAGTGTTCAAAGCAGGTATTCGCCTGAATATTACTCTTGGAATAATGATATAAGACTTTGGTTCTAATGTATTGGTGATTGGGACCAGAGTAATGATTGATAGGGACGGTTGGGGTCTTACGTACTGCAAAGCGAGAGGTGAAATTCTTGGACCTTTGTATGACGAACAACTGCGAAAGCATTGGACTAGGACGTTCCCG
>794172d33a2f7f31952cdf9efbc7f6d70cea44f6_1
AGCTCCAATAGCGTATACTAATGTTGTTTGCAGTTAAAAGACTCGTAGTCGGATTTCAGTAGGCTCAATTAAGTCTCTATTCTGAGTTCTTTATGGGCCTGCTTCTTTGACAGAAACTTCTATGTTATTCATTTAGCGTGGGTAGCGACTGTCTCTTTTACTTTGAGAAAATTAGAGTGTTCAAAGCAGGTATTCGCCTGAATATTACTCTTGGAATAATGCTATAAGACTTTGGTTCTAATGTATTGGTGATTGGGACCAGAGTAATGATTGATAGGGACGGTTGGGTCTTACGGTACTGCAAAGCGAGAGGTGAAATTCTTGGACCTTTGTATGCGAACAACTGCGAAAGCATTGGACTAGGACGTTCCCG
>7de5a3479dd1d4bcac646cc20ea102281ef47ed2_1
AGCTCCAATAGCGTATACTAATGTTGTTGCAGTTAAAAGCTCGTAGTACGGATTTCAGTAGGCTCAAATTAAGTCTCTATTCTGAGTTCTTTATGGGCCTGCTTCTTTGACAGAAACTTCTATGTTATTCATTTAGCGTGGGTAGCGACTGTCGTCTTTTACTTTGAGAAAATTAGAGTGTTCAAAGCAGGTATTCGCCTGAATATTACTCTTGGAATAATGCTATAAGACTTTGGTTCTAATGTATTGGTGATTGGGACCAGAGTAATGATTGATAGGGACGATTGGGGTCTTACGTACTGCAAAGCGAGAGGTGAAATTCTTGGACCTTTGTATGACGAACAACTGCGAAAGCATTGGACTAGGACGTTCCCG
>8693acfc1de88a697513943505b97c770288272b_1
AGCTCCAATAGCGTATACTAATGTTGTTGCAGTTAAAAGCTCGTAGTCGGATTTCAGTAGGCTCAATTAAGTCTCTATTCTGAGTTCTTTACGGGCCTGCTTCTTTGACAGAAACTTCTATGTTATTCATTTAGCGTGGGTAGCGACTGTCTCTTTTACTTTGAGAAAATTAGAGTGTTCAAAGCAGGTATTCGCCTGAATATTACTCTTGGAATAATGCTATAAGACTTTGGTTCTAATGTATTGGTGATTGGGACCAGAGTAATGATTGATAGGGACGGTTGGGGTCTTACGTACTGCAAAGCGAGAGGTGAAATTCTTGGACCTTTGTATGACGACAACTGCGAAAGCATTGGACTAGGACGTTCCCG
>8861d58a11f7ca0b38f802cdf81e9ab44a7baab9_1
AGCTCCAATGGTGTATGCTAACATTGTTGCAGTTAAAAAGCTCGTAGTCGAATTTGTAAGAAAATCAATTTTATGTGATCCTAATGAACAAAAAAAGATGATTTACCTTATTTTATGCTTATGCACCGTTGAAATTCATTTTTTGACGGTGGACTGATTTTGTGTTTACTTTGAGAAAATTAGAGTGTTCAAAGCAGCCTTTGTGGACTTATATTAAAGCATGGAATGATAAATAATGACATTGGTTAATTTTTGTTGGGATGAGGAGCTGATGTAATGATTTATAGAGTTAGTCGGAGGTATTAGTATTTTATCGTTAGAAGTGAAATTCTTGGATCGTTTAAAGCTAACAATTGCAAAAGTATTTACCTAGAATTTTCTCT
>8d144e9c76bb100e99b3f60fa327e89a1d3e8e14_1
AGCTCCAATAGCGTATACTAATGTTGTTGCAGTTAAAAGACTCGTAGTCGGATTTCAGTAGGCTCAATTAAGTCTCTATTCTGAGTTCTTTATGGGCCTGCTTCTTTGACAGAAACTTCTATGTTATTCATTTAGCGTGGGTAGCGACTGTCTCTTTTACTTTGAGAAAATTAGAGTGTTCAAAGCAGGTATTCGCCTGAATATTACTCTTGGAATAATGCTATAAGACTTTGGTTCTAATGTATTGGTGATTGGGACCAGAGTAATGATTGATAGGGACGGTTTGGGCGTTACGTACTGCAAAGCGAGAGGTGAAATTCTTGGACCTTTGTATGCGAACAACTGCGAAAGCATTGGACTAGGACGTTCCCG
>970551137516c7565cf4514e09ff23f49e491f0c_1
AGCTCCAATAGCGTATACTAATGTTGTTGCAGTTAAAAAGCTCGTAGTCGGATTTCAGTAGGCTCAATTAAGTCTCTATTCTGAGTTCTTTATGGGCCTGCTTCTTTGACAGAAACTTCTATGTTATTCATTTAGCGTGGGTAGCGACTGTCTCTTTTACTTTGAGAAAATTAGAGTGTTCAAAGCAGGTATTCGCCTCAATATTACTCTTGGAATAATGCTATAAGACTTTGGTTCTAATGTATTGGTGATTGGGACCAGAGTAATGATTGATAGGGACGGTTGGGGTCTTACGTACTGCAAAGCGAGAGGTGAAATTCTTGGACCTTTGTATGACGAACAACTGCGAAAGCATTGGACTAGGACGTTCCCG
>9d3470390b0cc84dc6154fb4e7efa103b0b21b0d_1
AGCTCCAATAGCGTATACTAATGTTGTTGCAGTCAAAAAGCTCGTAGTCGGATTTCAGTAGGCTCAATTAAGTCTCTATTGTGAGTTCTTTATGGGCCTGCTTCTTTGACAGAAACTTCTATGTTATTCATTTAGCGTGGGTAGCGACTGTCTCTTTTACTTTGAGAAAATTAGAGTGTTCAAAGCAGGTATTCGCCTGAATATTACTCTTGGAATAATGCTATAAGACTTTGGTTCTAATGTATTGGTGATTGGGACCAGAGTAATGATTGATAGGGACGGTTGGGGTCTTACGTACTGCAAAGCGAGAGGTGAAATTCTTGGACCTTTGTATGACGAACAACTGCGAAAGCATTTGGACTAGGACGTTCCCG
>9eb763196e689ce1bfa286af174d87e25f4fd41d_1
AGCTCCAATAGCGTATACTAATGTTGTTGCAGTTAAAAGCTCGTAGTCGGATTTCAGTAGGCTCAATTAAGTCTCTATTCTGAGTTCTTTATGGGCCTGCTTCTTTGACAGAAACTTCTATGTTATTCATTTAGCGTGGGTAGCGACTGTCTCTTTTACTTTGAGAAAATTAGAGTGTTCAAAGCAGGTATTCGCCTGAATATTACTCTTGGAATAATGCTATAAGACTTTGGTTCTAATGTATTGGTGATTGGGACCAGAGTAATGATTGATAGGGACGGTTGGGGTCTTACGTACTACAAAGCGAGAGGTGAAATTCTTGGACCTTTGTATGACGACAACTGCGAAAGCATTGGACTAGGACGTTCCCG
>9edd7fb6d0d01ffe12952c17d4158bff40bfebf3_1
AGCTCCAATAGCGTATACTAATGTTGTTGCAGTTAAAAGCTCGTAGTCGGATTTCAGTAGGCTCAATTAAGTCTCTATTGTGAGTTCTTTATGGGCCTGTTTCTTTGACAGAAACTTCTATGTTATTCATTTAGCGTGGGTAGCGACTGTCTCTTTTACTTTGAGAAAATTAGAGTGTTCAAAGCAGGTATTCGCCTGAATATTACTCTTGGAATAATGCTATAAGACTTTGGTTCTAATGTATTGGTGATTGGGACCAGAGTAATGATTGATAGGGACGGTTGGGGTCTTACGTACTGCAAAGCGAGAGGTGAAATTCTTGGACCTTTGTATGACGACAACTTCGAAAGCATTGGACTAGGACGTTCCCG
>a9212629c00b2d9c82abee8f5f8f6c613053f25c_1
AGCTCCAATAGCGTATACTAATGTTGTTGCAGTTAAAAACTCGTAGTCGGATTTCAGTAGGCTCAGTTAAGTCTCTATTCTGAGTTCTTTATGGGCCTGCTTCTTTGACAGAAACTTCTATGTTATTCATTTAGCGTGGGTAACGACTGTCTCTTTTACTTTGAGAAAATTAGAGTGTTCAAAGCAGGTATTCGCCTGAATATTACTCTTGGAATAATGCTATAAGACTTTGGTTCTAATGTATTGGTGATTGGGACCAGAGTAATGATTGATAGGGACGGTTGGGGTCTTACGTACTGCAAAGCGAGAGGTGAAATTCTTGGACCTTTGTATGACGACAACTGCGAAAGCATTGGACTAGGACGTTCCCG
>af511eb31b1e0c1bd4280e461758f663259ab89e_1
AGCTCCAATAGCGTATACTAATGTTGTTGCAGTTAAAAGACTCGTAGTCGGATTTCAGTAGGCTCAATTAAGTCTCTATTCTGAGTTCTTTATGGGCCTGCTTCTTTGACAGAAACTTCTATGTTATTCATTTAGCGTGGGTAGCGACTGTCTCTTTTACTTTGAGAAAATTAGAGTGTTCAAAGCAGGTATTCGCCTGAATATTACTCTTGGAATAATGCTATAAGACTTTGGTTCTAATGTATTGGTGATTGGGACCAGAGTAATGATTGATAGGGACGGTTGGGCTTACGGTACTGCAAAGCGAGAGGTGAAATTCTTGGACCTTTGTATGCGAACAACTGCGAAAGCATTGGACTAGGACGTTCCCG
>afbe509bafd41310e6fa0c3085b3866f347d3d51_1
CAGCTCCAATAGCGTATACTAATGTTGTTGCAGTTAAAAAGCTCGTAGTCGGATTTCAGTAGGCTCAATTAAGTCTCTATTCTGAGTTCTTTATGGGCCTGCTTCTTTGACAGAAACTTCTATGTTATTCATTTAGCGTGGGTAGCGACTGTCTCTTTTACTTTGAGAAAATTAGAGTGTTCAAAGCAGGTATTCGCCTGAATATTACTCTTGGAATAATGCTATAAGACTTTGGTTCTAATGTATTGGTGATTGGGACCAGAGTAATGATTGATAGGGACGGTTGGGGTCTTACGTACTGCAAAGCGAGAGGTGAAATTCTTGGACCTTTGTATGACGAACAAGTGCGAAAGCATTGGACTAGGACGTTCCCG
>ba3cefcc7b668d466038f81d3f40bf8ed72f1f05_1
AGCTCCAATAGCGTATACTAATGTTGTTGCAGTTAAAAGACTCGTAGTCGGATTTCAGTAGGCTCAATTAAGTCTCTATTCTGAGTTCTTTATGGGCCTGCTTCTTTGACAGAAACTTCTATGTTATTCATTTAGCGTGGGTAGCGACTGTCTCTTTTACTTTGAGAAAATTAGAGTGTTCAAAGCAGGTATTCGCCTGAATATTACTCTTGGAATAATGCTATAAGACTTTGGTTCTAATGTATTGGTGATTGGGACCAGAGTAATGATTGATAGGGACGGTTGGGGCTTACGTACTGCAAGCGAGAGGTGAAATTCTTGGACCTTTGTATTGACGAACAACTGCGAAAGCATTGGACTAGGACGTTCCCG
>bd73ec6b78a16562e1ad29174525c67d1e603dd9_1
AGCTCCAATAGCGTATACTAATGTTGTTGCAGTTAAAAAGCTCGTAGTCGGATTTCAGTAGGCTCAATTAAGTCTCTATTCTGAGTTCTTTATGGGCCTGCTTCTTTGACAGAAACTTCTATGTTATTCATTTAGCGTGGGTAGCGACTGTCTCTTTTACTTTGAGAAAATTAGAGTGTTCAAAGCAGGTATTCGCCTGAATATTACTCTTGGAATAATGCTATAAGACTTTGGTTCTAATGTATTGGTGATTGGGACCAGAGTAATGATTGATAGGGACGGTTGGGGTCGTTACGTACTGCAAAGCGAGAGGTGAAATTCTTGGACCTTTGTATGACGAACAACTGCGAAAGCATTGGACTAGGACGTTCCCG
>bd868d9f9a14dd6fe384869ac24b798a57a66eeb_1
CAGCTCCAATAGCGTATACTAATGTTGTTGCAGTTAAAAAGCTCGTAGTCGGATTTCAGTAGGCTCAGTTAAGTCTCACTTATGAGTTCTTTATGGGCCTGCTTCTTTGACAGAAACTTCTATGTTATTCATTTAGCGTGGGTAGCGACTGTCTCTTTTACTTTGAGAAAATTAGAGTGTTCAAAGCAGGTATTCGCCTGAATATTACTCTTGGAATAATGCTATAAGACTTTGGTTCTAATGTATTGGTGATTGGGACCAGAGTAATGATTGATAGGGACGGTTGGGGTCTTACGTACTGCAAAGCGAGAGGTGAAATTCTTGGACCTTTGTATGACGAACAACTGCGAAAGCATTGGACTAGGACGTTCCCG
>cc72624176f59c9ad97301d3893b95d5ac11f354_1
AGCTCCAATAGCGTATACTAATGTTGTTGCAGTTAAAAAGCTCGTAGTCGGATTTCAGTAGGCTCAATTAAGTCTCTATTCTGAGTTCTTTATGGGCCTACTTCTTTGACAGAAACTTCTATGTTATTCATTTAGCGTGGGTAGCGACTGTCTCTTTTACTTTGAGAAAATTAGAGTGTTCAAAGCAGGTATTCGCCTGAATATTACTCTTGGAATAATGCTATAAGACTTTGGTTCTAATGTATTGGTGATTGGGACCAGAGTAATGATTGATAGGGACGGTTGGGGTCTTACGTACTGCAAAGCGAGAGGTGAAATTCTTGGACCTTTGTATGACGAACAACTGCGAAAGCATTGGACTAGGACGTTCCCG
>cd357f2803096aaeb7b8e65488e9ef1b0bc8d688_1
AGCTCCAATAGCGTATACTAATGTTGTTGCAGTTAAAAAGCTCGTAGTCGGATTTCAGTAGGCTCAGTTTAGTCTTCATTGTAAGATCTATATGGGCCTGCTTCTTTGACAGAAACTTCTATGTTATTCATTTAGCGTGGGCAGCGACTGTCTCTTTTACTTTGAGAAAATTAGAGTGTTCAAAGCAGGTAGATGCCTGAATATTACTCTTGGAATAATGCTATAAGACTTTGGTTCTAATGTATTGGTGATTGGGACCAGAGTAATGATTGATAGGGACGGTTGGGGTCTTACGTACTGCAAAGCGAGAGGTGAAATTCTTGGACCTTTGTATGACGAACAACTGCGAAAGCATTGGACTAGGACGTTCCCG
>d1759d87ff72bd59c78876fc61edcfcffb6f41d3_1
AGCTCCAATAGCGTATACTAATGTTGTTGCAGTTAAAAGCTCGTAGTCGGATTTCAGTAGGCTCAATTAAGTCTCTATTCTGAGTTCTTTATGGGCCTGCTTCTTTGACAGAAACTTCTATGTTATTCATTTAGCGTGGGTAGTGACTGTCTCTTTTACTTTGAGAAAATTAGAGTGTTCAAAGCAGGTATTCGCCTGAATATTACTCTTGGAATAATGCTATAAGACTTTGGTTCTAATGTATTGGTGATTGGGACCAGAGTAATGATTGATAGGGACGGTTGGGGTCTTACGTACTGCAAAGCGAGAGGTGAAATTCTTGGACCTTTGTATGACGAACAACTGCGAAAGCATTGGACTAGGACGTTCCCG
>d82992a8a31f84e9c61491e5253594e4fcfeca92_1
AGCTCCAATAGCGTATACTAATGTTGTTGCAGTTAAAAGCTCGTAGTCGGATTTCAGTAGGCTCAATTAAGTCTCTATTCTGAGTTCTTTATGGGCCTGCTTCTTTGACAGAAACTTCTATGTTATTCATTTAGCGTGGGTAGCGACTGTCTCTTTTACTTTGAGAAAATTAGAGTGTTCAAAGCAGGTATTCGCCTGAATATTACTCTTGGAATAATGCTATAAGACTTTGGTTCTAATGTATTGGTGATTGGGACCAGAGTAATGATTGATAGGGACGGTTGGGGTCTTACGTGCTGCAAAGCGAGAGGTGAAATTCTTGGACCTTTGTATGACGAACAACTGCGAAAGCATTGGACTAGGACGTTCCCG
>daa093eb2cc81b4141c0df39c4fce197cc7cb20f_1
AGCTCCAATAGCGTATACTAATGTTGTTGCAGTTAAAAGACTCGTAGTACGGATTTCAGTAGGCTCAATTAAGTCTCTATTCTGAGTTCTTTATGGGCCTGCTTCTTTGACAGAAACTTCTATGTTATTCATTTAGCGTGGGTAGCGACTGTCTCTTTTACTTTGAGAAAATTAGAGTGTTCAAAGCAGGTATTCGCCTGAATATTACTCTTGGAATAATGCTATAAGACTTTGGTTCTAATGTATTGGTGATTGGGACCAGAGTAATGATTGATAGGGACGGTTGGGGTCTTACGTACTGCAAAGCGAGAGGTGAAATTCTTGGACCTTTGTATGCGAACAACTGCGAAAGCATTGGACTAGGACGTTCCCG
>dbadbe23a70d08a076b0e7e08037e78e20cd9439_1
AGCTCCAATAGCGTATACTAATGTTGTTGCAGTTAAAAGCTCGTAGTCGGATTTCAGTAGGCTCAATTAAGTCTCTATTCTGAGTTCTTTATGGGCCTGCTTCTTTGACAGAAACTTCTATGTTATTCATTTAGCGTGGGTAGCGACTGTCTCTTTTACTTTGAGAAAATTAGAGTGTTCAAAGCAGGTATTCGCCTGAATATTACTCTTGGAATAATGCTATAAGACTTTGGTTCTAATGTATTGGTGATTGGGACCAGAGTAATGATTGATAGGGACGGTTGAGGTCTTACGTACTGCAAAGCGAGAGGTGAAATTCTTGGACCTTTGTATGACGAACAACTGCGAAAGCATTGGACTAGGACGTTCCCG
>df722688df523a90ba6312cd98e12b02136e3324_1
AGCTCCAATAGCGTATACTAATGTTGTTGCAGTTAAAAGCTCGTAGTCGGATTTCAGTAGGCTCAATTAAGTCTCTATTCTGAGTTCTTTATGGGCCTGCTTCTTTGACAGAAACTTCTATGTTATTCATTTAGCGTGGGTAGCGACTGTCTCTTTTACTTTGAGAAAATTAGAGTGTTCAAAGCAGGTATTCGCCTGAATATTACTCTTGGAATAATGCTATAAGACTTTGGTTCTAATGTATTGGTGATTGGGACCAGAGTAATGATTGATAGGGACGGTTGGGCGTTTACGTACTGCAAAGCGAGAGGTGAAATTCTTGGACCTTTGTATGCGAACAACTGCGAAAGCATTGGACTAGGACGTTCCCG
>e13b70c389d7d87886624d34d16c807d031b6048_1
AGCTCCAATAGCGTATATTTAAGTTGTTGCAGTTAAAAGCTCGTAGTCGGATTTCAGTAGGCTCAGTTTAGTCTTCATTGTAAGATCTATATGGGCCTGCTTCTTTGACAGAAACTTCTATGTTATTCATTTAGCGTGGGCAGCGACTGTCTCTTTTACTTTGAGAAAATTAGAGTGTTCAAAGCAGGTAGATGCCTGAATATTACTCTTGGAATAATGCTATAAGACTTTGGTTCTAATGTATTGGTGATTGGGACCAGAGTAATGATTGATAGGGACGGTTGGGGTCTTACGTACTGCAAAGCGAGAGGTGAAATTCTTGGACCTTTGTATGACGAACAACTGCGAAAGCATTGGACTAGGACGTTCCCG
>e7a07fc0021b1af056ef5e9d2f063563a441b860_1
AGCTCCAATAGCGTATACTAATGTTGTTGCAGTTAAAAGCTCGTAGTCGGATTTCAGTAGGCTCAATTAAGTCTCTATTCTGAGTTCTTTATGGGCCTGCTTCTTTGACAGAAACTTCTATGTTATTCATTTAGCGTGGGTAGCGACTGTCTCTTTTACTTTGAGAAAATTAGAGTGTTCAAAGCAGGTATTCGCCAGAATATTACTCTTGGAATAATGCTATAAGACTTTGGTTCTAATGTATTGGTGATTGGGACCAGAGTAATGATTGATAGGGACGGTTGGGGTCTTACGTACTGCAAAGCGAGAGGTGAAATTCTTGGACCTTTGTATGCGAACAACTGCGAAAGCATTGGACTAGGACGTTCCCG
>e8dcbe21be848814ed52ba1223ff31de81db78b1_1
AGCTCCAATAGCGTATACTAATGTTGTTGCAGTTAAAAGCTCGTAGTCGGATTTCAGTAGGCTCAATTAAGTCTCTATTGTGAGTTCTTTATGGGCCTGCTTCTTTGACATAAACTTCTATGTTATTCATTTAGCGTGGGTAGCGACTGTCTCTTTTACTTTGAGAAAATTAGAGTGTTCAAAGCAGGTATTCGCCTGAATATTACTCTTGGAATAATGCTATAAGACTTTGGTTCTAATGTATTGGTGATTGGGACCAGAGTAATGATTGATAGGGACGGTTGGGGTCTTACGTACTGCAAGCGAGAGGTGAAATTCTTGGACCTTTGTATGACGAACAACTGCGAAAGCATTGGACTAGGACGTTCCCG
>f9bcc11a9c261273b15e0df186ade8decf84cb85_1
AGCTCCAATAGCGTATACTAATGTTGTTGCAGTTAAAAAGCTCGTAGTCGGATTTCAGTAGGCTCAATTAAGTCTCTATTCTGAGTTCTTTATGGGCCTGCTTCTTTGACAGAAACTTCTATGTTATTCATTTAGCGTGGGTAGCGACTGTCTCTTTTACTTTGAGAAAATTAGAGTGTTCAAAGCAGGTATTCGCCTGAATATTACTCTTGGAATAATGCTATAAGACTTTGGTTCTAATGTATTGGTGATTGGGACCAGAGTAATGATTGATAGGGACGGTTGGGGTCTTACGTACTACAAAGCGAGAGGTGAAATTCTTGGACCTTTGTATGCGAACAACTGCGAAAGCATTGGACTAGGACGTTCCCG
>fb49b68cddeee7c7e78876fe1f2401048a3edd03_1
AGCTCCAATAGCGTATACTAATGTTGTTGCAGTTAAAAAGCTCGTAGTCGGATTTCAGTAGGCTCAATTAAGTCTCTATTCTGAGTTCTTTATGGGCCTGCTTCTTTGACAGAAACTTCTATGTTATTCATTTAGCGTGAGTAGCGACTGTCTCTTTTACTTTGAGAAAATTAGAGTGTTCAAAGCAGGTATTCGCCTGAATATTACTCTTGGAATAATGCTATAAGACTTTGGTTCTAATGTATTGGTGATTGGGACCAGAGTAATGATTGATAGGGACGGTTGGGGTCTTACGTACTGCAAAGCGAGAGGTGAAATTCTTGGACCTTTGTATGACGAACAACTGCGAAAGCATTGGACTAGGACGTTCCCG

# Ei45_V9_radiolaria.fas
>599a9d1ab5ed3e477599254458c741b14dac1691_508
GTCGCTCCTACCGATTGGATGATTCGGTAAGCTTTTGGGATTGATTGCGTATTTCTCATTGAGGGTACGTTAAAACAACTTAATCAAACCTAATCATCTAGAGGAAGGAGAAGTCGTAACAAGGTTTCC
>ffdd6b43a06e827c764c0b8916ee3d58a3663928_45
GTCGCTCCTACCGATTGGATGATTCGGTAAGCTTTTGGGATTGATTAGCTACTTGTCACTGACATTAGCTTTTAACAACTTAATCAAACCTAATCATCTAGAGGAAGGAGAAGTCGTAACAAGGTTTCC
>f85f078e9dc0ab7aa71e9d679471f24ea11a4045_21
GTCGCTCCTACCGATTGGATGATTCGGTAAGCTTTTGGGTATTGATTGCGTATTTCTCATTGAGGGTACGTTAAAACAACTTAATCAAACCTAATCATCTAGAGGAAGGAGAAGTCGTAACAAGGTTTCC
>50b3429dc679a1109ee546755c26b0aa7c957b2d_1
GTCGCTCCTACCGATTGGATGATTCGGTAAGCTTTTGGGATTGATTAGCTACTTGTCACTGACATTAGCTTTTAAACAACTCAATCAAACCTAATCATCTAGAGGAAGGAGAAGTCGTAACAAGGTTTCC
>7622cf30df5a50940ea5dd4da768562c247942a6_1
GTCGCTCCTACCGATTGGATGATTCGGTAAGCTTTTGGGATTGATTATGTATCTGTCATTGATGGTACATTTTAACAACTTAATCAAACCTAATCATCTAGAGGAAGGAGAAGTCGTAACAAGGTTTCC
>e9bb8b1079433b4eaa52fc838e09d628081479db_1
GTCGCTCCTACCGATTGGATGATTCGGTAAGCTTTTGGGATTGATTGCATATTTCTCATTGAGGGTACGTTAAAACAACTTAATCAAACCTAATCATCTAGAGGAAGGAGAAGTCGTAACAAGGTTTCC

# PEC16_1_V4_radiolaria.fas
>7cbe8476b88f679dc57c475ba8175642dcf38be9_3
AGCTCCAATAGCGTATACTAATGTTGTTGCAGTTAAAAGACTCGTAGTCGGATTTCAGTATTAGGCCCTTCGCTTCCAAATGGTTGCTTGTGGTCTTTACTTCCTTAACAGAATCTTTCATCTCATTAATTTGTGGTGTTTGGGGCCTGTTTCTTTTACTTTGAGAAAATTAGAGTGTTCAAAGCGGGTTTTTCGCCTGAATATTACTCTTGGAATAATAATATAGGACTTTGGTTCTTTTTGTTGGTGACTTAGAACCGAAGTAATGATTGATAGGGACAGTTGGGGTCATTCGTACTATGAAGCGAGAGGTGAAATTCATGGACCTTTGTATGACGAACTACTGCGAAAGCATTTGACAAGGTGTTCCCG
>d097489fc81e77ee3c6c08c8c19ce42a714fe8b1_3
AGCTCCAATAGCGTATACTAATGTTGTTGCAGTTAAAAGCTCGTAGTCGGATTTCAGTATTAGGCCCTTCGCTTCCAAATGGTTGCTTGTGGTCTTTACTTCCTTAACAGAATCTTTCATCTCATTAATTTGTGGTGTTTGGGGCCTGTTTCTTTTACTTTGAGAAAATTAGAGTGTTCAAAGCGGGTTTTTCGCCTGAATATTACTCTTGGAATAATAATATAGGACTTTGGTTCTTTTTGTTGGTGACTTAGAACCGAAGTAATGATTGATAGGGACAGTTGGGGTCATTCGTACTATGAAGCGAGAGGTGAAATTCATGGACCTTTGTATGACGAACTACTGCGAAAGCATTTGACAAGGTGTTCCCG
>0bb25704d552eeca44d268a76eb4e2f964e01bb9_2
AGCTCCAATAGCATATACTAATGTTGTTGCAGTTAAAAGCTCGTAGTCGGATTTCAGTATTAGGCCCTTCGCTTCCAAATGGTTGCTTGTGGTCTTTACTTCCTTAACAGAATCTTTCATCTCATTAATTTGTGGTGTTTGGGGCCTGTTTCTTTTACTTTGAGAAAATTAGAGTGTTCAAAGCGGGTTTTCGCCTGAATATTACTCTTGGAATAATAATATAGGACTTTGGTTCTTTTTGTTGGTGACTTAGAACCGAAGTAATGATTGATAGGGACAGTTGGGGTCATTCGTACTATGAAGCGAGAGGTGAAATTCATGGACCTTTGTATGACGAACTACTGCGAAAGCATTTGACAGGATGTTCCCG
>099acd0fe983c804cb8617fc93e70add89edce00_1
AGCTCCAATAGCGTATACTAATGTTGTTGCAGTTAAAAGCTCGTAGTCGGATTTCAGTATTAGGCCCTTCGCTTCCAAATGGTTGCTTGTGGTCTTTACTTCCTTAACAGAATCTTTCATCTCATTAATTTGTGGTGTTTGGGGCCTGTTTCTTTTACTTTGAGAAAATTAGAGTGTTCAAAGCGGGTTTTTCGCCTGAATATTACTCTTGGAATAATAATATAGGACTTTGGTTCTTTTTGTTGGTGACTTAGAACCGAAGTAATGATTGATAGGGACAGTTGGGGTCATTCGTACTATGAAGCGAGAGGTGAAATTCATGGACCTTTGTATGACGAACTACTGCGAAAGCATTTGACAAGGATGTTCCCG
>244803ec375bcf73b462819451fca7e7a4d1ff80_1
AGCTCCAATAGCGTATACTAATGTTGTTGCAGTTAAAAGCTCGTAGTCGGATTTCAGTATTAGGCCCTTCGCTTCCAAATGGTTGCTTGTGGTCTTTACTTCCTTAACAGAATCTTTCATCTCATTAATTTGTGGTGTTTGGGGCCTGTTTCTTTTACTTTGAGAAAATTAGAGTGTTCAAAGCGGGTTTTCGCCTGAATATTACTCTTGGAATAATAATATAGGACTTTGGTTCTTTTTGTTGGTGACTTAGAACCGAAGTAATGATTGATAGGGACAGTTGGGGTCATTCGTACTATGAAGCGAGAGGTGAAATTCATGGACCTTTGTATGACGAACTACTGCGAAAGCATTTGACAGGATGTTCCCG
>839f687a543c6721e3464c77c050362c5a094928_1
AGCTCCAATAGCGTATACTAATGTTGTTGTAGTTAAAAGCTCGTAGTCGGATTTCAGTATTAGGCCCTTCGCTTCCAAATGGTTGCTTGTGGTCTTTACTTCCTTAACAGAATCTTTCATCTCATTAATTTGTGGTGTTTGGGGCCTGTTTCTTTACTTTGAGAAAATTAGAGTGTTCAAAAGCGGGTTTTTCGCCTGAATATTACTCTTGGAATAATAATATAGGACTTTGGTTCTTTTTGTTGGTGACTTAGAACCGAAGTAATGATTGATAGGGACAGTTGGGGTCATTCGTACTATGAAGCGAGAGGTGAAATTCATGGACCTTTGTATGACGAACTACTGCGAAAGCATTTGACAAGGATGTTCCCG
>a145f7148b7f0ffebd808333370579f15a5cda39_1
AGCTCCAATAGCGTATACTAATGTTGTTGCAGTTAAAAGCTCGTAGTCGGATTTCAGTATTAGGCCCTTCGCTTCCAAATGGTTGCTTGTGGTCTTTACTTCCTTAACAGAATCTTTCATCTCATTAATTTGTGGTGTTTGGGGCCTGTTTCTTTACTTTGAGAAAATTAGAGTGTTCAAAGCGGGTTTTTCGCCTGAATATTACTCTTGGAATAATAATATAGGACTTTGGTTCTTTTGTTGGTGACTTAGAACCGAAGTAATGATTGATAGGGACAGTTGGGGTCATTCGTACTATGAAGCGAGAGGTGAAATTCATGGACCTTTGTATGACGAACTACTGCGAAAGCATTTGACAAGGATGTTCCCG
>fc94522c09bf4b775ad05d704b35796c25366cf8_1
AGCTCCAATAGCGTATACTAATGTTGTTGCAGTTAAAAGCTCGTAGTCGGATTTCAGTATTAGGCCCTTCGCTTCCAAATGGTTGCTTGTGGTCTTTACTTCCTTAACAGAATCTTTCATCTCATTAATTTGTGGTGTTTGGGGCCTGTTTCTTTACTTTGAGAAAAATTAGAGTGTTCAAAGCGGGTTTTTCGCCTGAATATTACTCTTGGAATAATAATATAGGACTTTGGTTCTTTTTGTTGGTGACTTAGAACCGAAGTAATGATTGATAGGGACAGTTGGGGTCATTCGTACTATGAAGCGAGAGGTGAAATTCATGGACCTTTGTATGACGAACTACTGCGAAAGCATTTGACAAGGATGTTCCCG

# PEC16_1_V9_radiolaria.fas
>7cf96d639880da99016506f593bbed1b05693298_829
GTCGCTCCTACCGATTGGATGATTCGGTAAGCTCTTGGGATTGATTGACGACCTGCATGTCAGACGGATGTTGACAACTTGATCAAACCTAATCATCTAGAGGAAGGAGAAGTCGTAACAAGGTTTCC
>4971d07e41d8c63b3aab8546a58a4953b5aeb3da_5
GTCGCTCCTACCGATCGGATAAGTTAGTGATTGAATTAGATGAGGAGTTAACTTACTGATAGACAACATTACGATTAAAATTTGCAAACTAGATTATTTAGAGGAAGGAGAAGTCGTAACAAGGTTTCC
>b7ada2fb38a7c42ba87b42ba4796f6bb65af0857_3
GTCGCTCCTACCGATTGGATGAGTTGGTGAGTGGATTGGAGTAATAGCTAACTTCTTTAACAGATGGTAGTATTTGTAAGATTTGCAAACTAGATTATCTAGAGGAAGGAGAAGTCGTAACAAGGTTTCC
>01bfb51a36d44f808cdf8d7f6cfcb73f51ef7daf_1
GTCGCTCCTACCGATTGGATGATTCGGTAAGCTCTTGGGATTGATTGATGACCTGCATGTCAGACGGATGTTGACAACTTGATCAAACCTAATCATCTAGAGGAAGGAGAAGTCGTAACAAGGTTTCC
>2b23b8d976028bc3f6a20cd03923bc38255ea997_1
GTCGCTCCTACCGATTGGATGATTCGGTAAGCTCTTGGGATTGATTGACGACCTGCATGTCAGACGGATGTTGACAACTTGATCAAACCTAATCATCTAGAGGAAGGAGAAGTCATAACAAGGTTTCC
>6e73a3bed220f91173de0a2b334a50b4e172ba3b_1
GTCGCTCCTACCGATTGGATGATTCGGTAAGCTCTTGGGATTGATTGACGACCTGCATTTCAGACGGATGTTGACAACTTGATCAAACCTAATCATCTAGAGGAAGGAGAAGTCGTAACAAGGTTTCC
>bd577cd7cf3fb89ddf2ec28d4e5f903545b6cb4c_1
GTCGCTCCTACCGATTGGATGAGTTGGTGAGTGGATTGGAGTAATAGCTAACTTCTTTAACAGATGGTAGTATTTGTAAGATTTGCAAACTAGATTATCTAAGAGGAAGGAGAAGTCGTAACAAGGTTTCC
>c6bfb286a6b6fc362e9be3524f05f1f8dcc432d1_1
GTCGCTCCTACCGATTGGATGAGTTGGTGAGTGGATTGGAGTAATAGCTATCTTCTTTAACAGATGGTAGTATTTTTAAGATTTGCAAACTAGATTATCTAGAGGAAGGAGAAGTCGTAACAAGGTTTCC

# PEC16_2_V4_radiolaria.fas
# No amplicon

# PEC16_2_V9_radiolaria.fas
>7cf96d639880da99016506f593bbed1b05693298_779
GTCGCTCCTACCGATTGGATGATTCGGTAAGCTCTTGGGATTGATTGACGACCTGCATGTCAGACGGATGTTGACAACTTGATCAAACCTAATCATCTAGAGGAAGGAGAAGTCGTAACAAGGTTTCC
>2b23b8d976028bc3f6a20cd03923bc38255ea997_1
GTCGCTCCTACCGATTGGATGATTCGGTAAGCTCTTGGGATTGATTGACGACCTGCATGTCAGACGGATGTTGACAACTTGATCAAACCTAATCATCTAGAGGAAGGAGAAGTCATAACAAGGTTTCC
>4c81a94f3a2586c97d0f60241fceeb3ff2f26678_1
GTCGCCCCTACCGATTGGATGATTCGGTAAGCTCTTGGGATTGATTGACGACCTGCATGTCAGACGGATGTTGACAACTTGATCAAACCTAATCATCTAGAGGAAGGAGAAGTCGTAACAAGGTTTCC
>822b91aa09739257370aab1cee7e9268ea5b1d86_1
GTCGCTCCTACCGATTGGATGATTCGGTAAGCTCTTGGGATTGATTGTCGACCTGCATGTCAGACGGATGTTGACAACTTGATCAAACCTAATCATCTAGAGGAAGGAGAAGTCGTAACAAGGTTTCC
>824541b8994aef3e069ab9c71096a4b835def43d_1
GTCGCTCCTACCGATTGGATGATTCGGTAAGCTCTTGGGATTGATTGACGACCTGCGTGTCAGACGGATGTTGACAACTTGATCAAACCTAATCATCTAGAGGAAGGAGAAGTCGTAACAAGGTTTCC
>b1eb31f132e09ffb212b37848cd9924e6c85bd53_1
GTCGCTCCTACCGATTGGATGATTCGGTAAGCTCTTGGGATTGATTGACGACCTGCATGTCAGACGGATGTTGACAACTTGATCAAACCTAATCAATCTAGAGGAAGGAGAAGTCGTAACAAGGTTTCC
>bdd5b19e761779826f287c4c4534ff7903d40dd9_1
GTCGCTCCTACCGTTTGGTATGATTCGGTAAGCTCTTGGGATTGATTGACGACCTGCATGTCAGACGGATGTTGACAACTTGATCAAACCTAATCATCTAGAGGAAGGAGAAGTCGTAACAAGGTTTCC

# Vil32_V4_radiolaria.fas
>b2cdfbbbb91be4e608d9aa9e980ffff5dbc3d9f8_372
AGCTCCAATAGCGTATACTAATGTTGTTGCAGTTAAAAGCTCGTAGTCGGATTTCAGTTTGATTTAGGATCTTCTACCCGGAGGCCCTTGATCATTCTTCCTTGACATAAACAGCCATGTCCTTCATTGGATGTAGTTTGGGAGTGTCTCTTTTACTTTGAGAAAATTAGAGTGTTCAAAGCAGGTAATCGCCTGAATATTACTCTTGGAATAATACTATAGGACTTTGGTTCTGCATTGTTGGTGTTCAGAGCCAGAGTAATGATTGATAGGGACGGTTGGGGTCATTAGTACTGCGAAGCGAGAGGTGAAATTCTTGGACCTTTGTATGACTAACAACTGCGAAAGCATTTGACTAGGACGTTCCCG
>50c0b8a997e85bec315e300f12a5a1ece16b0934_8
AGCTCCAATAGCGTATACTAATGTTGTTGCAGTTAAAAGACTCGTAGTCGGATTTCAGTTTGATTTAGGATCTTCTACCCGGAGGCCCTTGATCATTCTTCCTTGACATAAACAGCCATGTCCTTCATTGGATGTAGTTTGGGAGTGTCTCTTTTACTTTGAGAAAATTAGAGTGTTCAAAGCAGGTAATCGCCTGAATATTACTCTTGGAATAATACTATAGGACTTTGGTTCTGCATTGTTGGTGTTCAGAGCCAGAGTAATGATTGATAGGGACGGTTGGGGTCATTAGTACTGCGAAGCGAGAGGTGAAATTCTTGGACCTTTGTATGACTAACAACTGCGAAAGCATTTGACTAGGACGTTCCCG
>1a32b12bbe5671533ed741e008ef1e857798be76_4
AGCTCCAATAGCGTATACTAATGTTGTTGCAGTTAAAAGCTCGTAGTCGGATTTCAGTTTGATTTAGGATCTTCTACCCGGAGGCCCTTGATCATTCTTCCTTGACATAAACAGCCATGTCCTTCATTGGATGTAGTTTGGGTAGTGTCTCTTTTACTTTGAGAAAATTAGAGTGTTCAAAGCAGGTAATCGCCTGAATATTACTCTTGGAATAATACTATAGGACTTTGGTTCTGCATTGTTGGTGTTCAGAGCCAGAGTAATGATTGATAGGGACGGTTGGGGTCATTAGTACTGCGAAGCGAGAGGTGAAATTCTTGGACCTTTGTATGACTAACAACTGCGAAAGCATTTGACTAGGACGTTCCCG
>107ddefd0ec71068993e9a6a84067e4bdf14143a_1
AGCTCCAATAGCGTATACTAATGTTGTTGCAGTTAAAAGCTCGTAGTCGGATTTCTGTTTGATTTAGGATCTTCTACCCGGAGGCCCTTGATCATTCTTCCTTGACATAAACAGCCATGTCCTTCATTGGATGTAGTTTGGGAGTGTCTCTTTTACTTTGAGAAAATTAGAGTGTTCAAAGCAGGTAATCGCCTGAATATTACTCTTGGAATAATACTATAGGACTTTGGTTCTGCATTGTTGGTGTTCAGAGCCAGAGTAATGATTGATAGGGACGGTTGGGGTCATTAGTACTGCGAAGCGAGAGGTGAAATTCTTGGACCTTTGTATGACTAACAACTGCGAAAGCATTTGACTAGGACGTTCCCG
>1a231dd9d349eef84b0d894d6d9d519bc1468c8f_1
AGCTCCAATAGCGTATACTAATGTTGTTGCAGTTAAAAGCTCGTAGTCGGATTTCAGTTTGATTTAGGATCTTCTACCCGGAGGCCCTTGATCATTCTTCCTTGACATAAACAGCCATGTCCTTCATTGGATGTAGTTTGGGTAGTGTCTCTTTTACTTTGTAGAAAATTAGAGTGTTCAAAGCAGGTAATCGCCTGAATATTACTCTTGGAATAATACTATAGGACTTTGGTTCTGCATTGTTGGTGTTCAGAGCCAGAGTAATGATTGATAGGGACGGTTGGGGTCATTAGTACTGCGAAGCGAGAGGTGAAATTCTTGGACCTTTGTATGACTAACAACTGCGAAAGCATTTGACTAGGACGTTCCCG
>38c106eb4f8355d7590dd402ab55343cea1643c5_1
AGCTCCAATAGCGTATACTAATGTTGTTGCAGTTAAAAGCTCGTAGTCGGATTTCAGTTTGATTTAGGATCTTCTACCCGGAGGCCCTTGATCATTCTTCCTTGACATAAACAGCCATGTCCTTCATTGGATGTAGTTTGGGAGTGTCTCTTTTACTTTGAGAAAATTAGAGTGTTCAAAGTAGGTAATCGCCTGAATATTACTCTTGGAATAATACTATAGGACTTTGGTTCTGCATTGTTGGTGTTCAGAGCCAGAGTAATGATTGATAGGGACGGTTGGGGTCATTAGTACTGCGAAGCGAGAGGTGAAATTCTTGGACCTTTGTATGACTAACAACTGCGAAAGCATTTGACTAGGACGTTCCCG
>3f0255b3296b666e1200426d4e252d4395d3a565_1
AGCTCCAATAGCGTATACTAATGTTGTTGCAGTTAAAAGCTCGTAGTCGGATTTCAGTTTGATTTAGGATCTTCTACCCGGAGGCCCTTGATCATTCTTCCTTGACATAAACAGCCATGTCCTTCATTGGATGTAGTTTGGGAGTTTCTCTTTTACTTTGAGAAAATTAGAGTGTTCAAAGCAGGTAATCGCCTGAATATTACTCTTGGAATAATACTATAGGACTTTGGTTCTGCATTGTTGGTGTTCAGAGCCAGAGTAATGATTGATAGGGACGGTTGGGGTCATTAGTACTGCGAAGCGAGAGGTGAAATTCTTGGACCTTTGTATGACTAACAACTGCGAAAGCATTTGACTAGGACGTTCCCG
>43d06c4b6f0cb2ee8dbfcd8c9956aa53880d76d2_1
AGCTCCAATAGCGTATATTAAAGTTGTTGCAGTTAAAAGCTCGTAGTCGGATTTCAGTTTGATTTAGGATCTTCTACCCGGAGGCCCTTGATCATTCTTCCTTGACATAAACAGCCATGTCCTTCATTGGATGTAGTTTGGGAGTGTCTCTTTTACTTTGAGAAAATTAGAGTGTTCAAAGCAGGTAATCGCCTGAATATTACTCTTGGAATAATACTATAGGACTTTGGTTCTGCATTGTTGGTGTTCAGAGCCAGAGTAATGATTGATAGGGACGGTTGGGGTCATTAGTACTGCGAAGCGAGAGGTGAAATTCTTGGACCTTTGTATGACTAACAACTGCGAAGCATTTGACTAGGACGTTCCCG
>4f23df3236e36e39ba24f22c0dd7beadd32fbb83_1
AGCTCCAATAGCGTATACTAATGTTATTGCAGTTAAAAGCTCGTAGTCGGATTTCAGTTTGATTTAGGATCTTCTACCCGGAGGCCCTTGATCATTCTTCCTTGACATAAACAGCCATGTCCTTCATTGGATGTAGTTTGGGAGTGTCTCTTTTACTTTGAGAAAATTAGAGTGTTCAAAGCAGGTAATCGCCTGAATATTACTCTTGGAATAATACTATAGGACTTTGGTTCTGCATTGTTGGTGTTCAGAGCCAGAGTAATGATTGATAGGGACGGTTGGGGTCATTAGTACTGCGAAGCGAGAGGTGAAATTCTTGGACCTTTGTATGACTAACAACTGCGAAAGCATTTGACTAGGACGTTCCCG
>5aad3d72ce1c5b6abc25e8864a394ba9905f21b8_1
AGCTCCAATAGCGTATACTAATGTTGTTGTAGTTAAAAGCTCGTAGTCGGATTTCAGTTTGATTTAGGATCTTCTACCCGGAGGCCCTTGATCATTCTTCCTTGACATAAACAGCCATGTCCTTCATTGGATGTAGTTTGGGAGTGTCTCTTTTACTTTGAGAAAATTAGAGTGTTCAAAGCAGGTAATCGCCTGAATATTACTCTTGGAATAATACTATAGGACTTTGGTTCTGCATTGTTGGTGTTCAGAGCCAGAGTAATGATTGATAGGGACGGTTGGGGTCATTAGTACTGCGAAGCGAGAGGTGAAATTCTTGGACCTTTGTATGACTAACAACTGCGAAAGCATTTGACTAGGACGTTCCCG
>6370fcdd1b7490ee9b18a2901ba91dd18de4ef73_1
AGCTCCAATAGCGTATACTAATGTTGTTGCAGTTAAAAGCTCGTAGTCGGATTTCAGTTTGATTTAGGATCTTCTACTCGGAGGCCCTTGATCATTCTTCCTTGACATAAACAGCCATGTCCTTCATTGGATGTAGTTTGGGAGTGTCTCTTTTACTTTGAGAAAATTAGAGTGTTCAAAGCAGGTAATCGCCTGAATATTACTCTTGGAATAATACTATAGGACTTTGGTTCTGCATTGTTGGTGTTCAGAGCCAGAGTAATGATTGATAGGGACGGTTGGGGTCATTAGTACTGCGAAGCGAGAGGTGAAATTCTTGGACCTTTGTATGACTAACACTGCGAAAGCATTTGACTAGGACGTTCCCG
>65742f92809612c9b8ae658bec573dbeb01f81e1_1
AGCTCCAATAGCGTATACTAATGTTGTTGCAGTTAAAAGCTCGTAGTCGGATTTCAGTTTGATTTAGGATCTTCTACCCGGAGGCCCTTGATCATTCTTCCTTGACATAAACAGCCATGTCCTTCATTGGATGTAGTTTGGGAGTGTCTCTTTTACTTTGAGAAAATTAGAGTGTTCAAAGCAGGTAATCGCCTGAATATTACTCTTGGAATAATACTATAGGACTTTGGTTCTGCATTGTTGGTGTTCAGAGCCAGAGTAATGATTGATAGGGACGGTTGGGGTCATTAGTACTACGAAGCGAGAGGTGAAATTCTTGGACCTTTGTATGACTAACAACTGCGAAAGCATTTGACTAGGACGTTCCCG
>72c4ed51d1b7afc6831a1ff185d243cb8b259021_1
AGCTCCAATAGCGTATACTAATGTTGTTGCAGTTAAAAGCTCGTAGTCGGATTTCAGTTTGATTTAGGATCTTCTGCCCGGAGGCCCTTGATCATTCTTCCTTGACATAAACAGCCATGTCCTTCATTGGATGTAGTTTGGGAGTGTCTCTTTTACTTTGAGAAAATTAGAGTGTTCAAAGCAGGTAATCGCCTGAATATTACTCTTGGAATAATACTATAGGACTTTGGTTCTGCATTGTTGGTGTTCAGAGCCAGAGTAATGATTGATAGGGACGGTTGGGGTCATTAGTACTGCGAAGCGAGAGGTGAAATTCTTGGACCTTTGTATGACTAACACTGCGAAAGCATTTGACTAGGACGTTCCCG
>74c8e4c378b6172ada5fd1466ceb1725aa9c03ee_1
AGATCCAATAGCGTATACTAATGTTGTTGCAGTTAAAAGCTCGTAGTCGGATTTCAGTTTGATTTAGGATCTTCTACCCGGAGGCCCTTGATCATTCTTCCTTGACATAAACAGCCATGTCCTTCATTGGATGTAGTTTGGGAGTGTCTCTTTTACTTTGAGAAAATTAGAGTGTTCAAAGCAGGTAATCGCCTGAATATTACTCTTGGAATAATACTATAGGACTTTGGTTCTGCATTGTTGGTGTTCAGAGCCAGAGTAATGATTGATAGGGACGGTTGGGGTCATTAGTACTGCGAAGCGAGAGGTGAAATTCTTGGACCTTTGTATGACTAACAACTGCGAAAGCATTTGACTAGGACGTTCCCG
>7e5dfd9d5b99a0a9511cbcf2e78d26b6255d9464_1
AGCTCCAATAGCGTATACTAATTTTGTTGCAGTTAAAAGCTCGTAGTCGGATTTCAGTTTGATTTAGGATCTTCTACCCGGAGGCCCTTGATCATTCTTCCTTGACATAAACAGCCATGTCCTTCATTGGATGTAGTTTGGGAGTGTCTCTTTTACTTTGAGAAAATTAGAGTGTTCAAAGCAGGTAATCGCCTGAATATTACTCTTGGAATAATACTATAGGACTTTGGTTCTGCATTGTTGGTGTTCAGAGCCAGAGTAATGATTGATAGGGACGGTTGGGGTCATTAGTACTGCGAAGCGAGAGGTGAAATTCTTGGACCTTTGTATGACTAACAACTGCGAAAGCATTTGACTAGGACGTTCCCG
>b72155bf5503436d99a5e447fc15c6bf768526fb_1
AGCTCCAATAGCGTATACTAATGTTGTTGCAGTTAAAAGCTCGTAGTCGGATTTCAGTTTGATTTAGGATCTTCTACCCGGAGGCCCTTGATCATTCTTCCTTGACATAAACAGCCATGTCCTTCATTGGATGTAGTTTGGGAGTGTCTCTTTTACTTTGAGAAAATTAGAGTGTTCAAAGCAGGTAATCGCCTGAATATTACTCTTGGAATAATACTATAGGACTTTGGTTCTGCATTGTTGGTGTTCAGAGCCAGAGTAATGATTGATAGGGACGGTTGGAGTCATTAGTACTGCGAAGCGAGAGGTGAAATTCTTGGACCTTTGTATGACTAACACTGCGAAAGCATTTGACTAGGACGTTCCCG
>bb71e859922bbd766d22331f74c1bbc852addeb2_1
AGCTCCAATAGCGTATACTAATGTTGTTGCAGTTAAAAGCTCGTAGTCGGATTTCAGTTTGATTTAGGATTTTCTACCCGGAGGCCCTTGATCATTCTTCCTTGACATAAACAGCCATGTCCTTCATTGGATGTAGTTTGGGAGTGTCTCTTTTACTTTGAGAAAATTAGAGTGTTCAAAGCAGGTAATCGCCTGAATATTACTCTTGGAATAATACTATAGGACTTTGGTTCTGCATTGTTGGTGTTCAGAGCCAGAGTAATGATTGATAGGGACGGTTGGGGTCATTAGTACTGCGAAGCGAGAGGTGAAATTCTTGGACCTTTGTATGACTAACAACTGCGAAAGCATTTGACTAGGACGTTCCCG
>fde4abc2e3574c7064540afadf44927a79904c00_1
AGCTCCAATAGCGTATACTAATGTTGTTGCAGTTAAAAGACTCGTAGTCGGATTTCAGTTTGATTTAGGATCTTCTACCCGGAGGCCCTTGATCATTCTTCCTTGACATAAACAGCCATGTCCTTCATTGGATGTAGTTTGGGTAGTGTCTCTTTTACTTTGAGAAAATTAGAGTGTTCAAAGCAGGTAATCGCCTGAATATTACTCTTGGAATAATACTATAGGACTTTGGTTCTGCATTGTTGGTGTTCAGAGCCAGAGTAATGATTGATAGGGACGGTTGGGGTCATTAGTACTGCGAAGCGAGAGGTGAAATTCTTGGACCTTTGTATGACTAACAACTGCGAAAGCATTTGACTAGGACGTTCCCG

# Vil32_V9_radiolaria.fas
>b55341033f27c643b0758f4eed7bc8f5fb5258ce_1037
GTCGCTCCTACCGATTGGATGATTCGGTAAGCTCTTGGGATAGACTGTTCAGTAGTCATGTATTACTTTACTGGTTCAAAGCCTGATCAAACCTAATCATCTAGAGGAAGGAGAAGTCGTAACAAGGTTTCC
>fc5812f0a8d0dc8108476eefe26063f9c529b645_2
GTCGCTCCTACCGATTGGATGAGTTGGTGAGTGGATTGGAGTAACAGCAATGTTCCTGTAAGATTGTTGTATTTTTAAAAATTTGCAAACTAGATTATCTAGAGGAAGGAGAAGTCGTAACAAGGTTTCC
>a2058947e9d0e0a153f24742e1ab3fbe9efd243b_1
GTCGCTCCTACCGATTGAATGAGTTGGTGAGTGAATTGGAGCGACGGCTATCTTGCAAAAAGATTATTGTGATATTTTAAAATTTACAAACTAGATTATTTAGAGGAAGGAGAAGTCGTAACAAGGTTTCC
```

Author: Frédéric Mahé
<mahe@rhrk.uni-kl.de>

Date: [2014-04-07 lun.]

HTML generated by org-mode 6.33x in emacs 23
